# Cell-Type-Specific Gene Modules Related to the Regional Homogeneity of Spontaneous Brain Activity and Their Associations With Common Brain Disorders

**DOI:** 10.3389/fnins.2021.639527

**Published:** 2021-04-20

**Authors:** Junlin Shen, Bingbing Yang, Zhonghua Xie, Heng Wu, Zhanye Zheng, Jianhua Wang, Ping Wang, Peng Zhang, Wei Li, Zhaoxiang Ye, Chunshui Yu

**Affiliations:** ^1^Department of Radiology and Tianjin Key Laboratory of Functional Imaging, Tianjin Medical University General Hospital, Tianjin, China; ^2^Department of Mathematics, School of Science, Tianjin University of Science and Technology, Tianjin, China; ^3^Tianjin Key Laboratory of Lung Cancer Metastasis and Tumor Microenvironment, Tianjin Lung Cancer Institute, Tianjin Medical University General Hospital, Tianjin, China; ^4^Department of Pharmacology, School of Basic Medical Science, Tianjin Medical University, Tianjin, China; ^5^School of Medical Imaging and Tianjin Key Laboratory of Functional Imaging, Tianjin Medical University, Tianjin, China; ^6^Department of Radiology, National Clinical Research Center for Cancer, Tianjin Medical University Cancer Institute and Hospital, Tianjin, China; ^7^Key Laboratory of Cancer Prevention and Therapy, Tianjin, China; ^8^Tianjin’s Clinical Research Center for Cancer, Tianjin, China

**Keywords:** gene expression, Allen Human Brain Atlas, cell type, regional homogeneity, fMRI

## Abstract

Mapping gene expression profiles to neuroimaging phenotypes in the same anatomical space provides opportunities to discover molecular substrates for human brain functional properties. Here, we aimed to identify cell-type-specific gene modules associated with the regional homogeneity (ReHo) of spontaneous brain activity and their associations with brain disorders. Fourteen gene modules were consistently associated with ReHo in the three datasets, five of which showed cell-type-specific expression (one neuron-endothelial module, one neuron module, one astrocyte module and two microglial modules) in two independent cell series of the human cerebral cortex. The neuron-endothelial module was mainly enriched for transporter complexes, the neuron module for the synaptic membrane, the astrocyte module for amino acid metabolism, and microglial modules for leukocyte activation and ribose phosphate biosynthesis. In enrichment analyses of cell-type-specific modules for 10 common brain disorders, only the microglial module was significantly enriched for genes obtained from genome-wide association studies of multiple sclerosis (MS) and Alzheimer’s disease (AD). The ReHo of spontaneous brain activity is associated with the gene expression profiles of neurons, astrocytes, microglia and endothelial cells. The microglia-related genes associated with MS and AD may provide possible molecular substrates for ReHo abnormality in both brain disorders.

## Introduction

Resting-state functional magnetic resonance imaging (rs-fMRI) has been widely used to assess spontaneous brain activity, which records the blood oxygen level-dependent (BOLD) fluctuations during rest. Regional homogeneity (ReHo) is a measure reflecting the degree of local synchronization that occurs over the course of the rs-fMRI, that is, the similarity of the fluctuation of BOLD signals of a given voxel with those of its nearest neighbors (Zang et al., 2004; Zuo et al., 2013), which is measured by Kendall’s coefficient of concordance (KCC). Abnormal ReHo may be associated with pathological changes in the brain caused by specific neuropsychitric diseases (He et al., 2007; Wu et al., 2009; You et al., 2011). We were particularly interested in this measure for three reasons: (a) as a data-driven method, ReHo does not require an *a priori* hypothesis, which is appropriate for exploratory analysis; (b) the test-retest reliability of ReHo is well established. With a popular acquisition and preprocessing pipeline, ReHo has been demonstrated to be a highly robust and reliable index for mapping the local activity of the human functional connectome (Zuo et al., 2013; Zuo and Xing, 2014); and (c) ReHo has been used to identify brain functional abnormalities in many brain disorders, such as Alzheimer’s disease (AD) (He et al., 2007; Zhang et al., 2012), Parkinson’s disease (PD) (Zeng et al., 2017; Liu et al., 2019), epilepsy (EP) (Zeng et al., 2013, 2015), stroke (Liu et al., 2014; Zhao et al., 2018), multiple sclerosis (MS) (Dogonowski et al., 2013; Wu et al., 2016), bipolar disorder (BP) (Yao et al., 2018; Liu et al., 2020), major depressive disorder (MDD) (Guo et al., 2011; Sun et al., 2018), schizophrenia (SCZ) (Xu et al., 2015; Wang et al., 2018), autism spectrum disorders (ASD) (Paakki et al., 2010; Shukla et al., 2010), and attention deficit hyperactivity disorder (ADHD) (Cao et al., 2006; Wang et al., 2013). Although candidate gene studies in healthy and diseased populations have indicated the genetic bases of ReHo (Yu et al., 2014; Zheng et al., 2017; Gou et al., 2018; Shang et al., 2019), the molecular substrates underlying the ReHo of spontaneous brain activity remain elusive.

Genome-wide association studies (GWASs) of neuroimaging phenotypes suggest that resting-state brain functional phenotypes derived from both fMRI (Elliott et al., 2018) and electroencephalogram (Jawinski et al., 2019) are heritable, although the latter did not find genome-wide significant hit due to small sample size (*n* = 1877). These studies provide the basis for further linking gene expression with resting-state brain functional phenotypes, such as ReHo. Allen Human Brain Atlas (AHBA) provides a new approach for linking gene expression to neuroimaging phenotypes without stringent requirements for sample size (Fornito et al., 2019). By projecting gene expression data from postmortem human brains and neuroimaging data from living human brains to the same standard space, spatial correlation analysis between gene expression and neuroimaging measurement across brain regions or tissue samples can identify genes associated with neuroimaging phenotypes (Fornito et al., 2019). With this approach, several studies have provided new molecular insights into the neuroimaging phenotypes of both healthy and diseased brains (Hawrylycz et al., 2015; Rittman et al., 2016; Romme et al., 2017; Romero-Garcia et al., 2018; Morgan et al., 2019). However, none of these studies have investigated the association between gene expression and ReHo.

In humans, there are more than 20,000 genes, thousands of which have unknown functions. Gene-wise spatial correlations with neuroimaging phenotypes may face challenges not only in correcting multiple comparisons but also in interpreting significant genes with unknown functions. Weighted gene coexpression network analysis (WGCNA) has been proposed to cluster more than 20,000 genes into several dozen gene modules based on the similarity of their spatial expression patterns (Langfelder and Horvath, 2008). Using a module eigengene (ME) to represent the gene expression profile of each module, one can identify gene modules associated with neuroimaging phenotypes by analyzing spatial correlations between ME expression and neuroimaging phenotypes across brain regions, through which the numbers of comparisons are greatly reduced. Regarding the interpretability of significant gene modules, the functions of each module (generally consisting of hundreds of genes) can be investigated through a variety of enrichment analyses. For example, with the RNA-seq data of various types of purified human neocortical cells, one can identify the cell type in which a gene module shows specific expression (Xu et al., 2014). One can also investigate the enrichment of a certain module for biological processes, molecular functions and cellular components (Ashburner et al., 2000). Moreover, one can identify genes shared by neuroimaging phenotypes and brain disorders through enrichment analyses of phenotype-related genes for GWAS results of neuropsychiatric diseases (Gandal et al., 2018). These analyses may provide valuable insight into the molecular mechanisms underlying the neuroimaging abnormalities observed in these disorders.

**FIGURE 1:**
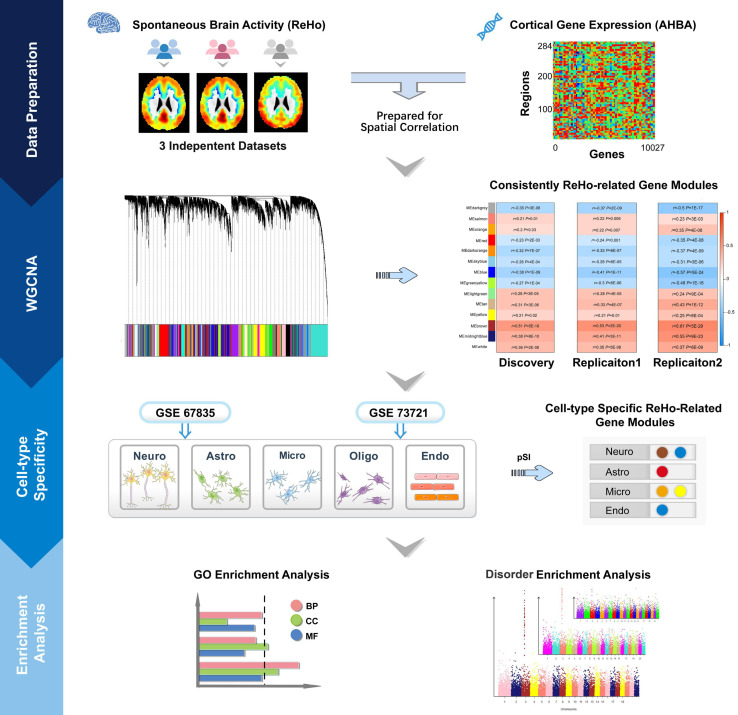
Schematic summary of the study design. This study comprised data preparation, WGCNA, cell-type-specific analysis and enrichment analysis. During data preparation, three mean ReHo maps were obtained from three independent datasets, and a cortical gene transcription matrix was constructed from AHBA. Both ReHo maps and the transcription matrix were assigned to neocortical regions from the HCP Atlas. WGCNA was used to cluster individual genes into gene modules, and spatial correlation was then performed between the ReHo maps and the gene expression profile of each gene module. The significantly correlated gene modules common to the three datasets were considered ReHo-related gene modules. Cell-type-specific analysis was performed for each ReHo-related gene module based on the transcriptomic profiles of neurons, astrocytes, oligodendrocytes, microglia, and endothelial cells from the GSE73721 and GSE67835 series, and the modules that were consistently related to a specific type of neocortical cells in both series were included in the following enrichment analysis. Finally, the identified cell-type-specific modules were annotated by GO and brain disorder enrichment analysis to identify the functions of these modules and to establish their relationships with brain disorders. GO, Gene Ontology; GSE, gene series expression; HCP, Human Connectome Project; ReHo, regional homogeneity; WGCNA, weighted gene coexpression network analysis.

In this exploratory study, we aimed to clarify the following questions: (a) which gene modules show consistent spatial correlations between gene expression and ReHo across neocortical regions; (b) which ReHo-related gene modules are specifically enriched in particular types of cells in the cerebral cortex; (c) what biological processes, molecular functions and cellular components are associated with these cell-type-specific modules; and (d) which cell-type-specific ReHo-related gene modules are related to common brain disorders. A schematic summary of the study design is shown in Figure 1.

## Materials and Methods

### Participants

After excluding participants with any neuropsychiatric illnesses, contraindications for MRI examination, or imaging artifacts, 1101 right-handed healthy young Chinese Han participants (509 males and 592 females; mean age: 24 years, ranging from 18 to 30 years) were recruited from Tianjin Medical University General Hospital (discovery sample: *n* = 409, 161 males and 248 females; mean age: 24 years, ranging from 18 to 30 years) and Cancer Hospital (replication sample 1: *n* = 692, 348 males and 344 females; mean age: 24 years, ranging from 18 to 30 years). The study protocol was approved by the Medical Research Ethics Committee of Tianjin Medical University, and written informed consent was obtained from each participant. To generalize our findings to participants of other ethnicities, the data of 600 healthy young non-Chinese adults (replication sample 2: 297 males and 303 females; mean age: 29 years, ranging from 22 to 36 years) were obtained from the Human Connectome Project (HCP) (Van Essen et al., 2013) (Supplementary Table 1).

### MRI Data Acquisition

In Tianjin Medical University General Hospital and Cancer Hospital, MRI data were acquired by using Discovery MR750 3.0-Tesla MR scanners (General Electric, Milwaukee, WI, United States) with the same parameters. Tight but comfortable foam padding was used to minimize head motion, and earplugs were used to reduce scanner noise. The rs-fMRI data were acquired using the Gradient-Echo Single-Shot Echo-Planar Imaging (GRE-SS-EPI) sequence with the following parameters: repetition time (TR)/echo time (TE) = 2000/30 ms; field of view (FOV) = 220 mm × 220 mm; matrix = 64 × 64; flip angle (FA) = 90°; slice thickness = 3 mm; gap = 1 mm; 36 interleaved transverse slices; and 180 volumes. All subjects were instructed to keep their eyes closed, relax, move as little as possible, think of nothing in particular, and stay awake during fMRI scans. Sagittal 3D T1-weighted images were acquired by brain volume sequence (TR/TE = 8.16/3.18 ms; inversion time = 450 ms; FA = 12°; FOV = 256 mm × 256 mm; matrix = 256 × 256; slice thickness = 1 mm, no gap; 188 slices). The HCP MRI data were collected by a customized 3.0-Tesla MR scanner. The rs-fMRI data were acquired by the GRE-SS-EPI sequence (TR/TE = 720/33.1 ms; FOV = 208 mm × 180 mm; matrix = 104 × 90; FA = 52°; slice thickness = 2 mm, no gap; 72 transverse slices; and 1200 volumes), and the 3D-T1-weighted images were acquired by the magnetization prepared rapid acquisition gradient echo sequence (TR/TE = 2400/2.14 ms; inversion time = 1000 ms; FA = 8°; FOV = 224 mm × 224 mm; matrix = 320 × 320; slice thickness = 0.7 mm, no gap; 260 sagittal slices).

### MRI Data Preprocessing

All fMRI data were preprocessed with the same procedures. The first 5 volumes were discarded to allow signals to reach equilibrium and to ensure that the participants had adapted to scanning noise. The remaining volumes were corrected for intra-volume temporal differences using sinc-interpolation. Inter-volume head motion was then corrected using rigid-body transformations. After removing non-brain tissues from functional and structural images, functional images were co-registered to corresponding structural images using the boundary-based registration method. Structural images were spatially normalized to the Montreal Neurological Institute (MNI) space, and functional images were normalized to the MNI space using the transformation parameters derived from structural image normalization and were resampled to 3-mm isotropic voxels. Several sources of variance were regressed out from the functional images, including the frame-wise displacement (volume-to-volume changes in head position), linear drift, Friston’s 24 head motion parameter, and signals from the white matter and ventricles.

### Individual-Level zReHo Calculation for Each Voxel

For each gray matter voxel of each subject, ReHo was defined as the KCC of spontaneous brain activity between this voxel and its nearest 26 neighboring voxels (Zang et al., 2004). To improve the normality and reliability of this measure across subjects, a zReHo values was calculated for each voxel by subtracting the mean ReHo values and dividing it by the standard deviation of all gray matter voxels (Zuo et al., 2013). The resulting zReHo map of each subject was spatially smoothed with an 8 mm × 8 mm × 8 mm full width at half-maximum Gaussian kernel.

### Group-Level zReHo Calculation for Each Brain Region

For each of the three groups (one discovery group and two replication groups), the corresponding zReHo map was calculated by voxel-wised one-sample *t*-test using SPM8^1^ within the gray matter mask, and the mean zReHo maps of each of the three groups were also calculated and are provided in Supplementary Figure 1. Brain regions of interest were defined based on the HCP Atlas (HCP’s multi-modal parcelation version 1.0, HCP_MMP1.0), which divides the human cerebral cortex into 360 non-overlapping regions (Glasser et al., 2016). In each group, we calculated the mean zReHo for each region by averaging the zReHo values of all voxels of that region in the corresponding zReHo map. Thus, we obtained the mean zReHo values for each of the 360 cerebral cortical regions in each of the three groups.

### Gene Expression Data Processing

The AHBA provided six donated postmortem brains with 3702 densely sampled expression data of more than 20,000 genes detected by 58,692 probes. To avoid biases from gross gene expression dissimilarities between brain regions, we excluded 1998 samples from subcortical nuclei, brainstem and cerebellum, and kept 1704 cerebral cortical samples. We followed a standardized pipeline proposed for AHBA data processing to link gene expression and neuroimaging phenotypes (Arnatkevic Iute et al., 2019). Specifically, the Re-Annotator toolkit v1.0 was used to update probe-to-gene annotations with the up-to-date gene symbol ID and name (Arloth et al., 2015), and the resulting 45,821 probes (20,232 genes) were used for further processing. After intensity-based filtering, 31,977 probes (15,746 genes) with expression values exceeding the background in at least 50% of the samples were preserved. After probe selection, 10,027 probes showing the highest correlation with RNA-seq gene expression were preserved to represent the expression of the corresponding 10,027 genes. The 10,027 genes exhibited relatively high reproducible expression patterns across brain structures between donors, indicating that they are suitable for investigating correlations between expression data from postmortem donors and neuroimaging data from living humans. For each AHBA brain, each tissue sample was assigned to the nearest neocortical region of the HCP Atlas with a recommended distance threshold of less than 2 mm (Arnatkevic Iute et al., 2019). In addition, samples that were assigned to a hemisphere that differed from the annotations provided with their MNI coordinates were excluded. After sample assignment, 820 out of 1704 cortical tissue samples were matched to brain regions in the HCP Atlas (Supplementary Table 2). For each brain region with more than one tissue sample, the mean gene expression values of these samples was defined as the expression values of the gene in that region. Due to the lack of any matched tissue samples, 76 regions were excluded from further analysis. Thus, we obtained the expression values of each gene in each of the 284 regions. Scaled robust sigmoid normalization was applied to remove donor-specific variability in gene expression (Fulcher et al., 2013). Finally, a gene transcription matrix of 284 × 10,027 regions × genes was constructed. The code for implementing the AHBA gene expression data processing steps can be downloaded from https://github.com/BMHLab/AHBAprocessing.

### Calculation of Gene Modules

Based on the gene transcription matrix of 284 × 10,027 regions × genes, WGCNA was used to identify gene modules and a gene module was defined as a set of genes with similar expression profile across brain regions (*n* = 284). For each brain region (*n* = 284), we performed principal component analysis (PCA) of the expression profiles of genes of each module (*n* = 30) and defined the first component as the module eigengene (ME) to represent the gene expression profile of this module. Specifically, according to the criterion of approximate scale-free topology (fit index = 0.9), a soft thresholding power of 7 was chosen to transform the correlation matrix into an adjacency matrix. Then, the topological similarity of gene expression was calculated using the adjacency matrix. A hierarchical clustering algorithm was used to generate a hierarchical clustering tree (dendrogram) of genes. Gene modules with similar expression profiles were obtained with dynamic tree-cutting with the following parameters: a minimum module size of 50 genes; a deep split of 2; and a height threshold of 0.1. The ME, representing the expression profile of a module, was defined as the first principal component of each module. Finally, we obtained ME values of each gene module (*n* = 30) of each brain region (*n* = 284) from AHBA data.

### Identification of ReHo-Related Gene Modules

Based on the ME values of the 30 modules × 284 brain regions and zReHo values of the same 284 brain regions, spatial correlation was performed between ME and zReHo across these brain regions for each of the 30 modules, respectively. Since brain regions have different ME values of the same gene module, we have considered the transcriptional variation of each gene module between brain regions. However, the spatial correlation was performed between gene expression and ReHo data derived from different individuals, only genes with similar expression profiles across individuals can be identified by this approach. Because the ME of all 30 gene modules deviated from a normal distribution (Supplementary Table 3), the ReHo-related gene modules were identified by the spatial Spearman correlation between the ME of each module and zReHo across the 284 neocortical regions. The same Bonferroni method was used to correct for multiple comparisons (*P*_c_ < 0.05, equal to an uncorrected *P* < 0.05/30 = 0.0017) in both the discovery and the two replication samples. The criterion for replication was defined as statistically significant in both the discovery and the two replication samples.

### Cell-Type-Specific Analysis

We performed cell-type-specific analysis for each ReHo-related gene module. The GSE73721 and GSE67835 series from normal adult human neocortices include the transcriptomic profiles of purified neurons, astrocytes, oligodendrocytes, microglia, and endothelial cells (Darmanis et al., 2015; Zhang et al., 2016). RNA-seq data of the two series were separately processed using the following steps: (a) sequencing files downloaded from the Sequence Read Archive (SRA) database^2^ were converted to Fastq files; (b) Prinseq v0.20.4 (Schmieder and Edwards, 2011), FASTQC v0.11.8^3^, Trim Galore v0.6.4_dev^4^ and Cutadapt v2.7 (Martin, 2011) were used to trim and filter reads and to identify and remove adaptors; (c) reads were aligned to the hg38 genome with STAR v2.7.3a (Dobin et al., 2013) and converted to counts using HTSeq v0.11.2 (Anders et al., 2015); (d) reads per kilobase per million (RPKM) values were calculated following the EdgeR pipeline v3.30.3 (Robinson et al., 2010; McCarthy et al., 2012); and (e) the averaged RPKM values of each neocortical cell type were used in the specificity index (SI) analysis to determine the specific neocortical cell type for which the Reho-related modules were enriched using pSI v1.1^5^. Specifically, RPKM values from one neocortical cell type were compared to those of the other cell types across genes. For each comparison between cell types, the genes were ranked from the highest to the lowest fold changes. The SI for each gene was calculated as the average rank across all comparisons. A *P*-values was assigned to each SI values via permutation testing, resulting in a pSI values, representing how likely it was that a gene was specifically expressed in a given cell type relative to other cortical cells. A pSI threshold of 0.05 was used to generate a cell-type-enriched gene list. Fisher’s exact test was used to evaluate the significance of the overlap between the candidate gene list in the module and the cell-type-specific genes as the background list. The Bonferroni method was used to correct for multiple comparisons (5 cell types and 14 ReHo-related modules) (*P_c_* < 0.05, uncorrected *P* < 0.05/5/14 = 7.14 × 10^–4^).

### Gene Ontology Enrichment Analysis

To characterize the possible biological processes, molecular functions and cellular components of each ReHo-related cell-type-specific gene module, GO enrichment analysis was performed with WebGestalt v2019^6^ using Fisher’s exact test with FDR correction (*q*_c_ < 0.05) (Liao et al., 2019).

### Enrichment Analysis for Common Brain Disorders

Associations between ReHo-related cell-type-specific gene modules and common brain disorders were identified with MAGMA v1.07b, a software for gene analysis and generalized gene-set analysis of GWAS data (de Leeuw et al., 2015). The single nucleotide polymorphism (SNP) *P*-values of the GWAS summary statistics of common brain disorders (AD, PD, EP, Stroke, MS, BD, MDD, SCZ, ADHD and ASD) were obtained from previous studies (Pankratz et al., 2012; Beecham et al., 2013; Ripke et al., 2013; Autism Spectrum Disorders Working Group of The Psychiatric Genomics Consortium, 2017; International League Against Epilepsy Consortium on Complex Epilepsies, 2018; Malik et al., 2018; Martin et al., 2018; Pardinas et al., 2018; International Multiple Sclerosis Genetics Consortium, 2019; Jansen et al., 2019; Kunkle et al., 2019; Stahl et al., 2019; Supplementary Table 4). The European panel of the 1000 Genomes phase 3 data were used as reference dataset to account for linkage disequilibrium (LD) between SNPs. The SNP locations in the data were determined in reference to human genome build 37 or 36. MAGMA-based enrichment basically consists of two steps: gene analysis and gene-set analysis.

The gene analysis followed a multiple linear principal component regression model to project the SNP *P* values matrix for a gene onto its principal components, which were used as predictors for the brain disorders in the linear regression model to calculate a gene *P* values. The gene *P* values was then converted to *Z* values to improve normality, which reflects the correlation strength of each gene with each brain disorder. In addition, a gene-gene correlation matrix was calculated to account for the dependency between genes in the following gene-set analysis.

In the gene-set analysis, each gene set consisted of the genes of each ReHo-related cell-type-specific module and represented as binary indicator variables, coded 1 for disease-related genes in that gene module and 0 otherwise. General linear regression analysis was performed at the gene level to test whether genes in the module were more strongly associated with brain diseases than genes outside the module while correcting for gene size, gene density and the minor allele count. Then, the gene-set *P* values were obtained and subjected to Bonferroni correction (the number of comparisons corresponding to the ReHo-related cell-type-specific gene modules) to assess the enrichment of the GWAS signal for each gene module. Moreover, the gene *P* values of the significant enrichment module were also subjected to Bonferroni correction (accounting for the total number of genes in the module) to identify the significant genes within each module for brain disorders.

## Results

### ReHo-Related Gene Modules

**FIGURE 2:**
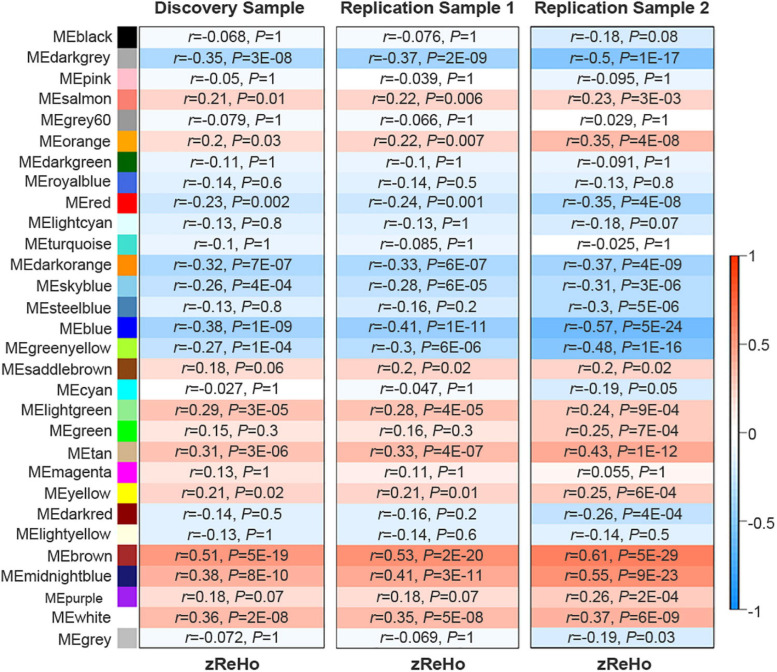
Identification of ReHo-related gene modules. The original 30 modules in the discovery sample and the two replication samples. Color bar denotes correlation coefficients between ME and zReHo. Warm color represents positive correlations and cold color represents negative correlations with correlation coefficients and Bonferroni-corrected *P* values listed for each module. ME, module eigengene; zReHo, z transformed regional homogeneity.

Based on the similarity of gene expression across brain regions, WGCNA divided the 10,027 genes into 30 non-overlapping gene modules (Supplementary Figure 2). Spatial correlations identified 14 significant ReHo-related gene modules across neocortical samples in the discovery sample of 409 Chinese subjects. In a replication sample of 692 Chinese subjects, 15 significant ReHo-related gene modules were identified across the neocortical samples, and 14 of these modules were identical to those in the discovery cohort. Furthermore, in another replication sample of 600 non-Chinese people, 14 out of the 20 gene modules were repeated. Thus, the 14 gene modules common to the three cohorts were considered as candidate ReHo-related gene modules (Figure 2 and Supplementary Table 5).

### Cell-Type Specificity of ReHo-Related Gene Modules

**FIGURE 3:**
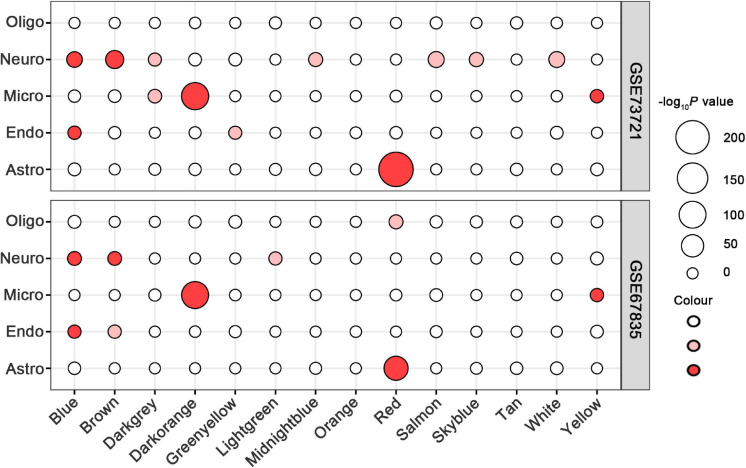
Cell-type-specific analyses for ReHo-related modules in the GSE73721 and GSE67835 series. The size of a given circle corresponds to the cell-type-specific enrichment -log_10_ Bonferroni-corrected *P*-values for each module. Solid red indicates significant enrichment in both series, faint red indicates significant enrichment in only one series, and white indicates non-significant in neither series. GSE, gene series expression; ReHo, regional homogeneity.

**FIGURE 4:**
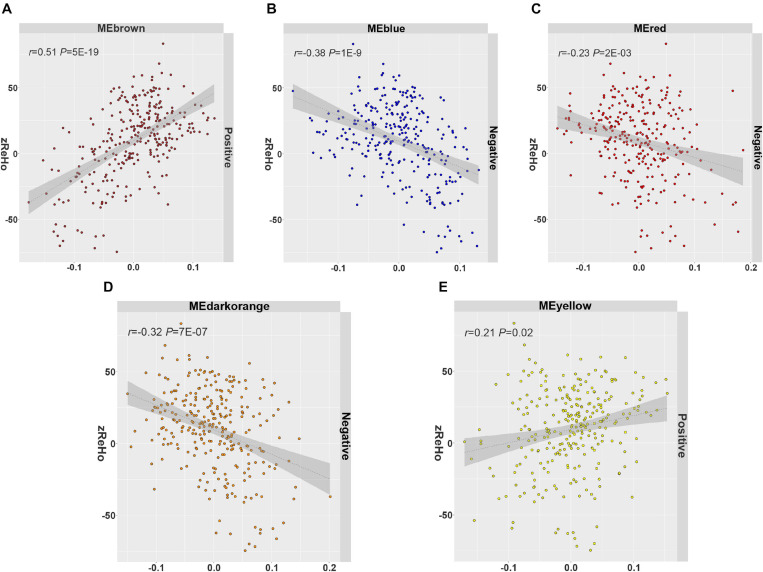
The spatial correlation between the ME and zReHo of each cell-type-specific module with the correlation coefficient and Bonferroni-corrected *P* values. **(A)** Brown module, **(B)** blue module, **(C)** red module, **(D)** dark orange module, **(E)** yellow module in the discovery sample. ME, module eigengene; zReHo, z transformed regional homogeneity.

In all 14 modules, only five ReHo-related gene modules showed consistent enrichment for a specific type of neocortical cells in both the GSE73721 and GSE67835 series, and these modules were deemed cell-type-specific modules. Specifically, the genes of the blue module were enriched in neurons and endothelial cells (*P*_c_ = 6.31 × 10^–9^ in GSE73721, and *P_c_* = 7.73 × 10^–4^ in GSE67835 for neuron; *P*_c_ = 1.18 × 10^–2^ in GSE73721, and *P_c_* = 1.87 × 10^–2^ in GSE67835 for endothelial cell); the genes of the brown module were enriched in neurons (*P_c_* = 2.17 × 10^–20^ in GSE73721, and *P_c_* = 1.35 × 10^–3^ in GSE67835); the genes of the red module were enriched in astrocytes (*P_c_* = 6.02 × 10^–219^ in GSE73721, *P_c_* = 1.29 × 10^–68^ in GSE67835); and the genes of the dark orange and yellow modules were enriched in microglia (*P_c_* = 8.44 × 10^–105^ in GSE73721, *P_c_* = 5.43 × 10^–102^ in GSE67835 for the dark orange module; *P_c_* = 8.22 × 10^–3^ in GSE73721, *P_c_* = 1.17 × 10^–2^ in GSE67835 for the yellow module) (Figure 3 and Supplementary Table 6). However, none of these modules showed significant specific expression in oligodendrocytes in either series. The ME values of the brown and yellow modules showed a positive correlation with zReHo values across neocortical regions (*r* = 0.51, *P_c_* = 5 × 10^–19^ for brown module; *r* = 0.21, *P_c_* = 0.02 for yellow module); however, the ME values of the blue, red and dark orange modules showed negative correlations with zReho values across neocortical regions (*r* = −0.38, *P_c_* = 1 × 10^–9^ for blue module; *r* = −0.23, *P_c_* = 2 × 10^–3^ for red module; *r* = −0.32, *P_c_* = 7 × 10^–7^ for dark orange module) in both the discovery sample (Figure 4) and the two replication samples (Supplementary Figures 3, 4).

Among the top hit genes obtained from GWAS of resting-state fMRI phenotypes, *INPP5A* in blue module that has been associated with resting-state activity of the prefrontal cortex in the UK Biobank paper (Elliott et al., 2018) was also associated with zReHo, a measure of resting-state brain activity.

### GO Enrichment for Cell-Type-Specific ReHo-Related Gene Modules

**FIGURE 5:**
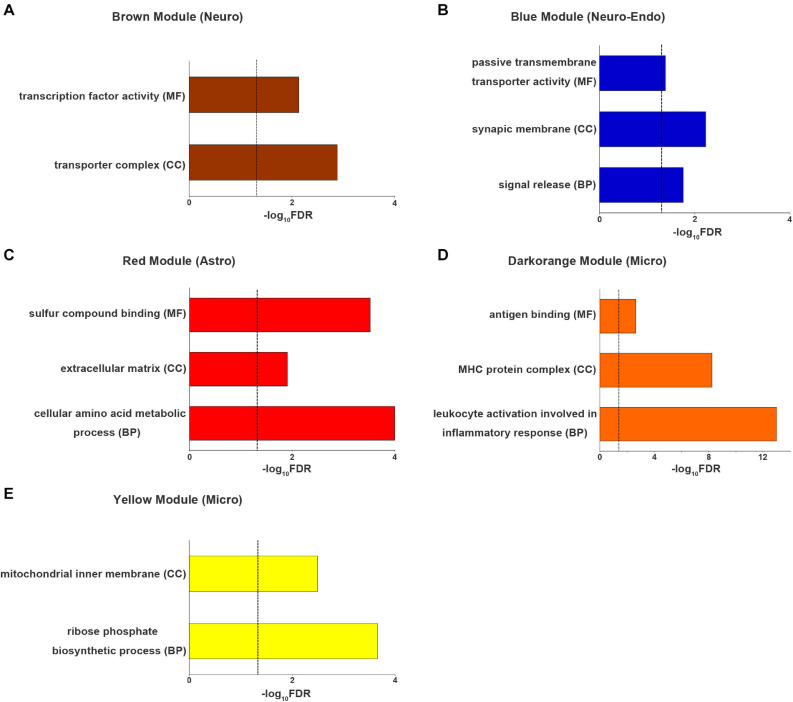
The top enriched Gene Ontology terms for each cell-type-specific module. **(A)** neuron (brown) module, **(B)** neuron-endo (blue) module, **(C)** astrocyte (red) module and **(D,E)** microglia (darkorange and yellow) modules. The dashed line indicates the FDR-corrected threshold. Astro, astrocytoma; BP, biological process; CC, cellular component; Endo, endothelium; Micro, microglia; FDR, the corrected *P* values with the false discovery rate method; MF, molecular function; Neuro, neuron; Oligo, oligodendrocyte.

The genes of brown module (neuron-endothelial module) were significantly enriched for transporter complexes (*q_c_* = 1.3 × 10^–3^) and transcription factor activity (*q*_c_ = 7.3 × 10^–3^) (Figure 5A). The genes of blue module (neuron module) were associated with signal release (*q*_c_ = 1.8 × 10^–2^), the synaptic membrane (*q_c_* = 5.9 × 10^–3^) and passive transmembrane transporter activity (*q*_c_ = 4.1 × 10^–2^) (Figure 5B). The genes of red module (astrocyte module) were enriched for cellular amino acid metabolic processes (*q*_c_ = 1 × 10^–4^), the extracellular matrix (*q*_c_ = 1.2 × 10^–2^) and sulfur compound binding (*q*_c_ = 3 × 10^–4^) (Figure 5C). The genes of dark orange module (microglial module) were associated with leukocyte activation involved in inflammatory response (*q*_c_ = 1.0 × 10^–13^), major histocompatibility complex (MHC) protein complexes (*q*_c_ = 5.6 × 10^–9^) and antigen binding (*q*_c_ = 2.3 × 10^–3^) (Figure 5D). The genes of yellow module (microglial module) were associated with ribose phosphate biosynthetic processes (*q*_c_ = 2.2 × 10^–4^) and the mitochondrial inner membrane (*q*_c_ = 3.2 × 10^–3^) (Figure 5E). Other significantly enriched GO terms of the five cell-type-specific modules are listed in Supplementary Table 7.

### Brain Disorder Enrichment for Cell-Type-Specific ReHo-Related Gene Modules

**FIGURE 6:**
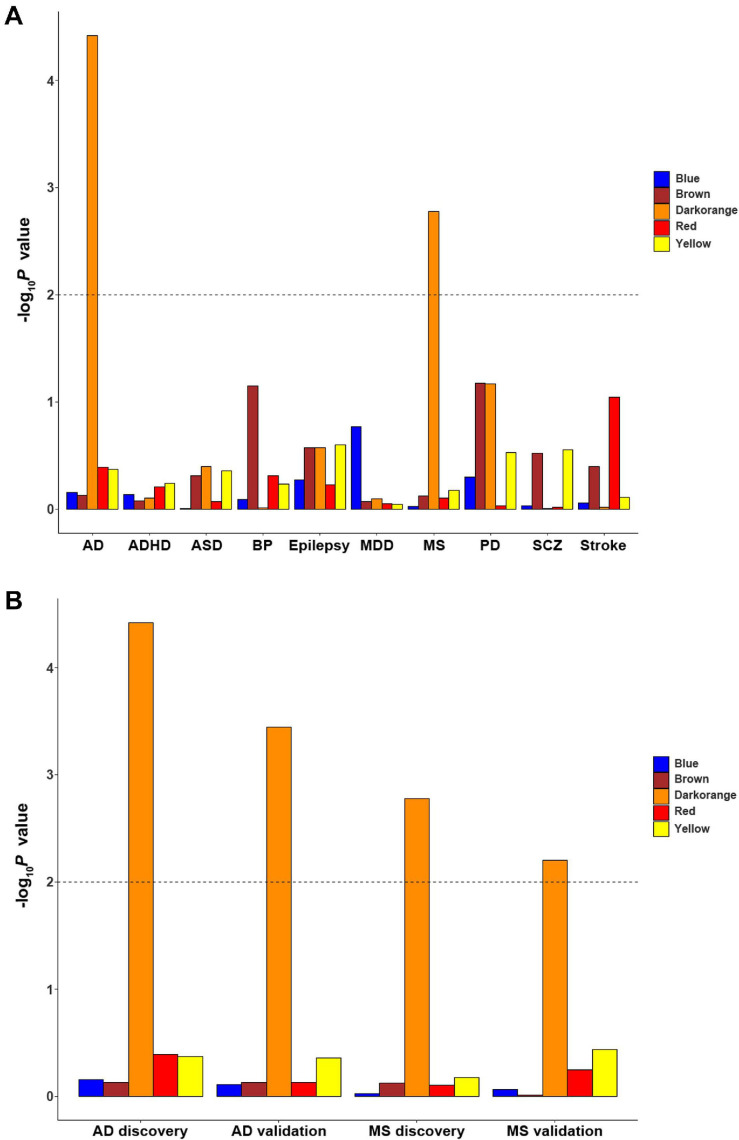
The enrichment of cell-type-specific modules in ten common brain disorders. **(A)** The significant finding is that the microglial module is significantly enriched for MS and AD. **(B)** The discovery and replication enrichment for MS and AD. The dashed line indicates the Bonferroni-corrected threshold for 5 cell-type-specific modules. AD, Alzheimer’s disease; ADHD, attention deficit hyperactivity disorder; ASD, autism spectrum disorders; BP, bipolar disorder; EP, epilepsy; MDD, major depressive disorder; MS, multiple sclerosis; SCZ, schizophrenia; PD, Parkinson’s disease.

Since ReHo abnormalities have been reported in brain disorders, enrichment was applied to investigate whether these cell-type-specific ReHo-related gene modules are associated with genetic susceptibility for common brain disorders. Among the 10 common brain disorders considered herein, only the microglial module was significantly enriched for multiple sclerosis and Alzheimer’s disease (MAGMA gene-set *P*_c_ values of 8.26 × 10^–3^ and 1.88 × 10^–4^, respectively, accounting for the 5 cell-type-specific modules) (Figure 6A and Supplementary Table 8). The other two GWAS summary statistics of multiple sclerosis and Alzheimer’s disease with different sample sizes from previous analysis were further tested to validate our findings. After Bonferroni correction for 5 cell-type-specific modules, the microglial module again exhibited significant associations with multiple sclerosis and Alzheimer’s disease (MAGMA gene-set *P*_c_ values of 3.12 × 10^–2^ and 1.78 × 10^–3^, respectively) (Figure 6B and Supplementary Table 8).

The comparison of MAGMA gene *P* values against a Bonferroni-corrected threshold of 0.05/94 = 5.32 × 10^–4^ in the microglial module revealed that the significant genes associated with MS were interferon gamma-inducible protein 30 (*IFI30*), regulator of G-protein signaling 1 (*RGS1*), and cluster of differentiation 86 (*CD86*) in both MS GWAS summaries. Comparison against a threshold of 0.05/101 = 4.95 × 10^–4^ in the microglial module revealed that the significant genes associated with AD were membrane spanning 4-domains A4A (*MS4A4A*), human leukocyte antigen-DR alpha (*HLA-DRA*), triggering receptor expressed on myeloid cells 2 (*TREM2*), human leukocyte antigen-DR beta 5 (*HLA-DRB5*) and cluster of differentiation 33 (*CD33*) in both AD GWAS summaries (Supplementary Table 9).

## Discussion

This study identified five cell-type-specific gene modules associated with ReHo. The neuron-endothelial module was enriched for transporter complexes, the neuron module for the synaptic membrane, the astrocyte module for amino acid metabolism, and the microglial modules for leukocyte activation and ribose phosphate anabolism, indicating that neurons, astrocytes, microglia and endothelial cells are all associated with the ReHo of spontaneous brain activity. We also found that the ReHo-related microglial module was enriched for MS and AD, indicating that the molecular pathology of microglial cells is a possible mechanism underlying ReHo abnormalities in both diseases.

Regional homogeneity is a measure of the local synchronization of BOLD signals among neighboring voxels (Zang et al., 2004), and the BOLD signal is a reflection of neurovascular coupling linking neural firing to cerebral blood flow changes to accommodate changing energy demands during brain activity (Metea and Newman, 2006). Neurovascular coupling depends on the structure and function of the neurovascular unit consisting of neurons, astrocytes and vessel endothelial cells. The associations between ReHo and gene expression in the neuron, astrocyte and neuron-endothelial modules are consistent with the neurovascular coupling theory of BOLD signals. In this study, we identified two sets of genes significantly expressed in neurons that exert different modulatory effects on the ReHo of spontaneous brain activity. The higher expression of the brown module related to transporter complexes and ligand-activated transcription factor activity corresponds to higher ReHo; however, the higher expression of the blue module related to the formation of the synaptic membrane, signal release, and passive transmembrane transporter activity corresponds to lower ReHo. These findings indicate that the ReHo of spontaneous brain activity may be modulated by neuron-related genes with different functions. In addition, the endothelial component in the blue module may also account for this difference. Astrocytes coordinate information exchange between neurons and vessels and act as a hub for neurovascular coupling (Filosa et al., 2016). We found that an astrocyte module enriched for extracellular matrix formation, sulfur compound binding, and amino acid metabolic processes was associated with ReHo, indicating that it is not only gene expression in neurons but also expression in astrocytes that may play an important role in modulating the ReHo of spontaneous brain activity.

A novel finding of this study was the association between microglia-related genes and the ReHo of spontaneous brain activity. Microglia in the central nervous system are responsible for regulating the immune response involving antigen presentation, debris phagocytosis and cytokine production (Sasaki, 2017). The significant enrichment of the dark orange module for the MHC, antigen binding and leukocyte activation was consistent with the immune inflammatory role of microglia. The yellow module was also specifically expressed in microglia but with different modulatory effects in ReHo compared with the dark orange module. The enrichment of ribose phosphate biosynthetic processes and the mitochondrial inner membrane observed for the yellow module may indicate that genes in this module may participate in adenosine triphosphate synthesis in mitochondria for energy supplementation to microglia. Interindividual and interregional variations in microglia-related gene expression will influence the function of microglia, resulting in individual and regional differences in the production of cytokines and the removal of free radicals (Kaur et al., 2017). Both cytokines and free radicals influence components of the neurovascular unit and, thus, ReHo.

In the enrichment analysis for common brain disorders, one significant result was the enrichment of the microglial module for multiple sclerosis, which was consistent with previous findings of microglial abnormalities in multiple sclerosis (Voet et al., 2019). In early active multiple sclerosis, microglia predominantly display a proinflammatory phenotype and express molecules involved in oxidative injury, phagocytosis, T cell stimulation, antigen presentation, and iron metabolism (Zrzavy et al., 2017). In addition to the active lesions, a similar microglial activation pattern was observed for the gene expression of different surface markers at the site of lesion expansion in chronic MS lesions (Frischer et al., 2009; Zrzavy et al., 2017). Furthermore, microglial modules (clusters of activated microglia) are observed in the white matter in the shadow of plaques in MS patients, which are deemed pre-active MS lesions (Ramaglia et al., 2012; Singh et al., 2013). PET using 18-kDa translocator protein (TSPO)-binding radioligands can detect MS-related pathology at the molecular level *in vivo*. Increased TSPO binding is observed in scattered areas related to demyelinating lesions in relapsing-remitting MS patients, which further supports neuroinflammation and neuronal injury involving activated microglia for MS (Airas et al., 2015). In the dark orange microglial module, three genes associated with MS were identified in both GWAS datasets. *IFI30* plays a crucial role in MHC class II-restricted antigen processing, and its expression is greatly enhanced on microglia in active demyelinating lesions of multiple sclerosis (Maric et al., 2001; Satoh et al., 2008). Previous studies have shown the expression of *CD86* in microglia within brain lesions in multiple sclerosis (Windhagen et al., 1995). Moreover, the expression of *CD86* is influenced by local variants correlated with disease susceptibility in the pathogenesis of multiple sclerosis (Smets et al., 2018). Regulators of G-protein signaling (RGS) proteins, especially *RGS1*, play a key role in the negative regulation of G-protein-coupled receptor signaling (Neves et al., 2002). There is a growing body of literature that suggests that *RGS1* is expressed in microglia (Atwood et al., 2011). *RGS1* presents a close relationship with neuroinflammation by responding to diverse chemokines (Balashov et al., 1999) and was recently designated as an MS susceptibility locus by the International Multiple Sclerosis Genetics Consortium (International Multiple Sclerosis Genetics Consortium, 2010).

Our enrichment analysis also provided further evidence that the dark orange microglial module was associated with genetic susceptibility for AD (Hansen et al., 2018). In addition to the characteristic histopathological findings of extracellular amyloid-β (Aβ) plaques and intracellular neurofibrillary tangles (NFTs), the cumulative evidence supports microglia-mediated neuroinflammation as a major contributor to the neurodegenerative processes and cognitive deficits observed in AD (Heneka et al., 2015). Histopathological studies showing activated microglia surrounding Aβ plaques and NFTs suggest a relationship between neuroinflammation and AD pathology (Rozemuller et al., 1992; Sheffield et al., 2000). Neuroinflammation in AD is recognized as a ‘double-edged sword’ in which microglia exhibit both neuroprotective and neurotoxic effects (Hansen et al., 2018). In the initial phase of AD, moderate activation of microglia exerts an anti-inflammatory function, promoting Aβ clearance and eliminating reactive oxygen and nitrogen species (ROS/RNS) (Ahmad et al., 2019). With the progression of AD, overactivated microglia secrete proinflammatory mediators and upregulate oxidative stress, which may exacerbate Aβ deposition and NFT formation, ultimately leading to progressive neuronal and synaptic damage (Ahmad et al., 2019). By using TSPO to reflect microglial activation, PET imaging allows the visualization and quantification of AD-related neuroinflammatory changes *in vivo* (Varley et al., 2015). Despite conflicting results, it is generally accepted that increased TSPO radioligand binding occurs across brain regions and is correlated with tau aggregation and amyloid deposition (Dani et al., 2018; Edison et al., 2018), which further supports the microglia-mediated neuroinflammation hypothesis for AD. In the dark orange microglial module, a total of five genes associated with AD were identified in both GWAS datasets, most of which were established AD-susceptibility genes (Naj et al., 2011; Guerreiro et al., 2013; Lambert et al., 2013). *MS4A4A* is a member of the *MS4A* gene family and is highly expressed on the plasma membrane in microglia in the brain; its functions are still poorly understood, but possible roles in protein trafficking and clathrin-dependent endocytosis have been indicated (Cruse et al., 2015). Previous evidence suggested that the expression of *MS4A4A* was increased in the brain tissue of autopsied AD patients (Allen et al., 2012). *TREM2* lies in a cluster of *TREM* family genes and is uniquely expressed on the surface of microglia in the brain (Gao et al., 2017). Mounting evidence indicates that *TREM2* may modulate AD-related neuropathology by suppressing the inflammatory response, increasing Aβ phagocytosis, ameliorating tau pathology and promoting neuronal survival, thus contributing to neuroimmune homeostasis (Yeh et al., 2017). The *HLA-DRA* and *HLA-DRB5* genes are members of MHC class II, a highly polymorphic region involved in the immune response and histocompatibility; both of these genes are predominantly expressed by microglia in the brain and might play a role in susceptibility to AD (Zhang et al., 2015; Villegas-Llerena et al., 2016; Yokoyama et al., 2016). *CD33* belongs to the sialic acid-binding immunoglobulin-like lectin (Siglec) family and is a myeloid cell receptor that is mainly expressed by microglia in the brain (Jiang et al., 2014). The expression of *CD33* is elevated in AD patients’ brains, where it is thought to impair Aβ clearance via immunoreactive microglia (Bradshaw et al., 2013; Griciuc et al., 2013).

There are several limitations to our study. First, there are currently no AD and MS patients for whom both gene expression data and ReHo data are available, which prevents us from establishing a causal link between the expression of microglia-related genes and ReHo abnormalities in these two brain disorders. Further replication in patients with MS and AD may provide us with a more complete understanding of the effects of the ReHo-related microglial module on both disorders. Second, for each gray matter voxel of each subject, ReHo is a measure of the temporal coherence of the fluctuation of the mean BOLD signals of this voxel with its nearest 26 neighboring voxels. For each time point, the hemodynamic signal of each voxel is the mean signal of this voxel during a period of TR. Although we cannot exclude the effect of the overlapping signals on ReHo, the similar results derived from fMRI data with different scan parameters (discovery sample and replication sample 1: TR = 2000 ms, voxel size = 3.4 × 3.4 × 3.0 mm with 1 mm gap; replication sample 2: TR = 720 ms, voxel size = 2.0 × 2.0 × 2.0 mm without gap) indicate a relatively small effect of the overlapping signals on ReHo. Third, the zReHo values of neocortical regions used for spatial correlation analysis were derived from the group-level zReHo map, which may loss the information of individual variation. The best way to establish the connection between ReHo and gene expression in a given brain region is to observe their correlation in a large number of individuals with both gene expression data of this region and brain fMRI data to calculate ReHo of this region. However, no such large-scale data are available so far, even for a single brain region. An alternative but suboptimal way is to investigate the spatial correlation between ReHo and gene expression across brain regions in a single subject. Although ReHo values of many brain regions can be easily obtained from fMRI data, gene expression data of many brain regions are available only in a few datasets, such as the AHBA provided six donated postmortem brains with 3702 densely sampled expression data of more than 20,000 genes detected by 58,692 probes. An ideal strategy is to investigate the spatial correlation between ReHo and gene expression derived from the same AHBA subject; however, the fMRI data were not acquired in the AHBA data. Based on the fact that the expression patterns of some genes across brain structures are conserved between individuals (Hawrylycz et al., 2015), several pioneer studies have studied the spatial correlations between the gene expression of postmortem AHBA brains and neuroimaging measures of living human brains and found reasonable associations between gene expression and brain imaging phenotypes (Rittman et al., 2016; Romme et al., 2017; Romero-Garcia et al., 2018; Morgan et al., 2019). In this study, we found that the microglial module was selectively enriched for multiple sclerosis and Alzheimer’s disease rather than other brain disorders, which are well consistent with previous findings (Hansen et al., 2018; Voet et al., 2019). In summary, although the spatial correlation analysis is not the best way to investigate the correlation between ReHo and gene expression, it can still provide useful information about the association of gene expression with ReHo.

Although transcription-neuroimaging spatial correlation analysis identified 14 gene modules that were significantly associated with ReHo in both Chinese and non-Chinese samples, more gene modules (*n* = 20) were associated with ReHo in non-Chinese than in Chinese (*n* = 14 for the discovery sample and *n* = 15 for the replication sample 1). The reasons for this discrepancy between different ethnic populations are complex and are so far known to include (1) the ethnic difference in the association between gene expression and ReHo; (2) the difference in ethnic consistency since three of the six AHBA donors are American Caucasian, but none of them are Chinese; and (3) the differences in spatial and temporal resolutions between the two populations, which may influence the resulting ReHo values.

## Conclusion

In conclusion, this transcription-neuroimaging association study revealed that the ReHo of spontaneous brain activity was related to gene expression in cell-type-specific modules of neurons, astrocytes, microglia and epithelial cells, indicating a complex cellular architecture of ReHo. Moreover, we linked gene expression in the microglial module to MS and AD, which may provide possible molecular substrates for ReHo abnormalities in both brain disorders.

## Data Availability Statement

The original contributions presented in the study are included in the article/Supplementary Material, further inquiries can be directed to the corresponding author/s.

## Ethics Statement

The studies involving human participants were reviewed and approved by Medical Research Ethics Committee of Tianjin Medical University. The patients/participants provided their written informed consent to participate in this study. Written informed consent was obtained from the individual(s) for the publication of any potentially identifiable images or data included in this article.

## Author Contributions

JS and CY conceived of the idea. BY, PZ, WL, and ZY analyzed the fMRI data. ZX, HW, ZZ, JW, and PW analyzed the transcription data. JS, BY, and CY wrote the initial draft. All authors agreed with the final version of the manuscript.

## Conflict of Interest

The authors declare that the research was conducted in the absence of any commercial or financial relationships that could be construed as a potential conflict of interest.

## Footnotes

1 http://www.fil.ion.ucl.ac.uk/spm

2 https://www.ncbi.nlm.nih.gov/sra

3 https://www.bioinformatics.babraham.ac.uk/projects/fastqc/

4 https://www.bioinformatics.babraham.ac.uk/projects/trim_galore/

5 http://genetics.wustl.edu/jdlab/psi_package/

6 http://www.webgestalt.org/option.php

## Supplementary Material

Supplementary Figure 1 The mean zReHo maps of discovery sample **(A)**, replication sample 1 **(B)** and replication sample 2 **(C)**.

Supplementary Figure 2 The cluster dendrogram and 30 gene modules clustered following weighted gene coexpression network analysis.

Supplementary Figure 3 The spatial correlation between ME and zReHo of each cell-type-specific module with correlation coefficient and Bonferroni-corrected *P* values in replication sample 1. **(A)** Brown module, **(B)** blue module, **(C)** red module, **(D)** dark orange module, **(E)** yellow module. ME, module eigengene; zReHo, z transformed regional homogeneity.

Supplementary Figure 4 The spatial correlation between ME and zReHo of each cell-type-specific module with correlation coefficient and Bonferroni-corrected *P* values in replication sample 2. **(A)** Brown module, **(B)** blue module, **(C)** red module, **(D)** dark orange module, **(E)** yellow module. ME, module eigengene; zReHo, z transformed regional homogeneity.

Supplementary Figure 5 The group-level zReHo of three cohorts mapping on cortical surface of the HCP Atlas. **(A)** discovery sample, **(B)** replication sample 1, **(C)** replication sample 2. HCP, Human Connectome Project; L, left hemisphere; R, right hemisphere; zReHo, z transformed regional homogeneity.

Supplementary Figure 6 The ME values of cell-type-specific modules mapping on cortical surface of the HCP Atlas. **(A)** ME brown, **(B)** ME blue, **(C)** ME red, **(D)** ME darkorange, **(E)** ME yellow. HCP, Human Connectome Project; L, left hemisphere; ME, module eigengene; R, right hemisphere.

Supplementary Table 1 The 820 neocortical sample coordinates with the corresponding HCP brain regions. HCP, Human Connectome Project; MNI, Montreal Neurological Institute; Parcel Index, parcelation index.

Supplementary Table 2 The normality test results of ME for all modules across neocortical regions. Note: The Kolmogorov-Smirnov test was applied to check the normality of ME for modules across neocortical regions. ME, module eigengene.

Supplementary Table 3 The GWAS datasets of common brain disorders involved in this study. GWAS, genome-wide association studies; IGAP, International Genomics of Alzheimer’s Project; ILAE, International League Against Epilepsy; IMSGC, International Multiple Sclerosis Genetics Consortium; MEGASTROKE, International Stroke Genetics Consortium; PGC, Psychiatric Genomics Consortium; iPSYCH, Integrative Psychiatric Research Consortium.

Supplementary Table 4 The significant ReHo-related modules in the discovery sample and replication samples. Note: the significant modules common to the three samples are labeled with bold font. *r*, correlation coefficients between ME and zReHo; *Pc*, Bonferroni-corrected *P* values; ReHo, regional homogeneity.

Supplementary Table 5 The results of cell-type-specific enrichment analysis in two GSE samples. The significant cell-type-specific ReHo-related modules in both GSE samples are labeled with bold font. Astro, astrocytoma; Endo, endothelium; GSE, gene series expression; Micro, microglia; Neuro, neuron; Oligo, oligodendrocyte; *Pc*, Bonferroni-corrected *P* values; ReHo, regional homogeneity.

Supplementary Table 6 The significant GO terms in ReHo-related cell-type-specific modules. BP, biological process; CC, cellular component; FDR, the corrected *P* values with the false discovery rate method; GO, gene ontology; MF, molecular function; ReHo, regional homogeneity.

Supplementary Table 7 The enrichment results for 10 common brain disorders in this study. The significant modules enriched for brain disorders are labeled with bold font. AD, Alzheimer’s disease; ADHD, attention deficit hyperactivity disorder; ASD, autism spectrum disorder; BP, bipolar disorder; IGAP, International Genomics of Alzheimer’s Project; ILAE, International League Against Epilepsy; IMSGC, International Multiple Sclerosis Genetics Consortium; iPSYCH, Integrative Psychiatric Research Consortium; MDD, major depressive disorder; MEGASTROKE, International Stroke Genetics Consortium; MS, multiple Sclerosis; PD, Parkinson’s disease; PGC, Psychiatric Genomics Consortium; *Pc*, Bonferroni-corrected *P* values; SCZ, schizophrenia.

Supplementary Table 8 The MAGMA gene level analysis results in the microglial module for AD and MS. The significant genes common to both discovery and replication GWAS datasets are labeled with bold font. AD, Alzheimer’s disease; IGAP, International Genomics of Alzheimer’s Project; IMSGC, International Multiple Sclerosis Genetics Consortium; MAGMA, multi-marker analysis of genomic annotation; MS, multiple sclerosis.
