# Supplementary figures and images for: Cell-Type-Specific Gene Modules Related to the Regional Homogeneity of Spontaneous Brain Activity and Their Associations With Common Brain Disorders
Source: Front Neurosci. 2021 Apr 20;15:639527. doi: 10.3389/fnins.2021.639527 (PMC8093778; doi:10.3389/fnins.2021.639527)

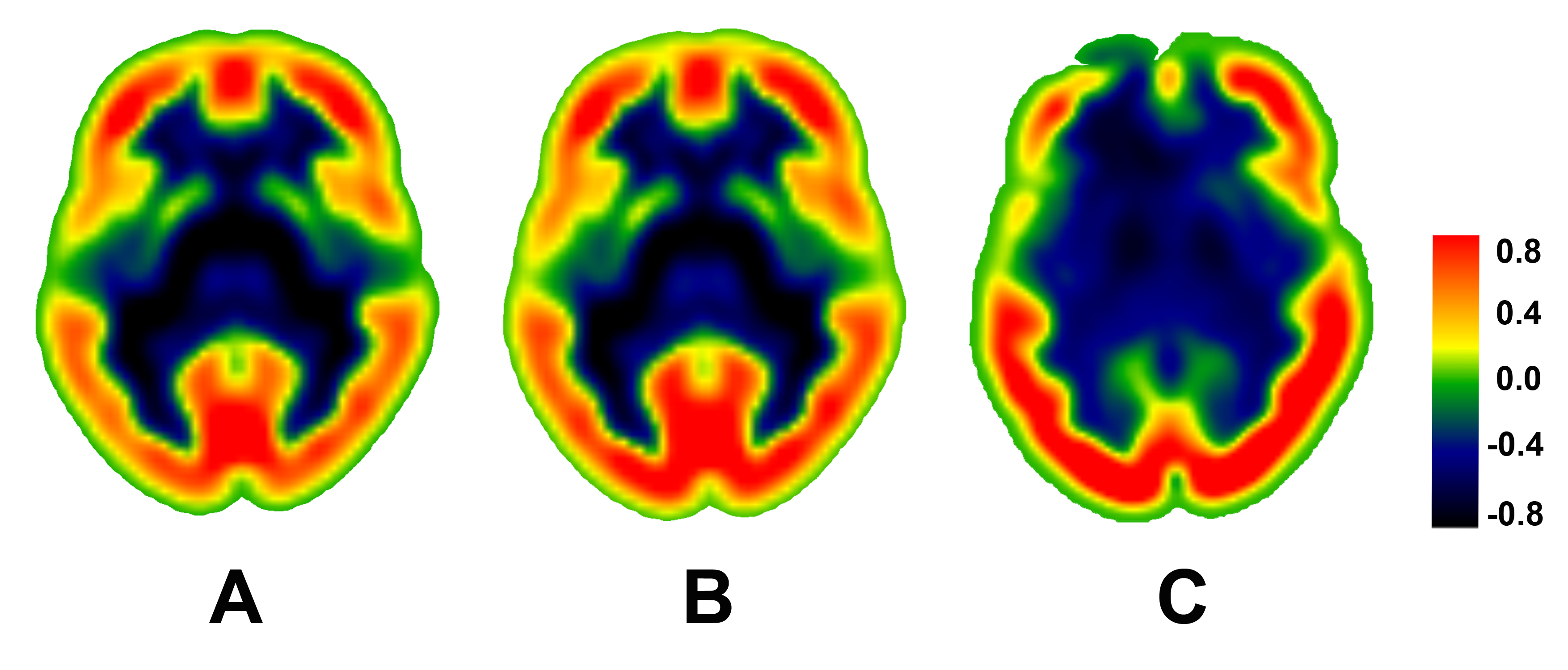

Supplement: Supplementary Figure 1 — The mean zReHo maps of discovery sample (A), replication sample 1 (B) and replication sample 2 (C). [file Image_1.TIF]

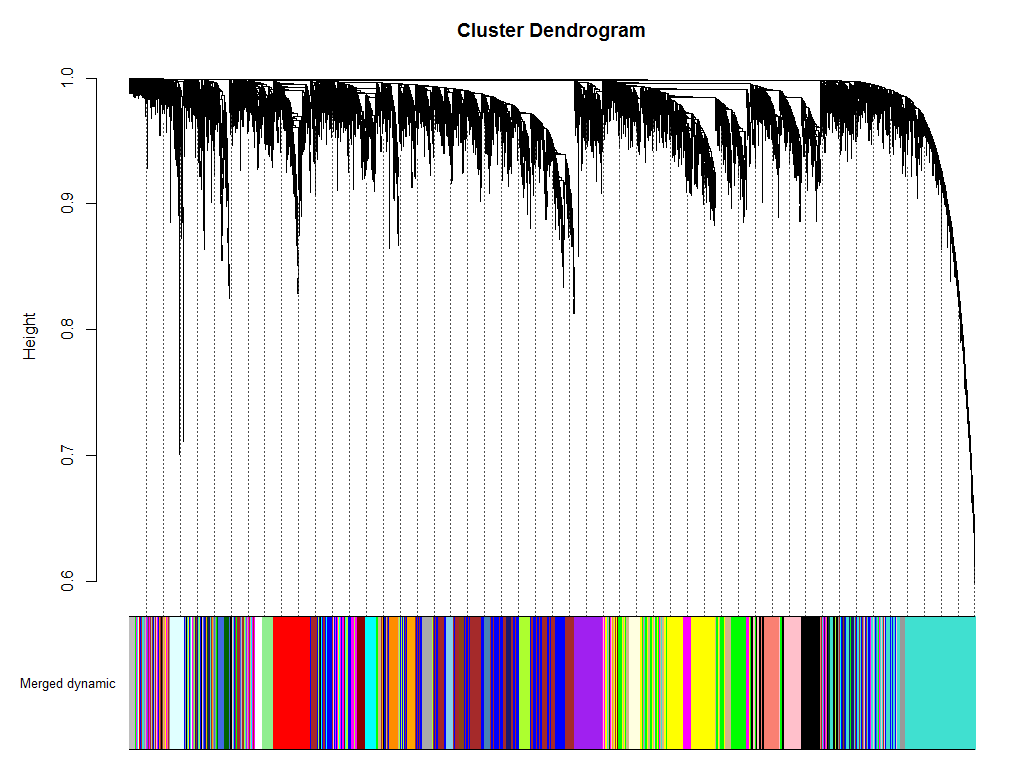

Supplement: Supplementary Figure 2 — The cluster dendrogram and 30 gene modules clustered following weighted gene coexpression network analysis. [file Image_2.TIFF]

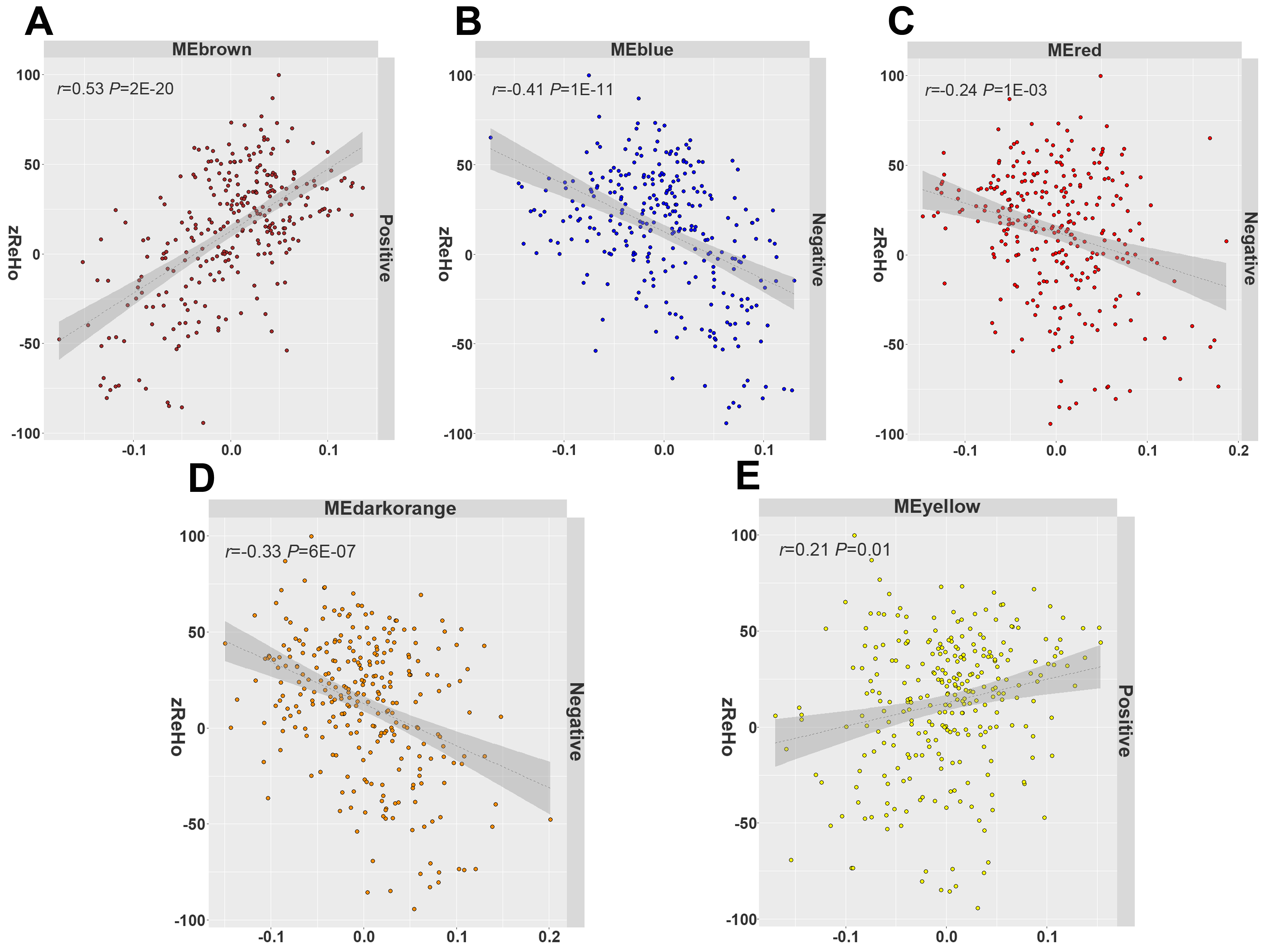

Supplement: Supplementary Figure 3 — The spatial correlation between ME and zReHo of each cell-type-specific module with correlation coefficient and Bonferroni-corrected P values in replication sample 1. (A) Brown module, (B) blue module, (C) red module, (D) dark orange module, (E) yellow module. ME, module eigengene; zReHo, z transformed regional homogeneity. [file Image_3.TIF]

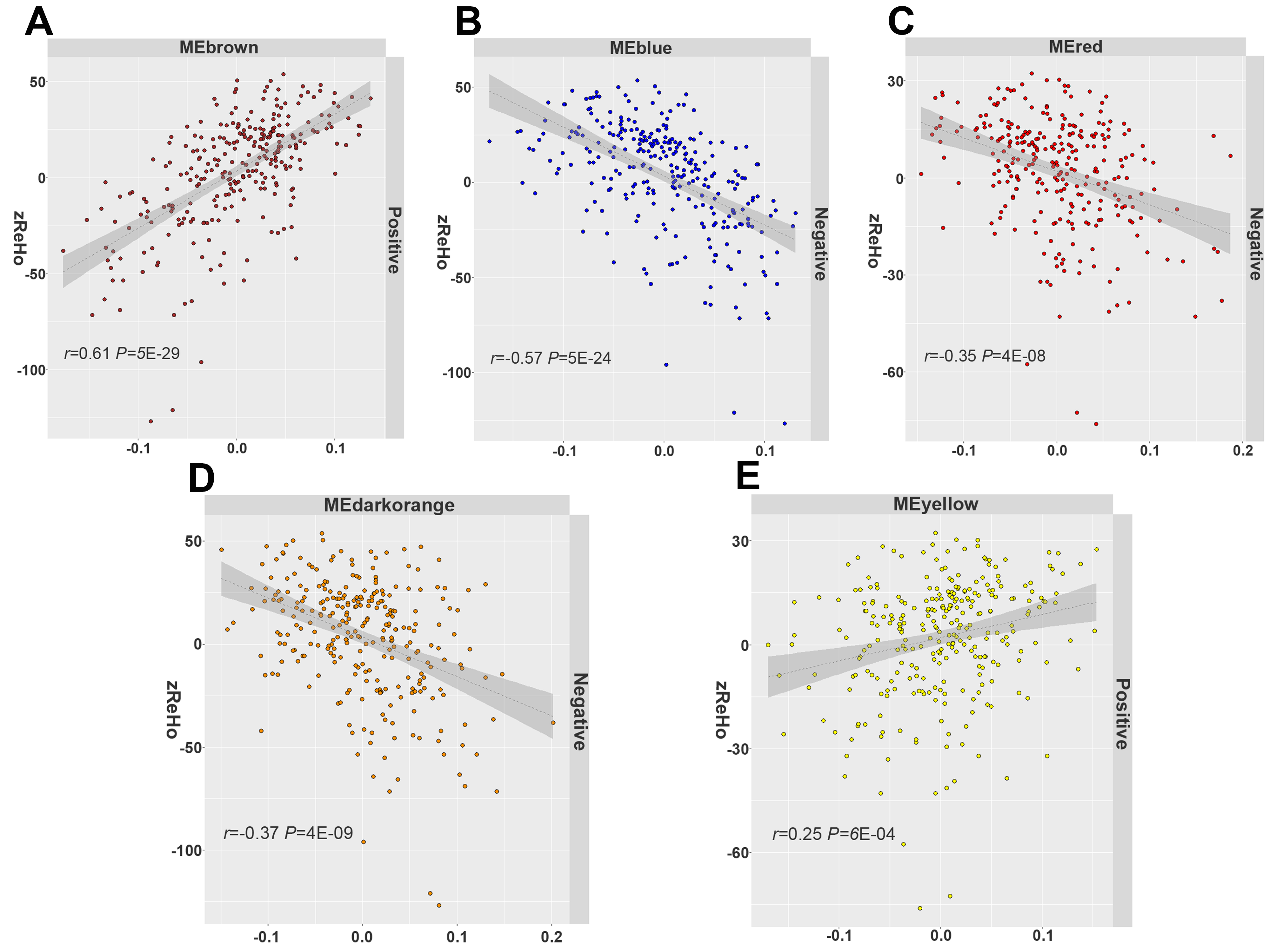

Supplement: Supplementary Figure 4 — The spatial correlation between ME and zReHo of each cell-type-specific module with correlation coefficient and Bonferroni-corrected P values in replication sample 2. (A) Brown module, (B) blue module, (C) red module, (D) dark orange module, (E) yellow module. ME, module eigengene; zReHo, z transformed regional homogeneity. [file Image_4.TIF]

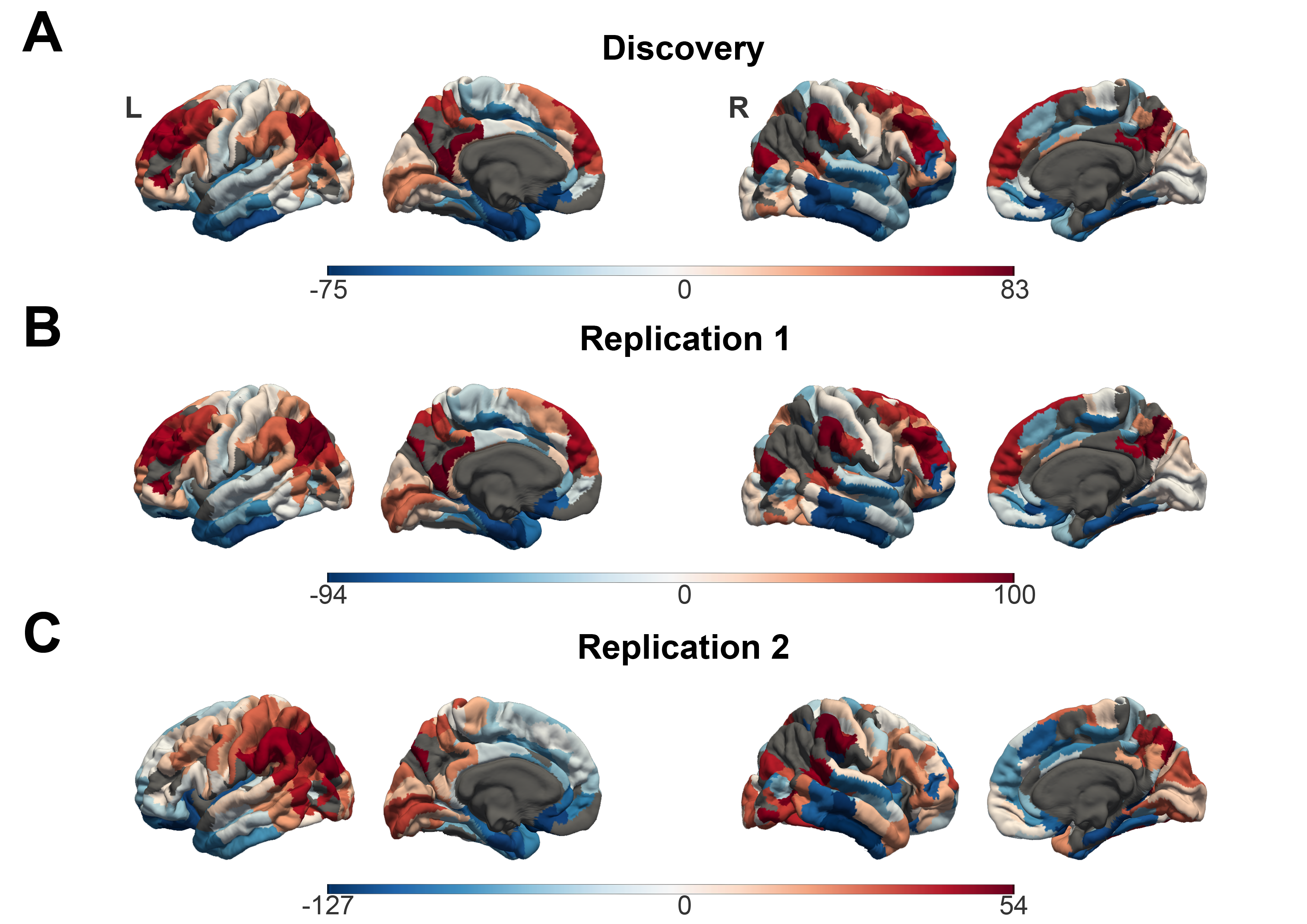

Supplement: Supplementary Figure 5 — The group-level zReHo of three cohorts mapping on cortical surface of the HCP Atlas. (A) discovery sample, (B) replication sample 1, (C) replication sample 2. HCP, Human Connectome Project; L, left hemisphere; R, right hemisphere; zReHo, z transformed regional homogeneity. [file Image_5.TIF]

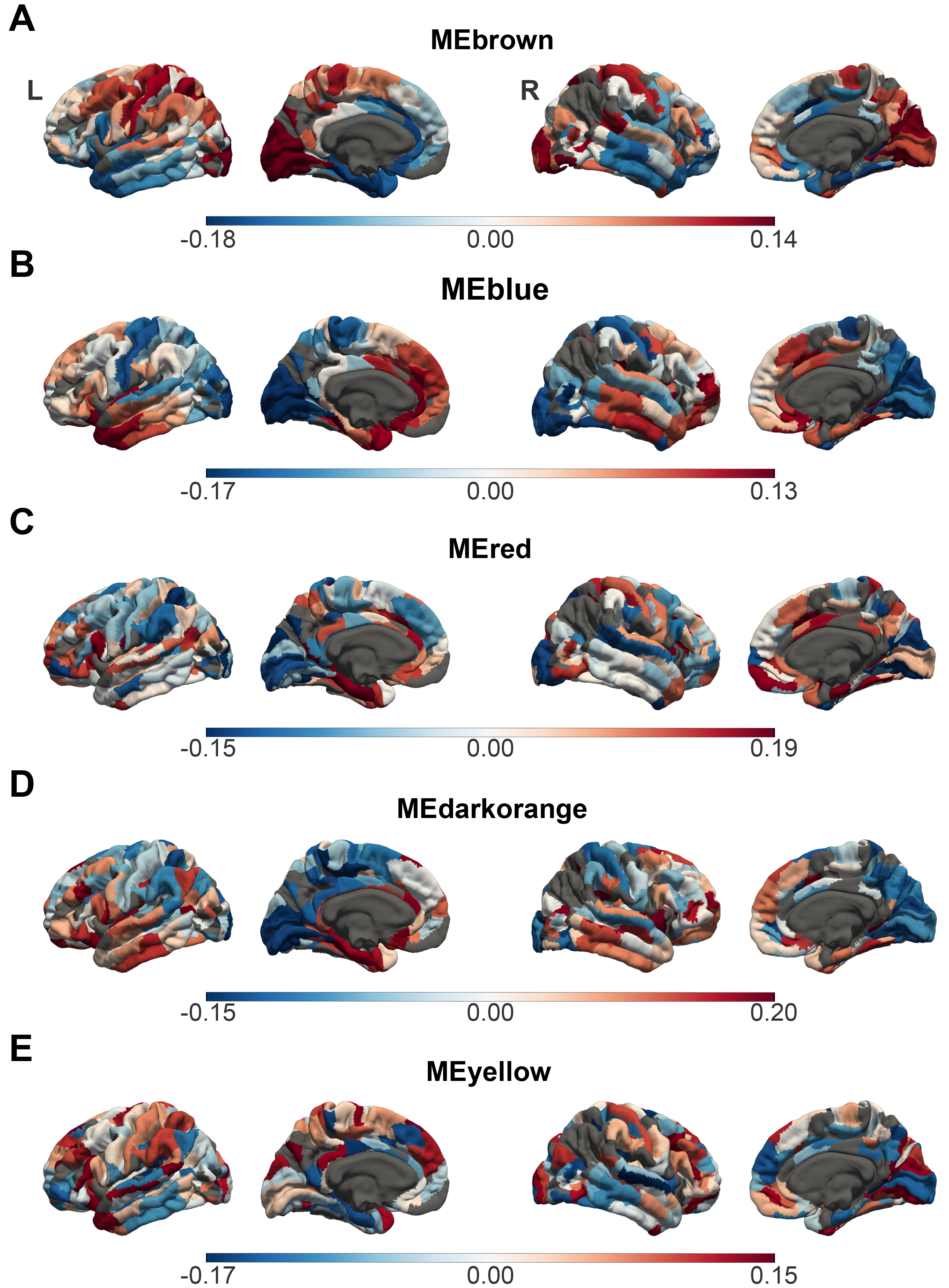

Supplement: Supplementary Figure 6 — The ME values of cell-type-specific modules mapping on cortical surface of the HCP Atlas. (A) ME brown, (B) ME blue, (C) ME red, (D) ME darkorange, (E) ME yellow. HCP, Human Connectome Project; L, left hemisphere; ME, module eigengene; R, right hemisphere. [file Image_6.TIF]
