# Supplementary material for: Cell-Type-Specific Gene Modules Related to the Regional Homogeneity of Spontaneous Brain Activity and Their Associations With Common Brain Disorders
Source: Front Neurosci. 2021 Apr 20;15:639527. doi: 10.3389/fnins.2021.639527 (PMC8093778; doi:10.3389/fnins.2021.639527)
Supplement: Supplementary file 7 [file Table_1.DOC]

**Table S1.** The ethnicity of 600 subjects from the Human Connectome Project.

| **Num.** | **Ethnicity** |
| --- | --- |
| 444 | White |
| 98 | Black or African American |
| 28 | Asian/Native Hawaiian/Other Pacific Islander |
| 17 | More than one race |
| 12 | Hispanic/Latino |
| 1 | American Indian/Alaskan Native |

Note: Num., number of subjects.
