# Supplementary material for: Cell-Type-Specific Gene Modules Related to the Regional Homogeneity of Spontaneous Brain Activity and Their Associations With Common Brain Disorders
Source: Front Neurosci. 2021 Apr 20;15:639527. doi: 10.3389/fnins.2021.639527 (PMC8093778; doi:10.3389/fnins.2021.639527)
Supplement: Supplementary Table 1 — The 820 neocortical sample coordinates with the corresponding HCP brain regions. HCP, Human Connectome Project; MNI, Montreal Neurological Institute; Parcel Index, parcelation index. [file Table_2.DOC]

**Table S1. The 820 neocortical coordinates with their corresponding HCP brain regions.**

| **Parcel Index** | **Area Name** | **Area Description** | **MNI Coordinates** | | |
| --- | --- | --- | --- | --- | --- |
| 1 | L_V1 | Left Primary Visual Cortex | -8 | -74.2 | 7.6 |
| 1 | L_V1 | Left Primary Visual Cortex | -7.4 | -94.1 | -17.8 |
| 1 | L_V1 | Left Primary Visual Cortex | -6.4 | -94.1 | -17.8 |
| 1 | L_V1 | Left Primary Visual Cortex | -6.6 | -62.9 | 8.9 |
| 1 | L_V1 | Left Primary Visual Cortex | -6.9 | -78.8 | 8.7 |
| 1 | L_V1 | Left Primary Visual Cortex | -11.1 | -96.7 | 0.9 |
| 1 | L_V1 | Left Primary Visual Cortex | -27.8 | -98.4 | -9.6 |
| 1 | L_V1 | Left Primary Visual Cortex | -5.8 | -79 | 1.6 |
| 1 | L_V1 | Left Primary Visual Cortex | -4.9 | -62.4 | 7.2 |
| 1 | L_V1 | Left Primary Visual Cortex | -11.2 | -95.4 | 5.5 |
| 4 | L_V2 | Left Second Visual Area | -19.9 | -85.7 | -18.5 |
| 4 | L_V2 | Left Second Visual Area | -2.5 | -68.9 | 19.1 |
| 4 | L_V2 | Left Second Visual Area | -12.4 | -82.9 | -16.6 |
| 4 | L_V2 | Left Second Visual Area | -1.5 | -68.9 | 19.1 |
| 4 | L_V2 | Left Second Visual Area | -6.7 | -67.3 | -3.7 |
| 4 | L_V2 | Left Second Visual Area | -33.6 | -97.9 | 6.8 |
| 4 | L_V2 | Left Second Visual Area | -14.6 | -81.8 | -11.1 |
| 4 | L_V2 | Left Second Visual Area | -11.5 | -67.1 | 0.6 |
| 5 | L_V3 | Left Third Visual Area | -33.7 | -84.1 | -6.2 |
| 5 | L_V3 | Left Third Visual Area | -32.6 | -84 | -3.9 |
| 5 | L_V3 | Left Third Visual Area | -1.5 | -75.9 | 28.5 |
| 5 | L_V3 | Left Third Visual Area | -12.4 | -91.9 | 12.2 |
| 6 | L_V4 | Left Fourth Visual Area | -31.7 | -60 | -4.9 |
| 8 | L_4 | Left Primary Motor Cortex | -53.8 | -10 | 33.8 |
| 8 | L_4 | Left Primary Motor Cortex | -58 | -10.8 | 35 |
| 8 | L_4 | Left Primary Motor Cortex | -38.2 | -17.6 | 41.2 |
| 8 | L_4 | Left Primary Motor Cortex | -37.2 | -22.1 | 53.3 |
| 8 | L_4 | Left Primary Motor Cortex | -41.5 | -23 | 54.5 |
| 8 | L_4 | Left Primary Motor Cortex | -1.3 | -21.7 | 73.1 |
| 8 | L_4 | Left Primary Motor Cortex | -1.2 | -35.3 | 63.8 |
| 8 | L_4 | Left Primary Motor Cortex | -39.4 | -17.9 | 47.9 |
| 8 | L_4 | Left Primary Motor Cortex | -25.9 | -27 | 61.8 |
| 8 | L_4 | Left Primary Motor Cortex | -3.5 | -26.5 | 66.5 |
| 8 | L_4 | Left Primary Motor Cortex | -21.5 | -26.7 | 68.8 |
| 8 | L_4 | Left Primary Motor Cortex | -5.9 | -26.2 | 75.9 |
| 8 | L_4 | Left Primary Motor Cortex | -5.7 | -36.3 | 68.6 |
| 8 | L_4 | Left Primary Motor Cortex | 0 | -35.3 | 63.8 |
| 8 | L_4 | Left Primary Motor Cortex | -6.8 | -36.2 | 70.9 |
| 9 | L_3b | Left Primary Sensory Cortex | -4.6 | -37.1 | 76.9 |
| 9 | L_3b | Left Primary Sensory Cortex | -31.6 | -26.8 | 70 |
| 9 | L_3b | Left Primary Sensory Cortex | -38.2 | -29.5 | 59.6 |
| 9 | L_3b | Left Primary Sensory Cortex | -64 | -12 | 37.9 |
| 10 | L_FEF | Left Frontal Eye Fields | -26.2 | -0.9 | 57.1 |
| 12 | L_55b | Left Area 55b | -44.1 | -1 | 54.8 |
| 13 | L_V3A | Left Area V3A | -6.9 | -91.2 | 29.9 |
| 13 | L_V3A | Left Area V4A | -17 | -93.8 | 24.2 |
| 14 | L_RSC | Left RetroSplenial Complex | -0.1 | -10.1 | 29.6 |
| 14 | L_RSC | Left RetroSplenial Complex | -0.9 | -34.4 | 25.9 |
| 15 | L_POS2 | Left Parieto-Occipital Sulcus Area 2 | -15 | -73.8 | 30.7 |
| 17 | L_IPS1 | Left IntraParietal Sulcus Area 1 | -25.1 | -76 | 37.9 |
| 18 | L_FFC | Left Fusiform Face Complex | -47.5 | -53.3 | -18.5 |
| 18 | L_FFC | Left Fusiform Face Complex | -45.1 | -60.5 | -13.1 |
| 18 | L_FFC | Left Fusiform Face Complex | -39.7 | -44 | -14.6 |
| 20 | L_LO1 | Left Area Lateral Occipital 1 | -48.3 | -85 | 8 |
| 22 | L_PIT | Left Posterior InferoTemporal | -36.8 | -86 | -9.9 |
| 24 | L_A1 | Left Primary Auditory Cortex | -34.4 | -35.5 | 18.8 |
| 25 | L_PSL | Left PeriSylvian Language Area | -59.2 | -54.4 | 24 |
| 25 | L_PSL | Left PeriSylvian Language Area | -67 | -40.9 | 27.5 |
| 26 | L_SFL | Left Superior Frontal Language Area | -1.6 | 7.7 | 59.8 |
| 27 | L_PCV | Left PreCuneus Visual Area | -4.2 | -50.2 | 40.2 |
| 27 | L_PCV | Left PreCuneus Visual Area | -3.1 | -50.2 | 40.3 |
| 27 | L_PCV | Left PreCuneus Visual Area | -2 | -52.1 | 51.1 |
| 28 | L_STV | Left Superior Temporal Visual Area | -55 | -58 | 10.2 |
| 31 | L_POS1 | Left Parieto-Occipital Sulcus Area 1 | -5.6 | -62.9 | 8.9 |
| 32 | L_23d | Left Area 23d | -1.2 | -16.7 | 29.6 |
| 33 | L_v23ab | Left Area ventral 23 a+b | -5.6 | -64.4 | 23.8 |
| 33 | L_v23ab | Left Area ventral 23 a+b | -2.8 | -53.2 | 19.4 |
| 34 | L_d23ab | Left Area dorsal 23 a+b | -2.7 | -35.6 | 33.1 |
| 36 | L_5m | Left Area 5m | -6 | -40.2 | 57.4 |
| 38 | L_23c | Left Area 23c | -12.7 | -13.1 | 39.8 |
| 38 | L_23c | Left Area 23c | -17.4 | -32.1 | 38.8 |
| 38 | L_23c | Left Area 23c | -10.2 | -10 | 37.9 |
| 39 | L_5L | Left Area 5L | -4.4 | -52.6 | 70.1 |
| 40 | L_24dd | Left Dorsal Area 24d | -5.4 | -17.3 | 48.6 |
| 40 | L_24dd | Left Dorsal Area 24d | -6.8 | -21.8 | 43.7 |
| 40 | L_24dd | Left Dorsal Area 24d | -7.9 | -23.9 | 48.6 |
| 40 | L_24dd | Left Dorsal Area 24d | -7.8 | -33.8 | 47.1 |
| 41 | L_24dv | Left Ventral Area 24d | -7.1 | -0.9 | 47.7 |
| 42 | L_7AL | Left Lateral Area 7A | -16.8 | -50.2 | 70.2 |
| 42 | L_7AL | Left Lateral Area 7A | -10 | -54.8 | 75 |
| 43 | L_SCEF | Left Supplementary and Cingulate Eye Field | -6.6 | 17.9 | 48.8 |
| 43 | L_SCEF | Left Supplementary and Cingulate Eye Field | -3 | -3.7 | 70.8 |
| 43 | L_SCEF | Left Supplementary and Cingulate Eye Field | -3.2 | -11.6 | 65.6 |
| 43 | L_SCEF | Left Supplementary and Cingulate Eye Field | -2.5 | -12.5 | 58.2 |
| 44 | L_6ma | Left Area 6m anterior | -18.5 | 6.7 | 57.3 |
| 44 | L_6ma | Left Area 6m anterior | -24.9 | 7.4 | 60.4 |
| 44 | L_6ma | Left Area 6m anterior | -25.2 | -4.8 | 68.3 |
| 45 | L_7Am | Left Medial Area 7A | -5.3 | -51.6 | 64 |
| 46 | L_7PL | Left Lateral Area 7P | -20.4 | -66.4 | 63.1 |
| 47 | L_7PC | Left Area 7PC | -33.6 | -44.5 | 66.9 |
| 48 | L_LIPv | Left Area Lateral IntraParietal ventral | -38.3 | -62.5 | 58.6 |
| 48 | L_LIPv | Left Area Lateral IntraParietal ventral | -29.9 | -62.3 | 56.7 |
| 49 | L_VIP | Left Ventral IntraParietal Complex | -19.7 | -72.9 | 60.1 |
| 49 | L_VIP | Left Ventral IntraParietal Complex | -30 | -61.1 | 60.1 |
| 50 | L_MIP | Left Medial IntraParietal Area | -18.4 | -70.3 | 52.6 |
| 50 | L_MIP | Left Medial IntraParietal Area | -17.3 | -70.2 | 52.7 |
| 51 | L_1 | Left Area 1 | -57.3 | -23.4 | 54.1 |
| 51 | L_1 | Left Area 1 | -52.1 | -26.5 | 50.4 |
| 51 | L_1 | Left Area 1 | -45.6 | -20.8 | 62.9 |
| 51 | L_1 | Left Area 1 | -39.4 | -26.1 | 67.4 |
| 51 | L_1 | Left Area 1 | -44.6 | -20.8 | 62.9 |
| 51 | L_1 | Left Area 1 | -50.7 | -17.7 | 58.5 |
| 52 | L_2 | Left Area 2 | -53.2 | -27.3 | 34.8 |
| 52 | L_2 | Left Area 2 | -54.3 | -31.1 | 45.6 |
| 52 | L_2 | Left Area 2 | -48.1 | -35.5 | 53.3 |
| 52 | L_2 | Left Area 2 | -43.5 | -46.3 | 51.7 |
| 52 | L_2 | Left Area 2 | -40.3 | -34.2 | 49.4 |
| 53 | L_3a | Left Area 3a | -21.4 | -22.7 | 58.2 |
| 54 | L_6d | Left Dorsal area 6 | -40.1 | -11 | 62.3 |
| 54 | L_6d | Left Dorsal area 6 | -35.2 | -27.4 | 62.2 |
| 54 | L_6d | Left Dorsal area 6 | -36.1 | -18.7 | 66.1 |
| 55 | L_6mp | Left Area 6mp | -21.2 | -13.9 | 66.7 |
| 55 | L_6mp | Left Area 6mp | -19.2 | -18.7 | 73.3 |
| 56 | L_6v | Left Ventral Area 6 | -56.8 | -4.4 | 42.8 |
| 56 | L_6v | Left Ventral Area 6 | -58.8 | 1.2 | 34.9 |
| 57 | L_p24pr | Left Area Posterior 24 prime | -7.1 | 1.4 | 36.1 |
| 57 | L_p24pr | Left Area Posterior 24 prime | -5.3 | -12.7 | 36.6 |
| 57 | L_p24pr | Left Area Posterior 24 prime | -4.6 | -9.9 | 37.9 |
| 57 | L_p24pr | Left Area Posterior 24 prime | -7 | -1.1 | 41.8 |
| 58 | L_33pr | Left Area 33 prime | -3 | -4.9 | 28.4 |
| 58 | L_33pr | Left Area 33 prime | -0.5 | 11.6 | 24.3 |
| 58 | L_33pr | Left Area 33 prime | -1.4 | -1.2 | 34.7 |
| 60 | L_p32pr | Left Area p32 prime | -8.9 | 13.3 | 27.1 |
| 60 | L_p32pr | Left Area p32 prime | -8.3 | 16.3 | 37.8 |
| 61 | L_a24 | Left Area a24 | -7.2 | 38.2 | -7.8 |
| 61 | L_a24 | Left Area a24 | -7.3 | 41.7 | 13.1 |
| 61 | L_a24 | Left Area a24 | -5 | 41.3 | 1.4 |
| 62 | L_d32 | Left Area dorsal 32 | 0.4 | 44.6 | 32.7 |
| 62 | L_d32 | Left Area dorsal 32 | -3 | 43.3 | 27.1 |
| 63 | L_8BM | Left Area 8BM | 0 | 28.4 | 42.5 |
| 65 | L_10r | Left Area 10r | -3.8 | 46.8 | -12.7 |
| 65 | L_10r | Left Area 10r | -7.4 | 56.5 | -3.6 |
| 65 | L_10r | Left Area 10r | -6.3 | 56.4 | -7.1 |
| 66 | L_47m | Left Area 47m | -31.6 | 33.6 | -10.9 |
| 67 | L_8Av | Left Area 8Av | -25.6 | 18.9 | 53.7 |
| 67 | L_8Av | Left Area 8Av | -36.2 | 17.2 | 35.8 |
| 67 | L_8Av | Left Area 8Av | -40.9 | 14 | 53.3 |
| 67 | L_8Av | Left Area 8Av | -36.2 | -1.3 | 50 |
| 68 | L_8Ad | Left Area 8Ad | -18.2 | 20.4 | 54.8 |
| 68 | L_8Ad | Left Area 8Ad | -13.7 | 29.3 | 50.9 |
| 68 | L_8Ad | Left Area 8Ad | -12.7 | 29.4 | 51 |
| 68 | L_8Ad | Left Area 8Ad | -11.6 | 28.5 | 47.8 |
| 68 | L_8Ad | Left Area 8Ad | -14.2 | 30.9 | 54.2 |
| 68 | L_8Ad | Left Area 8Ad | -15.3 | 29.6 | 48.4 |
| 69 | L_9m | Left Area 9 Middle | 1.7 | 57.3 | 22.5 |
| 69 | L_9m | Left Area 9 Middle | 0.4 | 44.8 | 33.8 |
| 70 | L_8BL | Left Area 8B Lateral | -14.6 | 35.7 | 54.2 |
| 70 | L_8BL | Left Area 8B Lateral | -2.8 | 42.7 | 50.9 |
| 71 | L_9p | Left Area 9 Posterior | -26.1 | 41.3 | 47.3 |
| 71 | L_9p | Left Area 9 Posterior | -21.9 | 42 | 37.2 |
| 72 | 10d | Left Area 10d | -2.3 | 64.8 | 9.8 |
| 72 | 10d | Left Area 10d | -1.2 | 64.9 | 9.8 |
| 72 | 10d | Left Area 10d | -9.7 | 56.7 | 5.9 |
| 72 | 10d | Left Area 10d | -5.3 | 66.6 | 3.8 |
| 73 | L_8C | Left Area 8C | -49.1 | 7.4 | 32.9 |
| 73 | L_8C | Left Area 8C | -48.2 | 2.7 | 31.5 |
| 73 | L_8C | Left Area 8C | -44.9 | 7 | 30.8 |
| 73 | L_8C | Left Area 8C | -56.5 | 19.5 | 28 |
| 73 | L_8C | Left Area 8C | -55.4 | 19.5 | 28 |
| 73 | L_8C | Left Area 8C | -53.2 | 19.6 | 30.3 |
| 74 | L_44 | Left Area 44 | -53.4 | 6.4 | -4 |
| 74 | L_44 | Left Area 44 | -54.2 | 16.7 | 14.2 |
| 75 | L_45 | Left Area 45 | -55.2 | 18.3 | -0.7 |
| 75 | L_45 | Left Area 45 | -47.8 | 17.8 | -7.2 |
| 75 | L_45 | Left Area 45 | -45.6 | 22.4 | -2.4 |
| 75 | L_45 | Left Area 45 | -52 | 17.8 | -2.8 |
| 75 | L_45 | Left Area 45 | -42.1 | 35.9 | -1.4 |
| 75 | L_45 | Left Area 45 | -43 | 35.2 | 0.6 |
| 76 | L_47l | LeftArea 47l (47 lateral) | -38.5 | 35.2 | -1.7 |
| 77 | L_a47r | Left Area anterior 47r | -34.1 | 59.2 | -1.1 |
| 78 | L_6r | Left Rostral Area 6 | -50.7 | 7.3 | 22 |
| 80 | L_IFJp | Left Area IFJp | -49.6 | 7.4 | 22 |
| 81 | L_IFSp | Left Area IFSp | -54.1 | 20 | 22.6 |
| 81 | L_IFSp | Left Area IFSp | -53 | 20 | 22.6 |
| 83 | L_p9-46v | Left Area posterior 9-46v | -48.8 | 35.8 | 21.8 |
| 84 | L_46 | Left Area 46 | -46.4 | 30.7 | 20.7 |
| 84 | L_46 | Left Area 46 | -41.9 | 40.9 | 17.8 |
| 84 | L_46 | Left Area 46 | -28.8 | 41.9 | 29.6 |
| 86 | L_9-46d | Left Area 9-46d | -31.5 | 36.7 | 30.1 |
| 87 | L_9a | Left Area 9 anterior | -25.9 | 48.1 | 31.4 |
| 87 | L_9a | Left Area 9 anterior | -24.9 | 48.2 | 31.4 |
| 87 | L_9a | Left Area 9 anterior | -16.6 | 57.5 | 30.5 |
| 87 | L_9a | Left Area 9 anterior | -18.9 | 57.4 | 29.4 |
| 87 | L_9a | Left Area 9 anterior | -16.6 | 57.4 | 29.4 |
| 87 | L_9a | Left Area 9 anterior | -24.4 | 44.3 | 35.3 |
| 87 | L_9a | Left Area 9 anterior | -15.5 | 57.5 | 30.6 |
| 90 | L_10pp | Left Polar 10p | -11 | 54.2 | -17.6 |
| 90 | L_10pp | Left Polar 10p | -10 | 54.2 | -17.6 |
| 90 | L_10pp | Left Polar 10p | -16.4 | 63.6 | -13.7 |
| 90 | L_10pp | Left Polar 10p | -9.6 | 63.7 | -14.8 |
| 90 | L_10pp | Left Polar 10p | -9.7 | 63.8 | -12.5 |
| 90 | L_10pp | Left Polar 10p | -8.5 | 63.8 | -12.5 |
| 90 | L_10pp | Left Polar 10p | -15.3 | 63.9 | -6.6 |
| 90 | L_10pp | Left Polar 10p | -8.5 | 63.7 | -14.8 |
| 91 | L_11l | Left Area 11l | -35.5 | 43.8 | -9.5 |
| 91 | L_11l | Left Area 11l | -32.4 | 44.4 | -7.3 |
| 91 | L_11l | Left Area 11l | -19.6 | 51.6 | -11.4 |
| 92 | L_13l | Left Area 13l | -23.9 | 36.2 | -13.6 |
| 92 | L_13l | Left Area 13l | -20.4 | 24.1 | -16 |
| 93 | L_OFC | Left Orbital Frontal Complex | -7 | 44.8 | -20.2 |
| 93 | L_OFC | Left Orbital Frontal Complex | -10 | 10.1 | -18.3 |
| 93 | L_OFC | Left Orbital Frontal Complex | -8.5 | 30 | -22 |
| 93 | L_OFC | Left Orbital Frontal Complex | -10.4 | 36.1 | -21.8 |
| 93 | L_OFC | Left Orbital Frontal Complex | -8.1 | 36.2 | -20.6 |
| 93 | L_OFC | Left Orbital Frontal Complex | -11.4 | 24.3 | -13.6 |
| 94 | L_47s | Left Area 47s | -46.9 | 14.3 | -12.1 |
| 96 | L_6a | Left Area 6 anterior | -31.4 | -0.6 | 47.1 |
| 96 | L_6a | Left Area 6 anterior | -19.4 | -10.4 | 65 |
| 96 | L_6a | Left Area 6 anterior | -28.4 | -8.5 | 58.9 |
| 96 | L_6a | Left Area 6 anterior | -19.5 | -0.6 | 64.1 |
| 97 | L_i6-8 | Left Inferior 6-8 Transitional Area | -32.4 | 3 | 56.5 |
| 97 | L_i6-8 | Left Inferior 6-9 Transitional Area | -22.8 | 6.6 | 57.2 |
| 97 | L_i6-8 | Left Inferior 6-10 Transitional Area | -35.3 | 14.2 | 55.7 |
| 97 | L_i6-8 | Left Inferior 6-11 Transitional Area | -25.1 | -0.7 | 64.1 |
| 99 | L_43 | Left Area 43 | -64.4 | -10.2 | 4.4 |
| 99 | L_43 | Left Area 43 | -63.2 | -6.1 | 11.5 |
| 99 | L_43 | Left Area 43 | -55.1 | 5.9 | 13.9 |
| 101 | L_OP1 | Left Area OP1/SII | -49.2 | -11.7 | 8.4 |
| 101 | L_OP1 | Left Area OP1/SII | -54.8 | -13 | 5 |
| 102 | L_OP2-3 | Left Area OP2-3/VS | -32.4 | -23.1 | 17.7 |
| 105 | L_PFcm | Left Area PFcm | -57.6 | -34.7 | 19.3 |
| 106 | L_PoI2 | Left Posterior Insular Area 2 | -38 | -11.8 | 0.8 |
| 107 | L_TA2 | Left Area TA2 | -49.3 | 5.3 | -6.1 |
| 108 | L_FOP4 | Left Frontal Opercular Area 4 | -33.1 | 14 | 10.7 |
| 108 | L_FOP4 | Left Frontal Opercular Area 4 | -36.2 | 21.4 | 10.2 |
| 110 | L_Pir_ROI | Left Pirform Cortex | -28 | 5.4 | -15.5 |
| 111 | L_AVI | Left Anterior Ventral Insular Area | -35 | 24 | 3.2 |
| 111 | L_AVI | Left Anterior Ventral Insular Area | -33.9 | 24.1 | 3.2 |
| 114 | L_FOP3 | Left Frontal Opercular Area 3 | -37.7 | 0.6 | 14.3 |
| 114 | L_FOP3 | Left Frontal Opercular Area 3 | -33.5 | -0.7 | 13.5 |
| 114 | L_FOP3 | Left Frontal Opercular Area 3 | -33.8 | 6.1 | 10.4 |
| 116 | L_PFt | Left Area PFt | -48.9 | -24.8 | 35.6 |
| 117 | L_AIP | Left Anterior IntraParietal Area | -35.5 | -38 | 42.8 |
| 117 | L_AIP | Left Anterior IntraParietal Area | -46.1 | -40.9 | 40.9 |
| 118 | L_EC | Left Entorhinal Cortex | -25.6 | -22.4 | -23.8 |
| 118 | L_EC | Left Entorhinal Cortex | -20.9 | -11.5 | -32.8 |
| 119 | L_PreS | Left PreSubiculum | -20.4 | -22 | -22.6 |
| 119 | L_PreS | Left PreSubiculum | -12.3 | -39.2 | -9.1 |
| 119 | L_PreS | Left PreSubiculum | -18.6 | -29.5 | -14.7 |
| 120 | L_H | Left Hippocampus | -26.4 | -12.5 | -27.9 |
| 121 | L_ProS | Left ProStriate Area | -10.6 | -48.6 | -1.1 |
| 121 | L_ProS | Left ProStriate Area | -10.6 | -46.3 | -0.1 |
| 122 | L_PeEc | Left Perirhinal Ectorhinal Cortex | -34.1 | -23.7 | -28.2 |
| 122 | L_PeEc | Left Perirhinal Ectorhinal Cortex | -25.1 | -0.5 | -32.4 |
| 122 | L_PeEc | Left Perirhinal Ectorhinal Cortex | -38.1 | -14.2 | -33.5 |
| 122 | L_PeEc | Left Perirhinal Ectorhinal Cortex | -33.9 | -15 | -36.6 |
| 122 | L_PeEc | Left Perirhinal Ectorhinal Cortex | -29.6 | -12.9 | -29 |
| 122 | L_PeEc | Left Perirhinal Ectorhinal Cortex | -24 | -0.5 | -32.3 |
| 122 | L_PeEc | Left Perirhinal Ectorhinal Cortex | -27.2 | -1.1 | -34.5 |
| 122 | L_PeEc | Left Perirhinal Ectorhinal Cortex | -27.6 | -11.5 | -30.5 |
| 122 | L_PeEc | Left Perirhinal Ectorhinal Cortex | -30.9 | -13 | -38.6 |
| 122 | L_PeEc | Left Perirhinal Ectorhinal Cortex | -31 | -11.7 | -32.8 |
| 124 | L_PBelt | Left ParaBelt Complex | -55.6 | -36.9 | 10.7 |
| 124 | L_PBelt | Left ParaBelt Complex | -45 | -36.6 | 15.4 |
| 125 | L_A5 | Left Auditory 5 Complex | -62.7 | -28.6 | 1.2 |
| 125 | L_A5 | Left Auditory 5 Complex | -60.8 | 6.5 | -3.1 |
| 126 | L_PHA1 | Left ParaHippocampal Area 1 | -25.3 | -29.6 | -17 |
| 127 | L_PHA3 | Left ParaHippocampal Area 3 | -33.1 | -31.1 | -24 |
| 128 | L_STSda | Left Area STSd anterior | -52 | -21 | -12.3 |
| 128 | L_STSda | Left Area STSd anterior | -48.2 | 2.3 | -20 |
| 128 | L_STSda | Left Area STSd anterior | -51.4 | 1.7 | -22.2 |
| 128 | L_STSda | Left Area STSd anterior | -49.1 | -14.5 | -9 |
| 129 | L_STSdp | Left Area STSd posterior | -55.6 | -38.1 | 6.5 |
| 129 | L_STSdp | Left Area STSd posterior | -44.1 | -44.8 | 9.1 |
| 129 | L_STSdp | Left Area STSd posterior | -57.7 | -37.8 | 7.5 |
| 129 | L_STSdp | Left Area STSd posterior | -54.6 | -26.4 | -2.1 |
| 130 | L_STSvp | Left Area STSv posterior | -57.5 | -31.8 | -7.1 |
| 130 | L_STSvp | Left Area STSv posterior | -60.7 | -31.6 | -6.1 |
| 130 | L_STSvp | Left Area STSv posterior | -46.3 | -45.7 | 5.9 |
| 130 | L_STSvp | Left Area STSv posterior | -57.9 | -28.8 | -6.6 |
| 131 | L_TGd | Left Area TG dorsal | -49.4 | -0.8 | -31.8 |
| 131 | L_TGd | Left Area TG dorsal | -46.2 | -1.8 | -36 |
| 131 | L_TGd | Left Area TG dorsal | -41.8 | 7.7 | -37.7 |
| 131 | L_TGd | Left Area TG dorsal | -25.9 | 9.2 | -33.1 |
| 131 | L_TGd | Left Area TG dorsal | -24.8 | 9.2 | -33 |
| 131 | L_TGd | Left Area TG dorsal | -41.6 | 19.2 | -20.3 |
| 132 | L_TE1a | Left Area TE1 anterior | -54.9 | -11.5 | -22 |
| 132 | L_TE1a | Left Area TE1 anterior | -55.9 | -9.3 | -13.5 |
| 132 | L_TE1a | Left Area TE1 anterior | -51.7 | -11.2 | -20.9 |
| 132 | L_TE1a | Left Area TE1 anterior | -52.4 | 2.5 | -19 |
| 133 | L_TE1p | Left Area TE1 posterior | -58.9 | -43.3 | -5 |
| 133 | L_TE1p | Left Area TE1 posterior | -57.9 | -47.1 | 1.4 |
| 133 | L_TE1p | Left Area TE1 posterior | -54.9 | -51.2 | -10.1 |
| 133 | L_TE1p | Left Area TE1 posterior | -55.8 | -47.4 | 0.4 |
| 133 | L_TE1p | Left Area TE1 posterior | -57.9 | -44.4 | -9.2 |
| 133 | L_TE1p | Left Area TE1 posterior | -63.1 | -60.4 | -0.2 |
| 133 | L_TE1p | Left Area TE1 posterior | -58.8 | -46.4 | -9.7 |
| 133 | L_TE1p | Left Area TE1 posterior | -58.7 | -46.5 | -14.4 |
| 134 | L_TE2a | Left Area TE2 anterior | -52.4 | -35.3 | -20.9 |
| 134 | L_TE2a | Left Area TE2 anterior | -53.5 | -37.3 | -28.4 |
| 134 | L_TE2a | Left Area TE2 anterior | -52 | -22.1 | -16.6 |
| 134 | L_TE2a | Left Area TE2 anterior | -52 | -23.2 | -20.9 |
| 134 | L_TE2a | Left Area TE2 anterior | -55.3 | -25.9 | -30.6 |
| 134 | L_TE2a | Left Area TE2 anterior | -55.2 | -23.6 | -22 |
| 134 | L_TE2a | Left Area TE2 anterior | -53.9 | -12.3 | -25.2 |
| 134 | L_TE2a | Left Area TE2 anterior | -50.7 | -12 | -24.1 |
| 134 | L_TE2a | Left Area TE2 anterior | -57.7 | -31.6 | -26.4 |
| 134 | L_TE2a | Left Area TE2 anterior | -51.2 | -13.9 | -23.3 |
| 134 | L_TE2a | Left Area TE2 anterior | -51.2 | -14 | -28 |
| 135 | L_TF | Left Area TF | -42.9 | -35.7 | -22.8 |
| 135 | L_TF | Left Area TF | -35.5 | -36.6 | -27 |
| 135 | L_TF | Left Area TF | -42.5 | -21.4 | -18.8 |
| 135 | L_TF | Left Area TF | -44.4 | -14.9 | -35.7 |
| 135 | L_TF | Left Area TF | -41.2 | -12.6 | -27.1 |
| 135 | L_TF | Left Area TF | -32.5 | -1.8 | -36.8 |
| 135 | L_TF | Left Area TF | -37.7 | -11.7 | -30.5 |
| 135 | L_TF | Left Area TF | -40 | -11.8 | -31.7 |
| 135 | L_TF | Left Area TF | -45.4 | -31.2 | -19.3 |
| 135 | L_TF | Left Area TF | -39.8 | -31 | -17 |
| 136 | L_TE2p | Left Area TE2 posterior | -51.3 | -37.3 | -28.3 |
| 136 | L_TE2p | Left Area TE2 posterior | -45.2 | -43.2 | -13.6 |
| 136 | L_TE2p | Left Area TE2 posterior | -54.9 | -52.6 | -15.5 |
| 136 | L_TE2p | Left Area TE2 posterior | -46.5 | -53.3 | -18.5 |
| 136 | L_TE2p | Left Area TE2 posterior | -43.1 | -44.1 | -14.6 |
| 137 | L_PHT | Left Area PHT | -56.1 | -58 | 10.2 |
| 137 | L_PHT | Left Area PHT | -54.1 | -62.4 | -6.9 |
| 137 | L_PHT | Left Area PHT | -64.3 | -59.2 | 2.1 |
| 138 | L_PH | Left Area PH | -42.4 | -60.7 | -1.3 |
| 138 | L_PH | Left Area PH | -50.9 | -61.8 | -4.7 |
| 138 | L_PH | Left Area PH | -40.4 | -62.7 | -8.8 |
| 138 | L_PH | Left Area PH | -41.5 | -64.1 | -14.1 |
| 138 | L_PH | Left Area PH | -50.6 | -74.4 | -0.1 |
| 139 | L_TPOJ1 | Left Area TemporoParietoOccipital Junction 1 | -66.3 | -42.9 | 13.9 |
| 139 | L_TPOJ1 | Left Area TemporoParietoOccipital Junction 1 | -64.5 | -42.6 | 8.8 |
| 139 | L_TPOJ1 | Left Area TemporoParietoOccipital Junction 1 | -67.8 | -43.9 | 3 |
| 139 | L_TPOJ1 | Left Area TemporoParietoOccipital Junction 1 | -62.3 | -42.5 | 8.8 |
| 140 | L_TPOJ2 | Left Area TemporoParietoOccipital Junction 1 | -53.3 | -57.3 | 22 |
| 141 | L_TPOJ3 | Left Area TemporoParietoOccipital Junction 1 | -45.2 | -72.4 | 23.4 |
| 141 | L_TPOJ3 | Left Area TemporoParietoOccipital Junction 1 | -40.8 | -69.9 | 30.2 |
| 141 | L_TPOJ3 | Left Area TemporoParietoOccipital Junction 1 | -44.2 | -71.1 | 28 |
| 141 | L_TPOJ3 | Left Area TemporoParietoOccipital Junction 1 | -44.1 | -72.4 | 23.4 |
| 141 | L_TPOJ3 | Left Area TemporoParietoOccipital Junction 1 | -41.9 | -71.1 | 28 |
| 143 | L_PGp | Left Area PGp | -36.7 | -84.3 | 25.7 |
| 143 | L_PGp | Left Area PGp | -35.7 | -84.3 | 25.7 |
| 143 | L_PGp | Left Area PGp | -46.2 | -83.3 | 25.5 |
| 145 | L_IP1 | Left Area IntraParietal 1 | -35.1 | -62.6 | 45.2 |
| 145 | L_IP1 | Left Area IntraParietal 1 | -34 | -62.6 | 45.2 |
| 146 | L_IP0 | Left Area IntraParietal 0 | -27.3 | -79.2 | 39.4 |
| 147 | L_PFop | Left Area PF opercular | -64 | -38.7 | 25.5 |
| 147 | L_PFop | Left Area PF opercular | -60.8 | -34.5 | 20.3 |
| 147 | L_PFop | Left Area PF opercular | -63 | -38.7 | 25.5 |
| 147 | L_PFop | Left Area PF opercular | -65.1 | -38.8 | 25.4 |
| 148 | L_PF_ROI | Left Area PF Complex | -63.7 | -37.2 | 37.8 |
| 148 | L_PF_ROI | Left Area PF Complex | -59.3 | -36.9 | 46.1 |
| 149 | L_PFm | Left Area PFm Complex | -56.1 | -60.3 | 43.1 |
| 150 | L_PGi | Left Area Pgi | -43.9 | -76.3 | 27.4 |
| 150 | L_PGi | Left Area Pgi | -45.6 | -62.6 | 33.7 |
| 150 | L_PGi | Left Area Pgi | -50 | -54.7 | 32.4 |
| 151 | L_PGs | Left Area PGs | -35.4 | -76.5 | 51.1 |
| 151 | L_PGs | Left Area PGs | -32.9 | -82.6 | 40.8 |
| 151 | L_PGs | Left Area PGs | -42.1 | -67.3 | 44.1 |
| 153 | L_VMV1 | Left VentroMedial Visual Area 1 | -18.4 | -54.2 | -3 |
| 154 | L_VMV3 | Left VentroMedial Visual Area 3 | -29.8 | -61.7 | -9.8 |
| 155 | L_PHA2 | Left ParaHippocampal Area 2 | -32.2 | -31.6 | -16.6 |
| 155 | L_PHA2 | Left ParaHippocampal Area 2 | -32.4 | -37.9 | -15.4 |
| 155 | L_PHA2 | Left ParaHippocampal Area 2 | -28.1 | -37 | -12.1 |
| 156 | L_V4t | Left Area V4t | -55.1 | -74.4 | -0.1 |
| 159 | L_LO3 | Left Area Lateral Occipital3 | -42.9 | -79.1 | 16.7 |
| 160 | L_VMV2 | Left VentroMedial Visual Area 2 | -24.5 | -59.1 | -4.6 |
| 162 | L_31a | Left Area 31a | -7.7 | -34 | 41.2 |
| 164 | L_25 | Left Area 25 | -3.6 | 24.7 | -6.6 |
| 164 | L_25 | Left Area 25 | -2.5 | 24.7 | -6.6 |
| 165 | L_s32 | Left Area s32 | -6 | 38.7 | -11.4 |
| 165 | L_s32 | Left Area s32 | -8.2 | 38.6 | -13.7 |
| 166 | L_pOFC | Left posterior OFC Complex | -11.4 | 24.2 | -16 |
| 167 | L_PoI1 | Left Area Posterior Insular 1 | -41.4 | -21.7 | 9.3 |
| 167 | L_PoI1 | Left Area Posterior Insular 1 | -43.3 | -13.6 | -5.7 |
| 167 | L_PoI1 | Left Area Posterior Insular 1 | -42.4 | -14.1 | -2 |
| 168 | L_Ig | Left Insular Granular Complex | -35 | -17.9 | 18.9 |
| 168 | L_Ig | Left Insular Granular Complex | -35.8 | -15.7 | 13.5 |
| 169 | L_FOP5 | Left Area Frontal Opercular 5 | -39.7 | 35.3 | 1.8 |
| 170 | L_p10p | Left Area posterior 10p | -21.1 | 65.6 | 14.5 |
| 170 | L_p10p | Left Area posterior 10p | -15.4 | 64.4 | 7.5 |
| 171 | L_p47r | Left Area posterior 47r | -38.8 | 48.7 | 10 |
| 171 | L_p47r | Left Area posterior 47r | -39.8 | 46.4 | 5.5 |
| 171 | L_p47r | Left Area posterior 47r | -38.7 | 46.4 | 5.5 |
| 172 | L_TGv | Left Area TG Ventral | -26.2 | -2.8 | -40.9 |
| 172 | L_TGv | Left Area TG Ventral | -37.8 | 4.3 | -43.7 |
| 173 | L_MBelt | Left Medial Belt Complex | -50.8 | -16.6 | -4.2 |
| 173 | L_MBelt | Left Medial Belt Complex | -50.7 | -10.7 | 5.9 |
| 173 | L_MBelt | Left Medial Belt Complex | -37.9 | -23.5 | 10.7 |
| 173 | L_MBelt | Left Medial Belt Complex | -45.8 | -12.8 | 5 |
| 175 | L_A4 | Left Auditory 4 Complex | -67.6 | -14.6 | 0.7 |
| 175 | L_A4 | Left Auditory 4 Complex | -62 | -41.4 | 19.3 |
| 175 | L_A4 | Left Auditory 4 Complex | -66.2 | -41.8 | 18.1 |
| 175 | L_A4 | Left Auditory 4 Complex | -68.1 | -24.2 | 3.6 |
| 176 | L_STSva | Left Area STSv anterior | -51.3 | -14.6 | -12.6 |
| 177 | L_TE1m | Left Area TE1 Middle | -59.7 | -35.5 | -21 |
| 177 | L_TE1m | Left Area TE1 Middle | -55.7 | -44.1 | -8.1 |
| 177 | L_TE1m | Left Area TE1 Middle | -51 | -30.2 | -19.4 |
| 178 | L_PI | Left Para-Insular Area | -48.5 | -8.3 | -10.2 |
| 178 | L_PI | Left Para-Insular Area | -47.4 | -8.2 | -10.1 |
| 179 | L_a32pr | Left Area anterior 32 prime | 0 | 31.1 | 27.4 |
| 179 | L_a32pr | Left Area anterior 32 prime | -6.4 | 27.6 | 18.9 |
| 179 | L_a32pr | Left Area anterior 32 prime | -8.5 | 33.5 | 29.2 |
| 180 | L_p24 | Left Area posterior 24 | -8.4 | 33.3 | 24.5 |
| 181 | R_V1 | Right Primary Visual Cortex | 7.3 | -98 | 8 |
| 181 | R_V1 | Right Primary Visual Cortex | 0.1 | -95.4 | 0.8 |
| 181 | R_V1 | Right Primary Visual Cortex | 13.2 | -76.1 | 15.6 |
| 181 | R_V1 | Right Primary Visual Cortex | 0 | -91.8 | 8.7 |
| 181 | R_V1 | Right Primary Visual Cortex | 10.9 | -76.2 | 13.2 |
| 181 | R_V1 | Right Primary Visual Cortex | 14.1 | -61.9 | 12 |
| 182 | R_MST | Right Medial Superior Temporal Area | 39.6 | -73.2 | 4.1 |
| 182 | R_MST | Right Medial Superior Temporal Area | 45.6 | -65 | 1.7 |
| 182 | R_MST | Right Medial Superior Temporal Area | 46.8 | -65.1 | -0.6 |
| 184 | R_V2 | Right Second Visual Area | 19.1 | -87.1 | -14 |
| 184 | R_V2 | Right Second Visual Area | 12.9 | -83.8 | -13.6 |
| 184 | R_V2 | Right Second Visual Area | 5.2 | -55.4 | -1.2 |
| 184 | R_V2 | Right Second Visual Area | 7 | -65.3 | 3 |
| 184 | R_V2 | Right Second Visual Area | 18.1 | -95.4 | -8.6 |
| 184 | R_V2 | Right Second Visual Area | 15.8 | -95.4 | -7.4 |
| 184 | R_V2 | Right Second Visual Area | 9.7 | -64.5 | 2.7 |
| 185 | R_V3 | Right Third Visual Area | 10 | -78.1 | -10.2 |
| 185 | R_V3 | Right Third Visual Area | 12.2 | -92.4 | 17.1 |
| 186 | R_V4 | Right Fourth Visual Area | 35.8 | -92.2 | 11.2 |
| 186 | R_V4 | Right Fourth Visual Area | 18.9 | -78.8 | -4.2 |
| 186 | R_V4 | Right Fourth Visual Area | 25.6 | -91.8 | 30 |
| 187 | R_V8 | Right Eighth Visual Area | 30.2 | -79 | -14.8 |
| 188 | R_4 | Right Primary Motor Cortex | 5.2 | -13.9 | 72.9 |
| 188 | R_4 | Right Primary Motor Cortex | 30.4 | -20.3 | 51.2 |
| 188 | R_4 | Right Primary Motor Cortex | 9.4 | -15.1 | 76.6 |
| 188 | R_4 | Right Primary Motor Cortex | 32.3 | -27.3 | 53.7 |
| 188 | R_4 | Right Primary Motor Cortex | 8 | -28.8 | 74.7 |
| 188 | R_4 | Right Primary Motor Cortex | 1.7 | -26.8 | 66.3 |
| 188 | R_4 | Right Primary Motor Cortex | 2.8 | -26.7 | 66.3 |
| 188 | R_4 | Right Primary Motor Cortex | 5.3 | -14.2 | 69 |
| 188 | R_4 | Right Primary Motor Cortex | 2 | -20.9 | 66 |
| 188 | R_4 | Right Primary Motor Cortex | 11 | -24 | 67.5 |
| 189 | R_3b | Right Primary Sensory Cortex | 48.5 | -11.4 | 42.1 |
| 189 | R_3b | Right Primary Sensory Cortex | 36.5 | -27.2 | 53.7 |
| 189 | R_3b | Right Primary Sensory Cortex | 12.1 | -31.5 | 68.5 |
| 189 | R_3b | Right Primary Sensory Cortex | 49.6 | -11.4 | 42.1 |
| 189 | R_3b | Right Primary Sensory Cortex | 13.2 | -25 | 68.8 |
| 189 | R_3b | Right Primary Sensory Cortex | 31.3 | -28.2 | 63.2 |
| 189 | R_3b | Right Primary Sensory Cortex | 61.5 | -4.7 | 30.5 |
| 189 | R_3b | Right Primary Sensory Cortex | 49.2 | -15.4 | 45.6 |
| 190 | R_FEF | Right Frontal Eye Fields | 44.5 | -4.4 | 56.4 |
| 190 | R_FEF | Right Frontal Eye Fields | 42.2 | -0.1 | 46.5 |
| 190 | R_FEF | Right Frontal Eye Fields | 43.4 | -1.3 | 44.3 |
| 190 | R_FEF | Right Frontal Eye Fields | 51.2 | -4.2 | 50.5 |
| 190 | R_FEF | Right Frontal Eye Fields | 45.8 | -14.4 | 43.1 |
| 192 | R_55b | Right Area 55b | 37.1 | -5.4 | 53 |
| 192 | R_55b | Right Area 55b | 50.8 | -5.7 | 51.2 |
| 192 | R_55b | Right Area 55b | 47.5 | -9.5 | 45.1 |
| 194 | R_RSC | Right RetroSplenial Complex | 8.7 | -43.8 | 25.9 |
| 194 | R_RSC | Right RetroSplenial Complex | 5.4 | -48.1 | 14.3 |
| 194 | R_RSC | Right RetroSplenial Complex | 2.5 | -38.7 | 26.3 |
| 196 | R_V7 | Right Seventh Visual Area | 30.9 | -74.1 | 33.2 |
| 198 | R_FFC | Right Fusiform Face Complex | 40.2 | -48.8 | -16.2 |
| 198 | R_FFC | Right Fusiform Face Complex | 33.2 | -74 | -2.6 |
| 198 | R_FFC | Right Fusiform Face Complex | 36.5 | -67 | -14 |
| 199 | R_V3B | Right Area V3B | 29.1 | -68.2 | 32.1 |
| 200 | R_LO1 | Right Area Lateral Occipital1 | 43.6 | -79.5 | 13.2 |
| 200 | R_LO1 | Right Area Lateral Occipital1 | 37 | -93.4 | 9 |
| 203 | R_MT | Right Middle Temporal Area | 37.5 | -71.3 | 7.1 |
| 204 | R_A1 | Right Primary Auditory Cortex | 42.9 | -23.9 | 11.8 |
| 205 | R_PSL | Right PeriSylvian Language Area | 65.8 | -36.5 | 24.8 |
| 205 | R_PSL | Right PeriSylvian Language Area | 61.6 | -37.9 | 23.8 |
| 207 | R_PCV | Right PreCuneus Visual Area | 5.2 | -56.3 | 53.9 |
| 208 | R_STV | Right Superior Temporal Visual Area | 57.7 | -52.5 | 10 |
| 208 | R_STV | Right Superior Temporal Visual Area | 58.8 | -51.3 | 13.5 |
| 209 | R_7Pm | Right Medial Area 7P | 11.2 | -66.6 | 56 |
| 210 | R_7m | Right Area 7m | 3.8 | -67.5 | 44.8 |
| 211 | R_POS1 | Right Parieto-Occipital Sulcus Area 1 | 7 | -67.6 | 23.6 |
| 211 | R_POS1 | Right Parieto-Occipital Sulcus Area 1 | 8.2 | -63 | 11.5 |
| 211 | R_POS1 | Right Parieto-Occipital Sulcus Area 1 | 9.5 | -51.5 | 9.4 |
| 211 | R_POS1 | Right Parieto-Occipital Sulcus Area 1 | 10.6 | -57.1 | 26.8 |
| 212 | R_23d | Right Area 23d | 2.3 | -20.9 | 33 |
| 215 | R_31pv | Right Area 31p ventral | 8.1 | -38.3 | 33.4 |
| 218 | R_23c | Right Area 23c | 6.3 | -13.9 | 31.5 |
| 218 | R_23c | Right Area 23c | 6 | -22.7 | 39.8 |
| 218 | R_23c | Right Area 23c | 13.6 | -38 | 41.6 |
| 218 | R_23c | Right Area 23c | 12.5 | -38.1 | 39.3 |
| 218 | R_23c | Right Area 23c | 10.3 | -38.2 | 35.8 |
| 218 | R_23c | Right Area 23c | 11.2 | -19.6 | 35.3 |
| 219 | R_5L | Right Area 5L | 6.4 | -50.4 | 76.4 |
| 219 | R_5L | Right Area 5L | 8.6 | -46.4 | 74.6 |
| 219 | R_5L | Right Area 5L | 9.7 | -46.4 | 74.6 |
| 220 | R_24dd | Right Dorsal Area 24d | 5.9 | -27.7 | 49.7 |
| 220 | R_24dd | Right Dorsal Area 24d | 9.5 | -9.8 | 51 |
| 220 | R_24dd | Right Dorsal Area 24d | 13.5 | -20.7 | 38.5 |
| 220 | R_24dd | Right Dorsal Area 24d | 0.5 | -31.2 | 53.6 |
| 220 | R_24dd | Right Dorsal Area 24d | 13.4 | -20.6 | 37.7 |
| 221 | R_24dv | Right Ventral Area 24d | 8.7 | -7.4 | 41.2 |
| 221 | R_24dv | Right Ventral Area 24d | 8.8 | -6.5 | 36.4 |
| 225 | R_7Am | Right Medial Area 7A | 2.4 | -53.8 | 65.5 |
| 227 | R_7PC | Right Area 7PC | 33.8 | -53.2 | 66.7 |
| 230 | R_MIP | Right Medial IntraParietal Area | 32.3 | -67.6 | 63.4 |
| 231 | R_1 | Right Area 1 | 64.6 | -3.8 | 19.7 |
| 231 | R_1 | Right Area 1 | 51.5 | -19.2 | 58.2 |
| 231 | R_1 | Right Area 1 | 55.7 | -19.9 | 55.1 |
| 231 | R_1 | Right Area 1 | 40.8 | -24 | 65.6 |
| 231 | R_1 | Right Area 1 | 41.9 | -24 | 65.6 |
| 231 | R_1 | Right Area 1 | 49.2 | -24.9 | 61.5 |
| 231 | R_1 | Right Area 1 | 25.8 | -32.4 | 72.4 |
| 231 | R_1 | Right Area 1 | 61.5 | -17.3 | 47 |
| 231 | R_1 | Right Area 1 | 40.2 | -28.1 | 60.9 |
| 231 | R_1 | Right Area 1 | 66 | -4.7 | 29.3 |
| 231 | R_1 | Right Area 1 | 51.3 | -16.1 | 55.1 |
| 231 | R_1 | Right Area 1 | 39.1 | -27.9 | 67.9 |
| 232 | R_2 | Right Area 2 | 15.3 | -32.5 | 64.3 |
| 232 | R_2 | Right Area 2 | 40.7 | -29.6 | 48.7 |
| 233 | R_3a | Right Area 3a | 56.1 | -5 | 19.8 |
| 234 | R_6d | Right Dorsal area 6 | 37.9 | -16.2 | 66.3 |
| 234 | R_6d | Right Dorsal area 6 | 32.5 | -21.3 | 68.2 |
| 234 | R_6d | Right Dorsal area 6 | 37.8 | -12.6 | 67.7 |
| 235 | R_6mp | Right Area 6mp | 12.7 | -11.7 | 72.7 |
| 235 | R_6mp | Right Area 6mp | 23 | -18 | 73 |
| 236 | R_6v | Right Ventral Area 6 | 57.2 | -2.8 | 23.9 |
| 236 | R_6v | Right Ventral Area 6 | 56.3 | 1.7 | 28.6 |
| 236 | R_6v | Right Ventral Area 6 | 62.5 | -0.3 | 29.1 |
| 237 | R_p24pr | Right Area Posterior 24 prime | 1.1 | -10.4 | 28.4 |
| 237 | R_p24pr | Right Area Posterior 24 prime | 7.7 | -6.5 | 36.4 |
| 237 | R_p24pr | Right Area Posterior 24 prime | 8.6 | 2.4 | 40.3 |
| 238 | R_33pr | Right Area 33 prime | 2.7 | 10.6 | 24.5 |
| 239 | R_a24pr | Right Anterior 24 prime | 7.9 | 10.8 | 29.1 |
| 240 | R_p32pr | Right Area p32 prime | 8.6 | 3.6 | 44.9 |
| 241 | R_a24 | Right Area a24 | 4 | 40.2 | -4.4 |
| 242 | R_d32 | Right Area dorsal 32 | 7.8 | 45.2 | 30.5 |
| 242 | R_d32 | Right Area dorsal 32 | 10.9 | 43.3 | 23.1 |
| 242 | R_d32 | Right Area dorsal 32 | 8.8 | 45.3 | 30.6 |
| 242 | R_d32 | Right Area dorsal 32 | 6.7 | 45.2 | 30.5 |
| 242 | R_d32 | Right Area dorsal 32 | 7.2 | 42.3 | 22.6 |
| 242 | R_d32 | Right Area dorsal 32 | 9.4 | 42.3 | 23.7 |
| 243 | R_8BM | Right Area 8BM | 7 | 14.2 | 46.4 |
| 243 | R_8BM | Right Area 8BM | 1.7 | 17.8 | 47.2 |
| 244 | R_p32 | Right Area p32 | 9.9 | 44.8 | -8.6 |
| 244 | R_p32 | Right Area p32 | 11.7 | 52.6 | 1.6 |
| 245 | R_10r | Right Area 10r | 9.5 | 53.3 | -7.9 |
| 246 | R_47m | Right Area 47m | 22 | 40.3 | -10.3 |
| 247 | R_8Av | Right Area 8Av | 35 | -4.6 | 56.2 |
| 247 | R_8Av | Right Area 8Av | 43.8 | 8.8 | 43.8 |
| 247 | R_8Av | Right Area 8Av | 26.3 | 16.3 | 54.5 |
| 247 | R_8Av | Right Area 8Av | 31.9 | 15.2 | 52.2 |
| 248 | R_8Ad | Right Area 8Ad | 16.9 | 29.9 | 54.9 |
| 248 | R_8Ad | Right Area 8Ad | 17.9 | 29.9 | 55 |
| 249 | R_9m | Right Area 9 Middle | 5.9 | 53.7 | 8.7 |
| 249 | R_9m | Right Area 9 Middle | 12.7 | 52.8 | 8.6 |
| 250 | R_8BL | Right Area 8B Lateral | 4.2 | 29.3 | 53.6 |
| 251 | R_9p | Right Area 9 Posterior | 20.3 | 40.1 | 35.2 |
| 251 | R_9p | Right Area 9 Posterior | 9.1 | 53.9 | 41.6 |
| 252 | R_10d | Right Area 10d | 9.3 | 63.1 | 2.5 |
| 253 | R_8C | Right Area 8C | 40.6 | 9.2 | 41.4 |
| 253 | R_8C | Right Area 8C | 28.2 | 19.1 | 38.2 |
| 254 | R_44 | Right Area 44 | 60.8 | 15.9 | 11.5 |
| 254 | R_44 | Right Area 44 | 44.9 | 13 | 1.6 |
| 255 | R_45 | Right Area 45 | 55.8 | 23.8 | -0.2 |
| 255 | R_45 | Right Area 45 | 53.6 | 23.1 | -2.4 |
| 256 | R_47l | Right Area 47l (47 lateral) | 37 | 31.2 | -12.1 |
| 256 | R_47l | Right Area 47l (47 lateral) | 43.3 | 40.6 | -12.6 |
| 257 | R_a47r | Right Area anterior 47r | 36.5 | 53.1 | -8.4 |
| 257 | R_a47r | Right Area anterior 47r | 29.2 | 56.5 | 0.9 |
| 257 | R_a47r | Right Area anterior 47r | 28.2 | 59.8 | 9.2 |
| 257 | R_a47r | Right Area anterior 47r | 30.2 | 56.5 | 0.9 |
| 257 | R_a47r | Right Area anterior 47r | 28.1 | 56.5 | 0.9 |
| 257 | R_a47r | Right Area anterior 47r | 29.2 | 56.8 | 2 |
| 257 | R_a47r | Right Area anterior 47r | 37.5 | 53.1 | -8.4 |
| 257 | R_a47r | Right Area anterior 47r | 29.2 | 59.8 | 9.3 |
| 257 | R_a47r | Right Area anterior 47r | 33 | 53.7 | -9.1 |
| 258 | R_6r | Right Rostral Area 6 | 55.8 | 13 | 5.3 |
| 261 | R_IFSp | Right Area IFSp | 48.9 | 13.7 | 31.2 |
| 261 | R_IFSp | Right Area IFSp | 50 | 13.7 | 31.2 |
| 261 | R_IFSp | Right Area IFSp | 39.9 | 26.3 | 20.5 |
| 262 | R_IFSa | Right Area IFSa | 44.2 | 40.4 | 12.2 |
| 263 | R_p9-46v | Right Area posterior 9-46v | 52.9 | 34.1 | 27 |
| 263 | R_p9-46v | Right Area posterior 9-46v | 49.4 | 23.8 | 25.4 |
| 263 | R_p9-46v | Right Area posterior 9-46v | 40.9 | 27.6 | 24 |
| 263 | R_p9-46v | Right Area posterior 9-46v | 42.1 | 27.6 | 24 |
| 264 | R_46 | Right Area 46 | 28.5 | 31.5 | 43.7 |
| 264 | R_46 | Right Area 46 | 25.3 | 30.6 | 40.4 |
| 264 | R_46 | Right Area 46 | 28.7 | 39.3 | 40 |
| 264 | R_46 | Right Area 46 | 26.4 | 30.6 | 40.4 |
| 264 | R_46 | Right Area 46 | 18.4 | 27.9 | 46.3 |
| 264 | R_46 | Right Area 46 | 21.8 | 27.8 | 40.4 |
| 264 | R_46 | Right Area 46 | 26.1 | 42.9 | 32 |
| 265 | R_a9-46v | Right Area anterior 9-46v | 33.4 | 56.2 | 7.8 |
| 265 | R_a9-46v | Right Area anterior 9-46v | 39.6 | 54.5 | 10.9 |
| 266 | R_9-46d | Right Area 9-46d | 24.9 | 53.4 | 27.2 |
| 266 | R_9-46d | Right Area 9-46d | 21.3 | 38.2 | 32.2 |
| 266 | R_9-46d | Right Area 9-46d | 22.7 | 52.5 | 23.9 |
| 266 | R_9-46d | Right Area 9-46d | 22.7 | 42.9 | 34.3 |
| 267 | R_9a | Right Area 9 anterior | 12.5 | 53.9 | 41.6 |
| 268 | R_10v | Right Area 10v | 3.7 | 51 | -22.2 |
| 268 | R_10v | Right Area 10v | 5 | 61.1 | -5 |
| 268 | R_10v | Right Area 10v | 7 | 67.7 | -3.4 |
| 268 | R_10v | Right Area 10v | 3.7 | 67.4 | -9.2 |
| 269 | R_a10p | Right Area anterior 10p | 20.5 | 67.9 | -3.3 |
| 269 | R_a10p | Right Area anterior 10p | 21.6 | 67.8 | -6.9 |
| 269 | R_a10p | Right Area anterior 10p | 25 | 67.8 | -6.9 |
| 270 | R_10pp | Right Polar 10p | 10.5 | 67.1 | -21 |
| 271 | R_11l | Right Area 11l | 21.6 | 51.3 | -14 |
| 271 | R_11l | Right Area 11l | 25.2 | 54.6 | -9.2 |
| 272 | R_13l | Right Area 13l | 21.8 | 16.8 | -19.9 |
| 273 | R_OFC | Right Orbital Frontal Complex | 8.7 | 40.1 | -22.3 |
| 273 | R_OFC | Right Orbital Frontal Complex | 11 | 49.1 | -21.7 |
| 273 | R_OFC | Right Orbital Frontal Complex | 10.2 | 18.7 | -20.5 |
| 273 | R_OFC | Right Orbital Frontal Complex | 9.7 | 40.1 | -22.3 |
| 273 | R_OFC | Right Orbital Frontal Complex | 11.3 | 18.7 | -20.5 |
| 273 | R_OFC | Right Orbital Frontal Complex | 9.5 | 31.2 | -22.9 |
| 273 | R_OFC | Right Orbital Frontal Complex | 13.7 | 31.6 | -21.7 |
| 273 | R_OFC | Right Orbital Frontal Complex | 9.6 | 52.9 | -19.7 |
| 273 | R_OFC | Right Orbital Frontal Complex | 11.8 | 53 | -19.7 |
| 273 | R_OFC | Right Orbital Frontal Complex | 7.5 | 38.6 | -20.8 |
| 273 | R_OFC | Right Orbital Frontal Complex | 9.8 | 38.6 | -22 |
| 273 | R_OFC | Right Orbital Frontal Complex | 6.4 | 38.5 | -24.3 |
| 274 | R_47s | Right Area 47s | 21.1 | 24.7 | -19.5 |
| 276 | R_6a | Right Area 6 anterior | 24.4 | -5.8 | 48.3 |
| 276 | R_6a | Right Area 6 anterior | 27.6 | -6.5 | 49.7 |
| 276 | R_6a | Right Area 6 anterior | 28.6 | -5.6 | 52.9 |
| 277 | R_i6-8 | Right Inferior 6-8 Transitional Area | 21.7 | 13.6 | 51.4 |
| 277 | R_i6-8 | Right Inferior 6-8 Transitional Area | 35.4 | 3.4 | 55.7 |
| 278 | R_s6-8 | Right Superior 6-8 Transitional Area | 32 | 4.5 | 57.9 |
| 278 | R_s6-8 | Right Superior 6-8 Transitional Area | 33.1 | 4.6 | 60.3 |
| 279 | R_43 | Right Area 43 | 44.3 | -9.7 | 6.9 |
| 279 | R_43 | Right Area 43 | 62.7 | 6.6 | 17.8 |
| 279 | R_43 | Right Area 43 | 43.8 | -6 | 2.2 |
| 280 | R_OP4 | Right Area OP4/PV | 65 | -4.2 | 10.4 |
| 284 | R_RI | Right RetroInsular Cortex | 40.6 | -33.9 | 18.9 |
| 286 | R_PoI2_ROI | Right Posterior Insular Area 2 | 38 | -11.6 | 0.4 |
| 287 | R_TA2 | Right Area TA2 | 50.7 | -10.4 | 3.8 |
| 287 | R_TA2 | Right Area TA2 | 52 | -0.7 | 3.2 |
| 287 | R_TA2 | Right Area TA2 | 47 | 10.3 | -4.6 |
| 287 | R_TA2 | Right Area TA2 | 45 | -6.3 | -9.5 |
| 289 | R_MI | Right Middle Insular Area | 37.6 | 13.9 | 1.2 |
| 290 | R_Pir | Right Pirform Cortex | 31.4 | 8.5 | -16.8 |
| 291 | R_AVI | Right Anterior Ventral Insular Area | 33.3 | 25.7 | 5.2 |
| 293 | R_FOP1 | Right Frontal Opercular Area 1 | 42.5 | -0.1 | 6.2 |
| 294 | R_FOP3 | Right Frontal Opercular Area 3 | 35.2 | 4.7 | 16.3 |
| 294 | R_FOP3 | Right Frontal Opercular Area 3 | 35.7 | 9.5 | 9.1 |
| 296 | R_PFt | Right Area PFt | 53.4 | -28.4 | 52.1 |
| 297 | R_AIP | Right Anterior IntraParietal Area | 39.4 | -38.1 | 58.1 |
| 297 | R_AIP | Right Anterior IntraParietal Area | 39.3 | -41.2 | 46.3 |
| 297 | R_AIP | Right Anterior IntraParietal Area | 41.4 | -42.7 | 44.4 |
| 297 | R_AIP | Right Anterior IntraParietal Area | 50.4 | -30.2 | 58.7 |
| 297 | R_AIP | Right Anterior IntraParietal Area | 37.2 | -46.1 | 48.3 |
| 297 | R_AIP | Right Anterior IntraParietal Area | 38.3 | -44.9 | 51.8 |
| 298 | R_EC | Right Entorhinal Cortex | 19.9 | -15.6 | -26.2 |
| 298 | R_EC | Right Entorhinal Cortex | 13.8 | -11.7 | -24.5 |
| 299 | R_PreS | Right PreSubiculum | 20.7 | -25.7 | -18.7 |
| 301 | R_ProS | Right ProStriate Area | 11.8 | -47 | 1.2 |
| 302 | R_PeEc | Right Perirhinal Ectorhinal Cortex | 34.6 | -17.2 | -33.4 |
| 302 | R_PeEc | Right Perirhinal Ectorhinal Cortex | 30.5 | -15.9 | -28.1 |
| 302 | R_PeEc | Right Perirhinal Ectorhinal Cortex | 26.2 | -15.7 | -27.2 |
| 302 | R_PeEc | Right Perirhinal Ectorhinal Cortex | 24 | -19.3 | -26.1 |
| 302 | R_PeEc | Right Perirhinal Ectorhinal Cortex | 21.7 | -10.7 | -31.6 |
| 302 | R_PeEc | Right Perirhinal Ectorhinal Cortex | 30.7 | -19.2 | -26.1 |
| 304 | R_PBelt | Right ParaBelt Complex | 58.8 | -22.9 | 14.2 |
| 304 | R_PBelt | Right ParaBelt Complex | 58.1 | -9.7 | 6.1 |
| 304 | R_PBelt | Right ParaBelt Complex | 53.9 | -7.1 | -5.9 |
| 305 | R_A5 | Right Auditory 5 Complex | 60.7 | -28.6 | 0.7 |
| 305 | R_A5 | Right Auditory 5 Complex | 60.6 | 7 | -5.9 |
| 306 | R_PHA1 | Right ParaHippocampal Area 1 | 17.2 | -38.8 | -9.6 |
| 307 | R_PHA3 | Right ParaHippocampal Area 3 | 32.1 | -34.6 | -15.6 |
| 307 | R_PHA3 | Right ParaHippocampal Area 3 | 27.6 | -45.7 | -15.9 |
| 307 | R_PHA3 | Right ParaHippocampal Area 3 | 34.1 | -19.1 | -26.1 |
| 307 | R_PHA3 | Right ParaHippocampal Area 3 | 30.8 | -36.2 | -12.7 |
| 308 | R_STSda | Right Area STSd anterior | 47.3 | -16.5 | -6.3 |
| 308 | R_STSda | Right Area STSd anterior | 57.1 | -4.9 | -4.9 |
| 308 | R_STSda | Right Area STSd anterior | 48.5 | -18.2 | -6.1 |
| 308 | R_STSda | Right Area STSd anterior | 51.8 | -6.4 | -16.6 |
| 309 | R_STSdp | Right Area STSd posterior | 48.6 | -36.4 | 6.2 |
| 309 | R_STSdp | Right Area STSd posterior | 46.4 | -36.4 | 7.4 |
| 310 | R_STSvp | Right Area STSv posterior | 52.2 | -31.5 | -1.2 |
| 310 | R_STSvp | Right Area STSv posterior | 49.1 | -31.6 | -1.2 |
| 310 | R_STSvp | Right Area STSv posterior | 63.6 | -42.2 | -1.1 |
| 311 | R_TGd | Right Area TG dorsal | 24.9 | 12.6 | -28 |
| 311 | R_TGd | Right Area TG dorsal | 41.8 | 15.6 | -13.6 |
| 311 | R_TGd | Right Area TG dorsal | 51.1 | 7 | -34.2 |
| 311 | R_TGd | Right Area TG dorsal | 52.2 | 7.1 | -34.2 |
| 311 | R_TGd | Right Area TG dorsal | 41.1 | 27.9 | -29.4 |
| 311 | R_TGd | Right Area TG dorsal | 26.8 | 22.3 | -28.7 |
| 312 | R_TE1a | Right Area TE1 anterior | 55.9 | -12 | -19.3 |
| 312 | R_TE1a | Right Area TE1 anterior | 56.3 | 3.3 | -15.4 |
| 312 | R_TE1a | Right Area TE1 anterior | 48.8 | -1.5 | -24.8 |
| 312 | R_TE1a | Right Area TE1 anterior | 51.9 | -1.4 | -24.7 |
| 312 | R_TE1a | Right Area TE1 anterior | 50.7 | -6.8 | -27.2 |
| 312 | R_TE1a | Right Area TE1 anterior | 49.6 | -7 | -30.7 |
| 312 | R_TE1a | Right Area TE1 anterior | 65.4 | -18.3 | -17.8 |
| 312 | R_TE1a | Right Area TE1 anterior | 51.8 | -6.8 | -27.2 |
| 312 | R_TE1a | Right Area TE1 anterior | 66.5 | -18.3 | -17.8 |
| 313 | R_TE1p | Right Area TE1 posterior | 53.2 | -35 | -10.6 |
| 313 | R_TE1p | Right Area TE1 posterior | 53.2 | -35.9 | -13.8 |
| 313 | R_TE1p | Right Area TE1 posterior | 46.5 | -50 | -16.9 |
| 313 | R_TE1p | Right Area TE1 posterior | 59.1 | -50.3 | -18.8 |
| 313 | R_TE1p | Right Area TE1 posterior | 58.2 | -44.8 | -6.4 |
| 313 | R_TE1p | Right Area TE1 posterior | 56.1 | -46.5 | -8.4 |
| 313 | R_TE1p | Right Area TE1 posterior | 54.9 | -51.5 | -10.8 |
| 313 | R_TE1p | Right Area TE1 posterior | 66.6 | -46.8 | 2.1 |
| 313 | R_TE1p | Right Area TE1 posterior | 52.8 | -51 | -8.7 |
| 313 | R_TE1p | Right Area TE1 posterior | 58 | -54.5 | -15.7 |
| 313 | R_TE1p | Right Area TE1 posterior | 46.8 | -54.7 | -16.9 |
| 313 | R_TE1p | Right Area TE1 posterior | 47.9 | -54.7 | -16.9 |
| 313 | R_TE1p | Right Area TE1 posterior | 60.2 | -54.4 | -14.6 |
| 314 | R_TE2a | Right TE2a | 61.6 | -37.7 | -21.1 |
| 314 | R_TE2a | Right TE2a | 51.6 | -13 | -22.6 |
| 314 | R_TE2a | Right TE2a | 55.9 | -12.9 | -22.5 |
| 314 | R_TE2a | Right TE2a | 62.2 | -13.6 | -25.6 |
| 314 | R_TE2a | Right TE2a | 51.7 | -12.1 | -19.4 |
| 314 | R_TE2a | Right TE2a | 52.7 | -12.1 | -19.4 |
| 314 | R_TE2a | Right TE2a | 42.8 | 5.9 | -35.4 |
| 314 | R_TE2a | Right TE2a | 41.6 | 6 | -33.1 |
| 314 | R_TE2a | Right TE2a | 64.5 | -37 | -19.7 |
| 314 | R_TE2a | Right TE2a | 47.5 | -7.3 | -38.9 |
| 314 | R_TE2a | Right TE2a | 48.6 | -7.3 | -38.9 |
| 314 | R_TE2a | Right TE2a | 47.4 | -7 | -29.5 |
| 315 | R_TF | Right Area TF | 36.2 | -39.1 | -24.8 |
| 315 | R_TF | Right Area TF | 43.9 | -26.6 | -23.6 |
| 315 | R_TF | Right Area TF | 38.6 | -27 | -24.8 |
| 315 | R_TF | Right Area TF | 42.1 | -14.5 | -23.6 |
| 315 | R_TF | Right Area TF | 40 | -15.7 | -28 |
| 315 | R_TF | Right Area TF | 36 | -5.2 | -37.9 |
| 315 | R_TF | Right Area TF | 41.9 | -8.4 | -36.5 |
| 315 | R_TF | Right Area TF | 43 | -19 | -26.1 |
| 315 | R_TF | Right Area TF | 39.7 | -19.1 | -28.4 |
| 315 | R_TF | Right Area TF | 37.4 | -10.6 | -35.1 |
| 316 | R_TE2p | Right Area TE2 posterior | 47.8 | -38 | -21.4 |
| 316 | R_TE2p | Right Area TE2 posterior | 43.6 | -38.1 | -21.4 |
| 316 | R_TE2p | Right Area TE2 posterior | 45.7 | -37.5 | -19.3 |
| 316 | R_TE2p | Right Area TE2 posterior | 49.2 | -27.6 | -27.8 |
| 316 | R_TE2p | Right Area TE2 posterior | 44.4 | -48.9 | -12.7 |
| 316 | R_TE2p | Right Area TE2 posterior | 42.1 | -55.3 | -16 |
| 316 | R_TE2p | Right Area TE2 posterior | 44.3 | -37.2 | -16.2 |
| 316 | R_TE2p | Right Area TE2 posterior | 40.9 | -37.3 | -17.3 |
| 317 | R_PHT | Right Area PHT | 55.7 | -62.3 | -9.9 |
| 318 | R_PH | Right Area PH | 43.5 | -65.3 | -6.5 |
| 318 | R_PH | Right Area PH | 42.4 | -65.5 | -10.1 |
| 319 | R_TPOJ1 | Right Area TemporoParietoOccipital Junction 1 | 65.7 | -41.8 | 8.9 |
| 319 | R_TPOJ1 | Right Area TemporoParietoOccipital Junction 1 | 47.7 | -42.9 | 9.8 |
| 319 | R_TPOJ1 | Right Area TemporoParietoOccipital Junction 1 | 61 | -36.4 | 0.3 |
| 320 | R_TPOJ2 | Right Area TemporoParietoOccipital Junction 1 | 56.7 | -63.3 | 15.7 |
| 322 | R_DVT | Right Dorsal Transitional Visual Area | 25.3 | -60.5 | 16.6 |
| 323 | R_PGp | Right Area PGp | 38.8 | -76.2 | 29.8 |
| 323 | R_PGp | Right Area PGp | 40 | -76.2 | 29.8 |
| 323 | R_PGp | Right Area PGp | 50 | -76.1 | 28.7 |
| 324 | R_IP2 | Right Area IntraParietal 2 | 44.6 | -41.3 | 45.4 |
| 324 | R_IP2 | Right Area IntraParietal 2 | 44.2 | -56.1 | 43.6 |
| 324 | R_IP2 | Right Area IntraParietal 2 | 45.3 | -56.1 | 43.6 |
| 324 | R_IP2 | Right Area IntraParietal 2 | 51.6 | -32.6 | 50.7 |
| 324 | R_IP2 | Right Area IntraParietal 2 | 51.6 | -32.7 | 48.4 |
| 324 | R_IP2 | Right Area IntraParietal 2 | 39.5 | -46.3 | 41.3 |
| 325 | R_IP1 | Right Area IntraParietal 1 | 36.6 | -65.7 | 45.2 |
| 325 | R_IP1 | Right Area IntraParietal 1 | 40.7 | -56.9 | 51.7 |
| 325 | R_IP1 | Right Area IntraParietal 1 | 26.3 | -72.5 | 49.5 |
| 326 | R_IP0 | Right Area IntraParietal 0 | 32 | -72.6 | 42.5 |
| 327 | R_PFop | Right Area PF opercular | 68.3 | -20.7 | 21.9 |
| 328 | R_PF | Right Area PF Complex | 62 | -22.8 | 47.9 |
| 328 | R_PF | Right Area PF Complex | 67 | -32 | 41.9 |
| 329 | R_PFm | Right Area PFm Complex | 49.6 | -52.2 | 20.6 |
| 329 | R_PFm | Right Area PFm Complex | 51.9 | -44.1 | 42.7 |
| 329 | R_PFm | Right Area PFm Complex | 55.7 | -61 | 32.5 |
| 329 | R_PFm | Right Area PFm Complex | 64.3 | -50.6 | 30 |
| 329 | R_PFm | Right Area PFm Complex | 50.9 | -60.6 | 34.4 |
| 329 | R_PFm | Right Area PFm Complex | 65.4 | -50.6 | 30 |
| 333 | R_VMV1 | Right VentroMedial Visual Area 1 | 21 | -53.9 | -8.9 |
| 334 | R_VMV3 | Right VentroMedial Visual Area 3 | 27.1 | -64.2 | -6.9 |
| 334 | R_VMV3 | Right VentroMedial Visual Area 3 | 29.9 | -52.5 | -8.9 |
| 334 | R_VMV3 | Right VentroMedial Visual Area 3 | 24.4 | -65.6 | -4.2 |
| 335 | R_PHA2 | Right ParaHippocampal Area 2 | 29.1 | -26.6 | -22.8 |
| 335 | R_PHA2 | Right ParaHippocampal Area 2 | 29.8 | -42.2 | -10.9 |
| 335 | R_PHA2 | Right ParaHippocampal Area 2 | 28.7 | -43.8 | -12.9 |
| 335 | R_PHA2 | Right ParaHippocampal Area 2 | 28.5 | -35.1 | -11.7 |
| 337 | R_FST | Right Area FST | 36.9 | -78.5 | -5.4 |
| 337 | R_FST | Right Area FST | 39.1 | -78.5 | -6.5 |
| 338 | R_V3CD | Right Area V3CD | 37.3 | -78.2 | 18.4 |
| 340 | R_VMV2 | Right VentroMedial Visual Area 2 | 26.5 | -52.5 | -6.6 |
| 342 | R_31a | Right Area 31a | 10.6 | -51.7 | 42 |
| 343 | R_VVC | Right Ventral Visual Complex | 19.7 | -66.1 | -13.4 |
| 343 | R_VVC | Right Ventral Visual Complex | 31.3 | -67.4 | -15.1 |
| 343 | R_VVC | Right Ventral Visual Complex | 30.2 | -67.7 | -16.2 |
| 343 | R_VVC | Right Ventral Visual Complex | 27.5 | -37.6 | -20.9 |
| 344 | R_25 | Right Area 25 | 2 | 24.8 | -5.4 |
| 345 | R_s32 | Right Area s32 | 6.4 | 32.3 | -14.2 |
| 345 | R_s32 | Right Area s32 | 5.2 | 38.8 | -13.7 |
| 345 | R_s32 | Right Area s32 | 6.3 | 38.8 | -13.7 |
| 346 | R_pOFC | Right posterior OFC Complex | 4.6 | 5.4 | -20.4 |
| 346 | R_pOFC | Right posterior OFC Complex | 15.5 | 19.3 | -18.3 |
| 346 | R_pOFC | Right posterior OFC Complex | 6.5 | 24.6 | -14.8 |
| 346 | R_pOFC | Right posterior OFC Complex | 14.3 | 24.8 | -11.2 |
| 347 | R_PoI1_ROI | Right Area Posterior Insular 1 | 30.2 | -25 | 8.3 |
| 347 | R_PoI1_ROI | Right Area Posterior Insular 1 | 36.1 | -18.1 | 2.1 |
| 349 | R_FOP5 | Right Area Frontal Opercular 5 | 32.6 | 26.2 | 6.6 |
| 349 | R_FOP5 | Right Area Frontal Opercular 5 | 35.5 | 25.9 | 8.8 |
| 349 | R_FOP5 | Right Area Frontal Opercular 5 | 36.6 | 25.9 | 8.8 |
| 350 | R_p10p | Right Area posterior 10p | 18.1 | 67.4 | 15.6 |
| 350 | R_p10p | Right Area posterior 10p | 21.5 | 67.4 | 14.4 |
| 351 | R_p47r | Right Area posterior 47r | 51.8 | 32.7 | -7.6 |
| 351 | R_p47r | Right Area posterior 47r | 47.7 | 40.9 | -6.7 |
| 351 | R_p47r | Right Area posterior 47r | 48.8 | 41 | -4.4 |
| 352 | R_TGv | Right Area TG Ventral | 46.5 | -5.8 | -40.9 |
| 352 | R_TGv | Right Area TG Ventral | 47.6 | -5.8 | -40.9 |
| 352 | R_TGv | Right Area TG Ventral | 32.9 | -4.7 | -35.8 |
| 352 | R_TGv | Right Area TG Ventral | 27.6 | -5.7 | -39.1 |
| 352 | R_TGv | Right Area TG Ventral | 27.6 | -4.3 | -33.8 |
| 352 | R_TGv | Right Area TG Ventral | 25.5 | -2.4 | -30.8 |
| 352 | R_TGv | Right Area TG Ventral | 24.9 | 5.3 | -44.8 |
| 353 | R_MBelt | Right Medial Belt Complex | 40.6 | -18.1 | 1 |
| 355 | R_A4 | Right Auditory 4 Complex | 69.3 | -24.9 | 5.9 |
| 355 | R_A4 | Right Auditory 4 Complex | 61.7 | -33.2 | 20.7 |
| 355 | R_A4 | Right Auditory 4 Complex | 65.7 | -1.7 | 2.6 |
| 355 | R_A4 | Right Auditory 4 Complex | 62.5 | -1.8 | 2.5 |
| 355 | R_A4 | Right Auditory 4 Complex | 59.6 | -34.1 | 17.5 |
| 356 | R_STSva | Right Area STSv anterior | 48.3 | -18.3 | -9.3 |
| 356 | R_STSva | Right Area STSv anterior | 60.2 | -7 | -8.9 |
| 356 | R_STSva | Right Area STSv anterior | 54 | -6.5 | -20.1 |
| 356 | R_STSva | Right Area STSv anterior | 50.7 | -18.2 | -8.4 |
| 356 | R_STSva | Right Area STSv anterior | 57.4 | -6.4 | -17.7 |
| 357 | R_TE1m | Right Area TE1 Middle | 57.8 | -20.8 | -15.4 |
| 357 | R_TE1m | Right Area TE1 Middle | 57.7 | -22.7 | -18.4 |
| 357 | R_TE1m | Right Area TE1 Middle | 55.6 | -22.5 | -17.3 |
| 357 | R_TE1m | Right Area TE1 Middle | 54.6 | -20.6 | -14.4 |
| 358 | R_PI | Right Para-Insular Area | 40 | -15 | -4.5 |
| 358 | R_PI | Right Para-Insular Area | 45.5 | -7.1 | -8.1 |
| 358 | R_PI | Right Para-Insular Area | 39.3 | 8.6 | -18 |
| 358 | R_PI | Right Para-Insular Area | 41.7 | -6.5 | -14.2 |
| 359 | R_a32pr | Right Area anterior 32 prime | 3.3 | 34 | 25.8 |
| 359 | R_a32pr | Right Area anterior 32 prime | 3.3 | 34.9 | 29 |
| 359 | R_a32pr | Right Area anterior 32 prime | 6.1 | 27.2 | 32.2 |
| 359 | R_a32pr | Right Area anterior 32 prime | 5.1 | 27.1 | 28.6 |
| 360 | R_p24 | Right Area posterior 24 | 9.5 | 31.1 | 18.6 |
| 360 | R_p24 | Right Area posterior 24 | 8.6 | 35.7 | 11 |

HCP, human connectome project; MNI, Montreal neurological institute; Parcel Index, parcellation index.
