# Supplementary material for: Cell-Type-Specific Gene Modules Related to the Regional Homogeneity of Spontaneous Brain Activity and Their Associations With Common Brain Disorders
Source: Front Neurosci. 2021 Apr 20;15:639527. doi: 10.3389/fnins.2021.639527 (PMC8093778; doi:10.3389/fnins.2021.639527)
Supplement: Supplementary Table 2 — The normality test results of ME for all modules across neocortical regions. Note: The Kolmogorov-Smirnov test was applied to check the normality of ME for modules across neocortical regions. ME, module eigengene. [file Table_3.DOC]

| **Module Type** | **Kolmogorov-Smirnov Test** | | **Distribution Type** |
| --- | --- | --- | --- |
| Black | *D*=0.45052 | *P* value<2.2e-16 | no-normality |
| Darkgrey | *D*=0.44781 | *P* value<2.2e-16 | no-normality |
| Pink | *D*=0.44119 | *P* value<2.2e-16 | no-normality |
| Salmon | *D=*0.44413 | *P* value<2.2e-16 | no-normality |
| Grey60 | *D*=0.44956 | *P* value<2.2e-16 | no-normality |
| Orange | *D*=0.44851 | *P* value<2.2e-16 | no-normality |
| Darkgreen | *D*=0.44045 | *P* value<2.2e-16 | no-normality |
| Royalblue | *D*=0.44808 | *P* value<2.2e-16 | no-normality |
| Red | *D*=0.44314 | *P* value<2.2e-16 | no-normality |
| Lightcyan | *D*=0.44341 | *P* value<2.2e-16 | no-normality |
| Turquoise | *D*=0.44448 | *P* value<2.2e-16 | no-normality |
| Darkorange | *D*=0.44266 | *P* value<2.2e-16 | no-normality |
| Skyblue | *D*=0.44475 | *P* value<2.2e-16 | no-normality |
| Steelblue | *D*=0.45487 | *P* value<2.2e-16 | no-normality |
| Blue | *D*=0.44795 | *P* value<2.2e-16 | no-normality |
| Greenyellow | *D*=0.45045 | *P* value<2.2e-16 | no-normality |
| Saddlebrown | *D*=0.44596 | *P* value<2.2e-16 | no-normality |
| Cyan | *D*=0.4449 | *P* value<2.2e-16 | no-normality |
| Lightgreen | *D*=0.43958 | *P* value<2.2e-16 | no-normality |
| Green | *D*=0.44856 | *P* value<2.2e-16 | no-normality |
| Tan | *D*=0.45205 | *P* value<2.2e-16 | no-normality |
| Magenta | *D*=0.44447 | *P* value<2.2e-16 | no-normality |
| Yellow | *D*=0.43897 | *P* value<2.2e-16 | no-normality |
| Darkred | *D*=0.44794 | *P* value<2.2e-16 | no-normality |
| Lightyellow | *D*=0.44046 | *P* value<2.2e-16 | no-normality |
| Brown | *D*=0.44649 | *P* value<2.2e-16 | no-normality |
| Midnightblue | *D*=0.44508 | *P* value<2.2e-16 | no-normality |
| Purple | *D*=0.44052 | *P* value<2.2e-16 | no-normality |
| White | *D*=0.44773 | *P* value<2.2e-16 | no-normality |
| Grey | *D*=0.44017 | *P* value<2.2e-16 | no-normality |

**Table S2.** The normality test results of MEs for all modules cross neocortical regions.

Note: the Kolmogorov-Smirnov test was applied for checking the normality of MEs for modules cross neocortical regions. MEs, module eigengenes.
