# Supplementary material for: Cell-Type-Specific Gene Modules Related to the Regional Homogeneity of Spontaneous Brain Activity and Their Associations With Common Brain Disorders
Source: Front Neurosci. 2021 Apr 20;15:639527. doi: 10.3389/fnins.2021.639527 (PMC8093778; doi:10.3389/fnins.2021.639527)
Supplement: Supplementary Table 3 — The GWAS datasets of common brain disorders involved in this study. GWAS, genome-wide association studies; IGAP, International Genomics of Alzheimer’s Project; ILAE, International League Against Epilepsy; IMSGC, International Multiple Sclerosis Genetics Consortium; MEGASTROKE, International Stroke Genetics Consortium; PGC, Psychiatric Genomics Consortium; iPSYCH, Integrative Psychiatric Research Consortium. [file Table_4.DOC]

**Table S3.** The GWAS data sets of common neuropsychiatric disorders involved in this study.

| **Disorder Type** | **Disorder Name** | **Consortium/1st_Author** | **Sample Size** | **Publication** |
| --- | --- | --- | --- | --- |
| Neurological disorders | Alzheimer's disease (Discovery) | Iris E.Jansen | 455258 | Nat Genet. 2019 Mar;51(3):404-413. |
|  | Alzheimer's disease (Validation) | IGAP | 63926 | Nat Genet. 2019 Mar;51(3):414-430. |
|  | Parkinson's disease | Nathan Pankratz | 8477 | Ann Neurol. 2012 Mar;71(3):370-84. |
|  | Epilepsy | ILAE | 44889 | Nat Commun. 2018 Dec 10;9(1):5269. |
|  | Any stroke type | MEGASTROKE | 455258 | Nat Genet. 2018 Apr;50(4):524-537. |
|  | Multiple sclerosis (Discovery) | IMSGC | 115803 | Science. 2019 Sep 27;365(6460). |
|  | Multiple sclerosis (Validation) | IMSGC | 38589 | Nat Genet. 2013 Nov;45(11):1353-60. |
| Psychiatric disorders | Bipolar disorder | PGC | 51710 | Nat Genet. 2019 May;51(5):793-803. |
|  | Major depressive disorder | PGC | 18759 | Mol Psychiatry. 2013 Apr;18(4):497-511. |
|  | Schizophrenia | CLUZUK-PGC | 105318 | Nat Genet. 2018 Mar;50(3):381-389. |
|  | Attention-deficit/hyperactivity disorder | PGC | 53293 | Biol Psychiatry. 2018 Jun 15;83(12):1044-1053. |
|  | autism spectrum disorder | iPSYCH-PGC | 46351 | Mol Autism. 2017 May 22;8:21. |

GWAS, genome-wide association studies; IGAP, international genomics of Alzheimer's project; ILAE, international league against epilepsy; IMSGC, international multiple sclerosis genetics consortium; MEGASTROKE, international stroke genetics consortium; PGC, psychiatric genomics consortium; iPSYCH, integrative psychiatric research consortium.
