# Supplementary material for: Cell-Type-Specific Gene Modules Related to the Regional Homogeneity of Spontaneous Brain Activity and Their Associations With Common Brain Disorders
Source: Front Neurosci. 2021 Apr 20;15:639527. doi: 10.3389/fnins.2021.639527 (PMC8093778; doi:10.3389/fnins.2021.639527)
Supplement: Supplementary Table 4 — The significant ReHo-related modules in the discovery sample and replication samples. Note: the significant modules common to the three samples are labeled with bold font. r, correlation coefficients between ME and zReHo; Pc, Bonferroni-corrected P values; ReHo, regional homogeneity. [file Table_5.DOC]

**Table S4.** The significant ReHo-related modules in discovery sample and validation samples.

| **Module Type** | **Discovery Sample** | | **Replication Sample1** | **Replication Sample2** |
| --- | --- | --- | --- | --- |
| **Darkgrey** | | *r*=-0.35, *Pc*=3E-08 | *r*=-0.37, *Pc*=2E-09 | *r*=-0.5, *Pc*=1E-17 |
| **Salmon** | | *r*=0.21, *Pc*=0.01 | *r*=0.22, *Pc*=6E-03 | *r*=0.23, *Pc*=3E-03 |
| **Orange** | | *r*=0.2, *Pc*=0.03 | *r*=0.22, *Pc*=7E-03 | *r*=0.35, *Pc*=4E-08 |
| **Red** | | *r*=-0.23, *Pc*=2E-03 | *r*=-0.24, *Pc*=1E-03 | *r*=-0.35, *Pc*=4E-08 |
| **Darkorange** | | *r*=-0.32, *Pc*=7E-07 | *r*=-0.33, *Pc*=6E-07 | *r*=-0.37, *Pc*=4E-09 |
| **Skyblue** | | *r*=-0.26, *Pc*=4E-04 | *r*=-0.28, *Pc*=6E-05 | *r*=-0.31, *Pc*=3E-06 |
| Steelblue | | *r*=-0.13, *Pc*=0.9 | *r*=-0.16, *Pc*=0.2 | *r*=-0.3, *Pc*=5E-06 |
| **Blue** | | *r*=-0.38, *Pc*=1E-09 | *r*=-0.41, *Pc*=1E-11 | *r*=-0.57, *Pc*=5E-24 |
| **Greenyellow** | | *r*=-0.27, *Pc*=1E-04 | *r*=-0.3, *Pc*=6E-06 | *r*=-0.48, *Pc*=1E-16 |
| Saddlebrown | | *r*=0.18, *Pc*=0.06 | *r*=0.2, *Pc*=0.02 | *r*=0.2, *Pc*=0.02 |
| **Lightgreen** | | *r*=0.29, *Pc*=3E-05 | *r*=0.28, *Pc*=4E-05 | *r*=0.24, *Pc*=9E-04 |
| Green | | *r*=0.15, *Pc*=0.3 | *r*=0.16, *Pc*=0.3 | *r*=0.25, *Pc*=7E-04 |
| **Tan** | | *r*=0.31, *Pc*=3E-06 | *r*=0.33, *Pc*=4E-07 | *r*=0.43, *Pc*=1E-12 |
| **Yellow** | | *r*=0.21, *Pc*=0.02 | *r*=0.21, *Pc*=0.01 | *r*=0.25, *Pc*=6E-04 |
| Darkred | | *r*=-0.14, *Pc*=0.5 | *r*=-0.16, *Pc*=0.2 | *r*=-0.26, *Pc*=4E-04 |
| **Brown** | | *r*=0.51, *Pc*=5E-19 | *r*=0.53, *Pc*=2E-20 | *r*=0.61, *Pc*=5E-29 |
| **Midnightblue** | | *r*=0.38, *Pc*=8E-10 | *r*=0.41, *Pc*=3E-11 | *r*=0.55, *Pc*=9E-23 |
| Purple | | *r*=0.18, *Pc*=0.07 | *r*=0.18, *Pc*=0.07 | *r*=0.26, *Pc*=2E-04 |
| **White** | | *r*=0.36, *Pc*=2E-08 | *r*=0.35, *Pc*=5E-08 | *r*=0.37, *Pc*=6E-09 |
| Grey | | *r*=-0.072, *Pc*=1 | *r*=-0.069, *Pc*=1 | *r*=-0.19, *Pc*=0.03 |
| Number of significant modules in each sample | | 14 | 15 | 20 |
| Number of significant modules common to three samples | | 14 | | |

Note: the significant modules common to the three samples are labeled with bold font. *r*, correlation coefficients between ME and zReHo; *Pc*, Bonferroni corrected *P* value; ReHo, regional homogeneity.
