# Supplementary material for: Cell-Type-Specific Gene Modules Related to the Regional Homogeneity of Spontaneous Brain Activity and Their Associations With Common Brain Disorders
Source: Front Neurosci. 2021 Apr 20;15:639527. doi: 10.3389/fnins.2021.639527 (PMC8093778; doi:10.3389/fnins.2021.639527)
Supplement: Supplementary Table 5 — The results of cell-type-specific enrichment analysis in two GSE samples. The significant cell-type-specific ReHo-related modules in both GSE samples are labeled with bold font. Astro, astrocytoma; Endo, endothelium; GSE, gene series expression; Micro, microglia; Neuro, neuron; Oligo, oligodendrocyte; Pc, Bonferroni-corrected P values; ReHo, regional homogeneity. [file Table_6.DOC]

**Table S5.** The results of cell-type specific enrichment analysis in two GSE samples.

| **Module Type** | **Cell Type** | **GSE73721** | |  | **GSE67835** | |
| --- | --- | --- | --- | --- | --- | --- |
|  |  | ***P* value** | ***Pc* value** |  | ***P* value** | ***Pc* value** |
| **Brown** | **Neuro** | **7.98E-21** | **2.17E-20** |  | **4.81E-04** | **1.35E-03** |
| Brown | Astro | 0.66 | 1 |  | 0.29 | 1 |
| Brown | Micro | 0.023 | 0.11 |  | 0.21 | 1 |
| Brown | Oligo | 0.39 | 1 |  | 0.73 | 1 |
| Brown | Endo | 0.044 | 0.26 |  | 2.24E-03 | 1.41E-02 |
| **Blue** | **Neuro** | **1.26E-09** | **6.31E-09** |  | **1.22E-04** | **7.73E-04** |
| Blue | Astro | 1.52E-02 | 0.076 |  | 3.93E-02 | 0.19 |
| Blue | Micro | 0.043 | 0.21 |  | 0.24 | 1 |
| Blue | Oligo | 0.52576 | 1 |  | 6.72E-03 | 0.056 |
| **Blue** | **Endo** | **7.36E-03** | **1.18E-02** |  | **2.39E-03** | **1.87E-02** |
| Red | Neuro | 1 | 1 |  | 1 | 1 |
| **Red** | **Astro** | **1.50E-217** | **6.02E-219** |  | **2.58E-69** | **1.29E-68** |
| Red | Micro | 0.11 | 0.59 |  | 0.13 | 0.67 |
| Red | Oligo | 0.63 | 1 |  | 9.91E-05 | 5.03E-04 |
| Red | Endo | 0.045 | 0.22 |  | 1 | 1 |
| Darkorange | Neuro | 0.014 | 0.074 |  | 1 | 1 |
| Darkorange | Astro | 0.051 | 0.25 |  | 1 | 1 |
| **Darkorange** | **Micro** | **1.927E-105** | **8.44E-105** |  | **1.09E-102** | **5.43E-102** |
| Darkorange | Oligo | 0.17 | 0.88 |  | 0.054 | 0.27 |
| Darkorange | Endo | 0.093 | 0.46 |  | 0.17 | 0.89 |
| Yellow | Neuro | 0.35 | 1 |  | 1.39E-02 | 0.069 |
| Yellow | Astro | 2.32E-02 | 0.11 |  | 1 | 1 |
| **Yellow** | **Micro** | **1.23E-03** | **8.22E-03** |  | **2.33E-03** | **1.17E-02** |
| Yellow | Oligo | 1 | 1 |  | 0.13 | 0.66 |
| Yellow | Endo | 0.71 | 1 |  | 1.56E-02 | 0.077 |
| White | Neuro | 1.22E-10 | 6.42E-10 |  | 0.40 | 1 |
| White | Astro | 0.58 | 1 |  | 2.12E-02 | 0.11 |
| White | Micro | 0.19 | 0.95 |  | 0.59 | 1 |
| White | Oligo | 0.17 | 0.88 |  | 0.26 | 1 |
| White | Endo | 1.52E-02 | 0.076 |  | 0.49 | 1 |
| Skyblue | Neuro | 1.62E-05 | 8.34E-05 |  | 0.59 | 1 |
| Skyblue | Astro | 0.37 | 1 |  | 0.12 | 0.64 |
| Skyblue | Micro | 0.58 | 1 |  | 0.26 | 1 |
| Skyblue | Oligo | 0.27 | 1 |  | 0.080 | 0.40 |
| Skyblue | Endo | 1 | 1 |  | 1 | 1 |
| Midnightblue | Neuro | 7.33E-05 | 3.73E-04 |  | 0.43 | 1 |
| Midnightblue | Astro | 0.04 | 0.23 |  | 0.10 | 0.52 |
| Midnightblue | Micro | 0.83 | 1 |  | 1 | 1 |
| Midnightblue | Oligo | 0.59 | 1 |  | 0.67 | 1 |
| Midnightblue | Endo | 0.71 | 1 |  | 0.21 | 1 |
| Lightgreen | Neuro | 0.83 | 1 |  | 3.78E-03 | 1.88E-02 |
| Lightgreen | Astro | 0.095 | 0.48 |  | 0.38 | 1 |
| Lightgreen | Micro | 0.38 | 1 |  | 0.65 | 1 |
| Lightgreen | Oligo | 0.079 | 0.39 |  | 0.82 | 1 |
| Lightgreen | Endo | 0.31 | 1 |  | 0.56 | 1 |
| Greenyellow | Neuro | 0.017 | 0.085 |  | 0.19 | 1 |
| Greenyellow | Astro | 0.19 | 0.99 |  | 1 | 1 |
| Greenyellow | Micro | 0.54 | 1 |  | 0.15 | 0.79 |
| Greenyellow | Oligo | 0.42 | 1 |  | 1.51E-02 | 0.076 |
| Greenyellow | Endo | 5.15E-03 | 3.02E-02 |  | 0.080 | 0.41 |
| Darkgrey | Neuro | 6.28E-03 | 2.96E-02 |  | 0.26 | 1 |
| Darkgrey | Astro | 0.11 | 0.56 |  | 0.69 | 1 |
| Darkgrey | Micro | 5.26E-04 | 2.63E-03 |  | 0.075 | 0.37 |
| Darkgrey | Oligo | 0.74 | 1 |  | 0.80 | 1 |
| Darkgrey | Endo | 0.37 | 1 |  | 1 | 1 |
| Tan | Neuro | 0.77 | 1 |  | 0.036 | 0.18 |
| Tan | Astro | 0.049 | 0.24 |  | 0.049 | 0.24 |
| Tan | Micro | 0.64 | 1 |  | 0.27 | 1 |
| Tan | Oligo | 0.071 | 0.35 |  | 0.11 | 0.53 |
| Tan | Endo | 0.67 | 1 |  | 0.78 | 1 |
| Orange | Neuro | 0.33 | 1 |  | 0.61 | 1 |
| Orange | Astro | 0.58 | 1 |  | 0.53 | 1 |
| Orange | Micro | 0.79 | 1 |  | 0.79 | 1 |
| Orange | Oligo | 1 | 1 |  | 0.27 | 1 |
| Orange | Endo | 0.15 | 0.74 |  | 0.17 | 0.88 |
| Salmon | Neuro | 5.06E-11 | 2.53E-10 |  | 0.54 | 1 |
| Salmon | Astro | 1 | 1 |  | 1 | 1 |
| Salmon | Micro | 0.200 | 1 |  | 0.015 | 0.076 |
| Salmon | Oligo | 0.097 | 0.48 |  | 0.050 | 0.25 |
| Salmon | Endo | 0.55 | 1 |  | 0.099 | 0.49 |

Note: the significant cell-type specific ReHo-related modules in both GSE samples are labeled with bold font. Astro, astrocytoma; Endo, endothelium; GSE, gene serial; Micro, microglia; Neuro, neuron; Oligo, oligodendrocyte; *Pc*, Bonferroni corrected *P* value; ReHo, regional homogeneity.
