# Supplementary material for: Cell-Type-Specific Gene Modules Related to the Regional Homogeneity of Spontaneous Brain Activity and Their Associations With Common Brain Disorders
Source: Front Neurosci. 2021 Apr 20;15:639527. doi: 10.3389/fnins.2021.639527 (PMC8093778; doi:10.3389/fnins.2021.639527)
Supplement: Supplementary Table 6 — The significant GO terms in ReHo-related cell-type-specific modules. BP, biological process; CC, cellular component; FDR, the corrected P values with the false discovery rate method; GO, gene ontology; MF, molecular function; ReHo, regional homogeneity. [file Table_7.DOC]

**Table S6.** The significant GO terms in ReHo-related cell-type specific modules.

| **Module** | **GO number** | **Description** | **Size** | **Expect** | **Ratio** | ***P* value** | **FDR** | **Category** |
| --- | --- | --- | --- | --- | --- | --- | --- | --- |
| Brown | GO:1990351 | transporter complex | 332 | 20.622 | 2.0366 | 7.5345E-06 | 0.0013 | CC |
| Brown | GO:0098531 | transcription factor activity, direct ligand regulated sequence-specific DNA binding | 48 | 2.9761 | 4.0321 | 0 | 0.0073 | MF |
| Brown | GO:0046873 | metal ion transmembrane transporter activity | 440 | 27.281 | 1.7228 | 0.0002 | 0.0165 | MF |
| Blue | GO:0023061 | signal release | 456 | 28.24 | 1.8059 | 0 | 0.0175 | BP |
| Blue | GO:0097060 | synaptic membrane | 430 | 27.819 | 1.7614 | 0.0001 | 0.0059 | CC |
| Blue | GO:0022803 | passive transmembrane transporter activity | 457 | 28.521 | 1.683 | 0.0003 | 0.0414 | MF |
| Blue | GO:0051959 | dynein light intermediate chain binding | 29 | 1.8099 | 4.4203 | 0.0003 | 0.0414 | MF |
| Red | GO:0006520 | cellular amino acid metabolic process | 318 | 8.5905 | 3.143 | 1.52E-07 | 0.0001 | BP |
| Red | GO:0007265 | Ras protein signal transduction | 437 | 11.805 | 2.4565 | 8.16E-06 | 0.001 | BP |
| Red | GO:0006631 | fatty acid metabolic process | 357 | 9.6441 | 2.5923 | 0.00E+00 | 0.0015 | BP |
| Red | GO:0072507 | divalent inorganic cation homeostasis | 494 | 13.345 | 2.1731 | 1.00E-04 | 0.0051 | BP |
| Red | GO:0001667 | ameboidal-type cell migration | 381 | 10.292 | 2.3318 | 1.00E-04 | 0.0063 | BP |
| Red | GO:0090596 | sensory organ morphogenesis | 250 | 6.7536 | 2.6653 | 2.00E-04 | 0.0071 | BP |
| Red | GO:0001763 | morphogenesis of a branching structure | 196 | 5.2948 | 2.833 | 3.00E-04 | 0.0094 | BP |
| Red | GO:0051961 | negative regulation of nervous system development | 297 | 8.0232 | 2.3681 | 5.00E-04 | 0.0138 | BP |
| Red | GO:0072089 | stem cell proliferation | 109 | 2.9446 | 3.3961 | 7.00E-04 | 0.0183 | BP |
| Red | GO:0001525 | angiogenesis | 487 | 13.156 | 1.9763 | 8.00E-04 | 0.0187 | BP |
| Red | GO:0031012 | extracellular matrix | 496 | 13.584 | 2.0613 | 0.0002 | 0.0124 | CC |
| Red | GO:0005911 | cell-cell junction | 441 | 12.077 | 2.1528 | 0.0002 | 0.0124 | CC |
| Red | GO:0031253 | cell projection membrane | 335 | 9.1744 | 2.398 | 0.0001 | 0.0124 | CC |
| Red | GO:1901681 | sulfur compound binding | 240 | 6.7002 | 3.2835 | 9.91E-07 | 0.0003 | MF |
| Red | GO:0016903 | oxidoreductase activity, acting on the aldehyde or oxo group of donors | 43 | 1.2005 | 5.8311 | 2.00E-04 | 0.0119 | MF |
| Darkorange | GO:0002269 | leukocyte activation involved in inflammatory response | 31 | 0.1947 | 25.68 | 1.31E-06 | 1.00E-13 | BP |
| Darkorange | GO:0002446 | neutrophil mediated immunity | 496 | 3.1153 | 7.383 | 3.04E-14 | 7.67E-12 | BP |
| Darkorange | GO:0002697 | regulation of immune effector process | 381 | 2.393 | 7.522 | 2.05E-11 | 1.74E-09 | BP |
| Darkorange | GO:0050900 | leukocyte migration | 419 | 2.6317 | 6.8398 | 9.78E-11 | 6.93E-09 | BP |
| Darkorange | GO:0002521 | leukocyte differentiation | 496 | 3.1153 | 6.099 | 1.94E-10 | 1.27E-08 | BP |
| Darkorange | GO:0019882 | antigen processing and presentation | 171 | 1.074 | 11.173 | 7.08E-10 | 4.30E-08 | BP |
| Darkorange | GO:0022407 | regulation of cell-cell adhesion | 383 | 2.4056 | 6.6513 | 1.82E-09 | 9.11E-08 | BP |
| Darkorange | GO:0071706 | tumor necrosis factor superfamily cytokine production | 139 | 0.873 | 11.454 | 1.60E-08 | 7.15E-07 | BP |
| Darkorange | GO:0050866 | negative regulation of cell activation | 179 | 1.1243 | 8.0052 | 1.81E-06 | 1.00E-04 | BP |
| Darkorange | GO:0031349 | positive regulation of defense response | 427 | 2.6819 | 4.8473 | 2.54E-06 | 1.00E-04 | BP |
| Darkorange | GO:0042611 | MHC protein complex | 21 | 0.1417 | 49.402 | 5.01E-11 | 5.61E-09 | CC |
| Darkorange | GO:0070820 | tertiary granule | 163 | 1.0998 | 9.0924 | 1.21E-07 | 5.18E-06 | CC |
| Darkorange | GO:0005766 | primary lysosome | 155 | 1.0458 | 6.6932 | 1.00E-04 | 1.90E-03 | CC |
| Darkorange | GO:0003823 | antigen binding | 55 | 0.3482 | 14.361 | 0 | 0.0023 | MF |
| Darkorange | GO:0016502 | nucleotide receptor activity | 22 | 0.1393 | 28.723 | 9.9665E-06 | 0.0023 | MF |
| Darkorange | GO:0019865 | immunoglobulin binding | 23 | 0.1456 | 20.605 | 4.00E-04 | 0.0222 | MF |
| Yellow | GO:0046390 | ribose phosphate biosynthetic process | 255 | 9.9885 | 2.9033 | 2.5954E-07 | 0.0002206 | BP |
| Yellow | GO:0061919 | process utilizing autophagic mechanism | 473 | 18.528 | 2.105 | 9.6166E-06 | 0.0020435 | BP |
| Yellow | GO:0006839 | mitochondrial transport | 237 | 9.2834 | 2.4775 | 0.000058911 | 0.0083457 | BP |
| Yellow | GO:0006399 | tRNA metabolic process | 183 | 7.1682 | 2.6506 | 0.00010469 | 0.011124 | BP |
| Yellow | GO:0009123 | nucleoside monophosphate metabolic process | 321 | 12.574 | 2.1473 | 0.00016257 | 0.015354 | BP |
| Yellow | GO:0005743 | mitochondrial inner membrane | 455 | 20.633 | 1.9871 | 0.000018654 | 0.0032085 | CC |
| Yellow | GO:1905368 | peptidase complex | 89 | 4.0358 | 3.2212 | 0.00017359 | 0.0099525 | CC |
| Yellow | GO:0099023 | tethering complex | 67 | 3.0382 | 3.2914 | 0.0008086 | 0.027816 | CC |

BP, biological process; CC, cellular component; FDR, the corrected *P* value with false discovery rate method; GO, gene ontology; MF, molecular function; ReHo, regional homogeneity.
