# Supplementary material for: Cell-Type-Specific Gene Modules Related to the Regional Homogeneity of Spontaneous Brain Activity and Their Associations With Common Brain Disorders
Source: Front Neurosci. 2021 Apr 20;15:639527. doi: 10.3389/fnins.2021.639527 (PMC8093778; doi:10.3389/fnins.2021.639527)
Supplement: Supplementary Table 7 — The enrichment results for 10 common brain disorders in this study. The significant modules enriched for brain disorders are labeled with bold font. AD, Alzheimer’s disease; ADHD, attention deficit hyperactivity disorder; ASD, autism spectrum disorder; BP, bipolar disorder; IGAP, International Genomics of Alzheimer’s Project; ILAE, International League Against Epilepsy; IMSGC, International Multiple Sclerosis Genetics Consortium; iPSYCH, Integrative Psychiatric Research Consortium; MDD, major depressive disorder; MEGASTROKE, International Stroke Genetics Consortium; MS, multiple Sclerosis; PD, Parkinson’s disease; PGC, Psychiatric Genomics Consortium; Pc, Bonferroni-corrected P values; SCZ, schizophrenia. [file Table_8.DOC]

| **The five common neurological disorders** | | | | | | | | | | | | | | | | | | | | |
| --- | --- | --- | --- | --- | --- | --- | --- | --- | --- | --- | --- | --- | --- | --- | --- | --- | --- | --- | --- | --- |
| **Module** | **AD.Jansen.2019 (Discovery)** | |  | **AD.IGAP.2019 (Validation)** | |  | **Epilepsy.ILAE.2018** | |  | **MS.MSGC.2018**  **(Discovery)** | |  | **MS.MSGC.2013 (Validation)** | |  | **PD.Pankratz.2012** | |  | **Stroke.MEGASTROKE.2018** | |
|  | ***P*** | ***Pc*** |  | ***P*** | ***Pc*** |  | ***P*** | ***Pc*** |  | ***P*** | ***Pc*** |  | ***P*** | ***Pc*** |  | ***P*** | ***Pc*** |  | ***P*** | ***Pc*** |
| Blue | 0.69758 | 1 |  | 0.7709 | 1 |  | 0.5287 | 1 |  | 0.93479 | 1 |  | 0.85679 | 1 |  | 0.50199 | 1 |  | 0.87365 | 1 |
| Brown | 0.73482 | 1 |  | 0.73852 | 1 |  | 0.26602 | 1 |  | 0.75495 | 1 |  | 0.97373 | 1 |  | 0.066328 | 0.33164 |  | 0.39613 | 1 |
| **Darkorange** | **3.77E-05** | **1.88E-04** |  | **3.55E-04** | **1.78E-03** |  | 0.26551 | 1 |  | **1.65E-03** | **8.26E-03** |  | **6.24E-03** | **3.12E-02** |  | 0.066942 | 0.33471 |  | 0.94953 | 1 |
| Red | 0.40508 | 1 |  | 0.7423 | 1 |  | 0.59065 | 1 |  | 0.78279 | 1 |  | 0.56033 | 1 |  | 0.91988 | 1 |  | 0.089543 | 0.447715 |
| Yellow | 0.42298 | 1 |  | 0.43892 | 1 |  | 0.2487 | 1 |  | 0.66091 | 1 |  | 0.36594 | 1 |  | 0.29348 | 1 |  | 0.769 | 1 |

**Table S7.** The Enrichment results for 10 common neuropsychiatric disorders in this study.

| **The five common psychiatric disorders** | | | | | | | | | | | | | | |
| --- | --- | --- | --- | --- | --- | --- | --- | --- | --- | --- | --- | --- | --- | --- |
| **Module** | **ADHD.PGC.2017** | |  | **ASD.iPSYCH-PGC.2017** | |  | **BP.PGC.2018** | |  | **MDD.PGC.2013** | |  | **SCZ.PGC.2019** | |
|  | ***P*** | ***Pc*** |  | ***P*** | ***Pc*** |  | ***P*** | ***Pc*** |  | ***P*** | ***Pc*** |  | ***P*** | ***Pc*** |
| Blue | 0.72523 | 1 |  | 0.98895 | 1 |  | 0.8036 | 1 |  | 0.16946 | 0.8473 |  | 0.92863 | 1 |
| Brown | 0.83272 | 1 |  | 0.48316 | 1 |  | 0.070563 | 0.352815 |  | 0.84407 | 1 |  | 0.2971 | 1 |
| Darkorange | 0.78915 | 1 |  | 0.39844 | 1 |  | 0.97159 | 1 |  | 0.80118 | 1 |  | 0.9827 | 1 |
| Red | 0.61577 | 1 |  | 0.848 | 1 |  | 0.48695 | 1 |  | 0.89111 | 1 |  | 0.95658 | 1 |
| Yellow | 0.57254 | 1 |  | 0.43683 | 1 |  | 0.57636 | 1 |  | 0.89276 | 1 |  | 0.27664 | 1 |

Note: the significant modules enriched for neuropsychiatric disorders are labeled with bold font. AD, Alzheimer's disease; ADHD, attention deficit hyperactivity disorder; ASD, autism spectrum disorder; BP, bipolar disorder; IGAP, international genomics of Alzheimer's project; ILAE, international league against epilepsy; IMSGC, international multiple sclerosis genetics consortium; iPSYCH, integrative psychiatric research consortium; MDD, major depressive disorder; MEGASTROKE, international stroke genetics consortium; MS, multiple sclerosis; PD, Parkinson’s disease; PGC, psychiatric genomics consortium; *Pc*, Bonferroni corrected *P* value; SCZ, schizophrenia.
