# Supplementary material for: Cell-Type-Specific Gene Modules Related to the Regional Homogeneity of Spontaneous Brain Activity and Their Associations With Common Brain Disorders
Source: Front Neurosci. 2021 Apr 20;15:639527. doi: 10.3389/fnins.2021.639527 (PMC8093778; doi:10.3389/fnins.2021.639527)
Supplement: Supplementary Table 8 — The MAGMA gene level analysis results in the microglial module for AD and MS. The significant genes common to both discovery and replication GWAS datasets are labeled with bold font. AD, Alzheimer’s disease; IGAP, International Genomics of Alzheimer’s Project; IMSGC, International Multiple Sclerosis Genetics Consortium; MAGMA, multi-marker analysis of genomic annotation; MS, multiple sclerosis. [file Table_9.DOC]

|  | | | | | | | | | | | | | | | | | | | | |
| --- | --- | --- | --- | --- | --- | --- | --- | --- | --- | --- | --- | --- | --- | --- | --- | --- | --- | --- | --- | --- |
|  | | | | | | | | | | | | | | | | | | | | |
|  | | | | | | | | | | | | | | | | | | | | |
|  | | | | | | | | | | | | | | | | | | | | |
|  | | | | | | | | | | | | | | | | | | | | |
| **Table S8.** The MAGMA gene level analysis results in microglia module for AD and MS. | | | | | | | | | | | | | | | | | | | | |
| **AD_Jansen.2019** | | |  | | **AD.IGAP.2019** | | | |  | | **MS.MSGC.2018** | | | |  | | **MS.MSGC.2013** | | | |
| **Gene Symbol** | ***P* value** | ***Pc* value** | |  | | **Gene Symbol** | ***P* value** | ***Pc* value** | |  | | **Gene Symbol** | ***P* value** | ***Pc* value** | |  | | **Gene Symbol** | ***P* value** | ***Pc* value** |
| ***MS4A4A*** | **4.93E-15** | **4.98E-13** | |  | | ***MS4A4A*** | **8.17E-15** | **8.25E-13** | |  | | ***IFI30*** | **4.16E-12** | **3.91E-10** | |  | | *C2* | 7.91E-93 | 6.64E-91 |
| ***HLA-DRA*** | **4.60E-10** | **4.65E-08** | |  | | ***HLA-DRB5*** | **4.69E-08** | **4.74E-06** | |  | | ***RGS1*** | **6.60E-11** | **6.20E-09** | |  | | *HLA-DRA* | 1.2E-92 | 1.01E-90 |
| ***TREM2*** | **1.67E-07** | **1.69E-05** | |  | | ***CD33*** | **2.28E-07** | **2.30E-05** | |  | | *HCLS1* | 5.01E-07 | 4.71E-05 | |  | | *AIF1* | 1.55E-85 | 1.30E-83 |
| ***HLA-DRB5*** | **1.30E-06** | **1.31E-04** | |  | | ***TREM2*** | **3.59E-06** | **3.63E-04** | |  | | ***CD86*** | **1.40E-06** | **1.32E-04** | |  | | *LST1* | 2.01E-68 | 1.69E-66 |
| ***CD33*** | **3.07E-06** | **3.10E-04** | |  | | ***HLA-DRA*** | **1.23E-05** | **1.24E-03** | |  | | *PTPRC* | 2.32E-05 | 2.18E-03 | |  | | *HLA-DMB* | 8.72E-65 | 7.32E-63 |
| *PLCG2* | 3.42E-06 | 3.45E-04 | |  | | *LAPTM5* | 4.47E-05 | 4.51E-03 | |  | | *ITGAM* | 9.88E-05 | 9.29E-03 | |  | | *HLA-DMA* | 4.43E-44 | 3.72E-42 |
| *SUSD3* | 1.81E-04 | 1.83E-02 | |  | | *LILRB4* | 2.97E-03 | 3.00E-01 | |  | | *CD68* | 5.51E-04 | 5.18E-02 | |  | | *HLA-DPA1* | 5.32E-17 | 4.47E-15 |
| *SYK* | 1.84E-03 | 1.86E-01 | |  | | *ITGAM* | 3.75E-03 | 3.78E-01 | |  | | *PLCG2* | 2.62E-03 | 2.47E-01 | |  | | *HLA-DOA* | 8.75E-16 | 7.35E-14 |
| *HAVCR2* | 2.65E-03 | 2.67E-01 | |  | | *TLR2* | 3.84E-03 | 3.88E-01 | |  | | *HAVCR2* | 2.67E-03 | 2.51E-01 | |  | | *HLA-DPB1* | 1.88E-14 | 1.58E-12 |
| *ITGAM* | 3.02E-03 | 3.05E-01 | |  | | *CMTM7* | 6.59E-03 | 6.66E-01 | |  | | *EVI2B* | 4.54E-03 | 4.27E-01 | |  | | ***RGS1*** | **3.14E-13** | **2.64E-11** |
| *BLNK* | 4.40E-03 | 4.44E-01 | |  | | *BLNK* | 1.13E-02 | 1 | |  | | *HLA-DRA* | 4.87E-03 | 4.58E-01 | |  | | ***CD86*** | **5.58E-11** | **4.69E-09** |
| *ITGAX* | 7.59E-03 | 7.67E-01 | |  | | *HHEX* | 1.32E-02 | 1 | |  | | *DOCK8* | 7.11E-03 | 6.69E-01 | |  | | *HHEX* | 6.38E-08 | 5.36E-06 |
| *HHEX* | 9.68E-03 | 9.78E-01 | |  | | *AIF1* | 1.51E-02 | 1 | |  | | *HLA-DPA1* | 7.75E-03 | 7.29E-01 | |  | | ***IFI30*** | **1.07E-07** | **8.99E-06** |
| *LAPTM5* | 1.42E-02 | 1 | |  | | *SLC2A5* | 1.69E-02 | 1 | |  | | *HLA-DPB1* | 7.75E-03 | 7.29E-01 | |  | | *FPR1* | 9.77E-06 | 8.21E-04 |
| *IFI30* | 1.70E-02 | 1 | |  | | *LCP1* | 2.80E-02 | 1 | |  | | *FYB1* | 1.22E-02 | 1 | |  | | *SLC15A3* | 0.0001528 | 1.28E-02 |
| *LILRB4* | 2.01E-02 | 1 | |  | | *FPR1* | 2.96E-02 | 1 | |  | | *RNASET2* | 1.73E-02 | 1 | |  | | *GPR183* | 0.0009707 | 8.15E-02 |
| *FGL2* | 2.20E-02 | 1 | |  | | *EVI2B* | 3.24E-02 | 1 | |  | | *SELPLG* | 2.38E-02 | 1 | |  | | *RNASET2* | 0.0019959 | 1.68E-01 |
| *SCIN* | 2.76E-02 | 1 | |  | | *HAVCR2* | 3.25E-02 | 1 | |  | | *RASAL3* | 2.50E-02 | 1 | |  | | *HCST* | 0.0072358 | 6.08E-01 |
| *FCGR2A* | 3.17E-02 | 1 | |  | | *ITGAX* | 3.49E-02 | 1 | |  | | *TREM2* | 4.78E-02 | 1 | |  | | *SYK* | 0.010838 | 9.10E-01 |
| *RGS18* | 3.77E-02 | 1 | |  | | *PLCG2* | 3.55E-02 | 1 | |  | | *CYBA* | 5.58E-02 | 1 | |  | | *DOCK8* | 0.011492 | 9.65E-01 |
| *CYTH4* | 3.80E-02 | 1 | |  | | *TMC8* | 3.63E-02 | 1 | |  | | *ADAP2* | 7.30E-02 | 1 | |  | | *C3* | 0.014036 | 1 |
| *LY86* | 4.11E-02 | 1 | |  | | *P2RY13* | 4.13E-02 | 1 | |  | | *PYCARD* | 8.56E-02 | 1 | |  | | *PTPRC* | 0.029885 | 1 |
| *AIF1* | 4.30E-02 | 1 | |  | | *FCGR2A* | 4.57E-02 | 1 | |  | | *IGSF6* | 8.61E-02 | 1 | |  | | *ADAM28* | 0.035 | 1 |
| *FCGR3A* | 4.31E-02 | 1 | |  | | *C2* | 6.60E-02 | 1 | |  | | *TLR1* | 8.69E-02 | 1 | |  | | *RGS10* | 0.044742 | 1 |
| *SLC2A5* | 4.75E-02 | 1 | |  | | *WDFY4* | 6.70E-02 | 1 | |  | | *KCNQ1* | 9.08E-02 | 1 | |  | | *HCLS1* | 0.055174 | 1 |
| *LCP1* | 4.86E-02 | 1 | |  | | *CLEC9A* | 6.78E-02 | 1 | |  | | *LY86* | 9.53E-02 | 1 | |  | | *ITGAM* | 0.062468 | 1 |
| *CLEC9A* | 5.55E-02 | 1 | |  | | *SYK* | 8.34E-02 | 1 | |  | | *TYROBP* | 9.70E-02 | 1 | |  | | *TREM2* | 0.093499 | 1 |
| *HLA-DOA* | 5.96E-02 | 1 | |  | | *RGS10* | 9.64E-02 | 1 | |  | | *MYO1F* | 1.04E-01 | 1 | |  | | *FCGR3A* | 0.12768 | 1 |
| *TMC8* | 8.27E-02 | 1 | |  | | *TYROBP* | 9.80E-02 | 1 | |  | | *ITGAX* | 1.14E-01 | 1 | |  | | *CCR1* | 0.13696 | 1 |
| *CD68* | 8.55E-02 | 1 | |  | | *CD74* | 1.18E-01 | 1 | |  | | *C1QA* | 1.20E-01 | 1 | |  | | *PYCARD* | 0.15769 | 1 |
| *CCR1* | 1.01E-01 | 1 | |  | | *OLFML3* | 1.27E-01 | 1 | |  | | *C1QC* | 1.20E-01 | 1 | |  | | *ITGAX* | 0.17597 | 1 |
| *FPR1* | 1.07E-01 | 1 | |  | | *P2RY12* | 1.46E-01 | 1 | |  | | *CX3CR1* | 1.23E-01 | 1 | |  | | *LRMDA* | 0.18906 | 1 |
| *C2* | 1.19E-01 | 1 | |  | | *LRMDA* | 1.50E-01 | 1 | |  | | *SLC15A3* | 1.49E-01 | 1 | |  | | *ADAP2* | 0.2358 | 1 |
| *PYCARD* | 1.45E-01 | 1 | |  | | *CD68* | 1.56E-01 | 1 | |  | | *APBB1IP* | 1.54E-01 | 1 | |  | | *PLCG2* | 0.24337 | 1 |
| *MYO1F* | 1.57E-01 | 1 | |  | | *CYTH4* | 1.68E-01 | 1 | |  | | *LPAR6* | 0.15493 | 1 | |  | | *TMC8* | 0.24629 | 1 |
| *TNFAIP8L2* | 1.73E-01 | 1 | |  | | *PYCARD* | 1.87E-01 | 1 | |  | | *RGS10* | 1.60E-01 | 1 | |  | | *ALOX5AP* | 0.25209 | 1 |
| *RNASET2* | 1.78E-01 | 1 | |  | | *SUSD3* | 1.88E-01 | 1 | |  | | *PIGP* | 1.61E-01 | 1 | |  | | *CYTL1* | 0.29383 | 1 |
| *PTPRC* | 1.81E-01 | 1 | |  | | *CD84* | 1.91E-01 | 1 | |  | | *SLC7A7* | 1.71E-01 | 1 | |  | | *CMTM7* | 0.31161 | 1 |
| *CX3CR1* | 1.84E-01 | 1 | |  | | *FCGR3A* | 2.30E-01 | 1 | |  | | *WDFY4* | 2.22E-01 | 1 | |  | | *OLFML3* | 0.32125 | 1 |
| *CD300A* | 2.00E-01 | 1 | |  | | *CD300A* | 2.38E-01 | 1 | |  | | *OLFML3* | 2.63E-01 | 1 | |  | | *P2RY13* | 0.33063 | 1 |
| *MNDA* | 2.08E-01 | 1 | |  | | *TMEM106A* | 2.62E-01 | 1 | |  | | *C1QB* | 2.67E-01 | 1 | |  | | *P2RY12* | 0.33063 | 1 |
| *TYROBP* | 2.25E-01 | 1 | |  | | *PIGP* | 2.75E-01 | 1 | |  | | *C3* | 2.70E-01 | 1 | |  | | *CD84* | 0.33396 | 1 |
| *EVI2B* | 2.31E-01 | 1 | |  | | *HCST* | 2.76E-01 | 1 | |  | | *HK2* | 2.72E-01 | 1 | |  | | *SCIN* | 0.34197 | 1 |
| *TLR1* | 2.78E-01 | 1 | |  | | *LPAR6* | 0.28188 | 1 | |  | | *IL13RA1* | 2.96E-01 | 1 | |  | | *TYROBP* | 0.343 | 1 |
| *P2RY13* | 2.84E-01 | 1 | |  | | *CX3CR1* | 2.90E-01 | 1 | |  | | *BLNK* | 3.07E-01 | 1 | |  | | *CYTH4* | 0.3431 | 1 |
| *LST1* | 2.98E-01 | 1 | |  | | *RGS18* | 2.97E-01 | 1 | |  | | *LRMDA* | 3.10E-01 | 1 | |  | | *SLC7A7* | 0.36 | 1 |
| *B3GNT5* | 2.99E-01 | 1 | |  | | *FCGR1B* | 3.06E-01 | 1 | |  | | *RHBDF2* | 3.15E-01 | 1 | |  | | *SELPLG* | 0.376 | 1 |
| *CYTL1* | 3.01E-01 | 1 | |  | | *LST1* | 3.12E-01 | 1 | |  | | *CD74* | 3.37E-01 | 1 | |  | | *RGS18* | 0.426 | 1 |
| *C3* | 3.30E-01 | 1 | |  | | *HK2* | 3.47E-01 | 1 | |  | | *SYK* | 3.38E-01 | 1 | |  | | *MNDA* | 0.428 | 1 |
| *SELPLG* | 3.59E-01 | 1 | |  | | *SLC15A3* | 3.69E-01 | 1 | |  | | *HCST* | 3.40E-01 | 1 | |  | | *CD33* | 0.437 | 1 |
| *LPAR6* | 0.38117 | 1 | |  | | *MS4A7* | 3.70E-01 | 1 | |  | | *LAPTM5* | 4.08E-01 | 1 | |  | | *ALOX5* | 0.48172 | 1 |
| *CD84* | 3.82E-01 | 1 | |  | | *RASAL3* | 3.75E-01 | 1 | |  | | *ALOX5* | 4.12E-01 | 1 | |  | | *FCGR1B* | 0.505 | 1 |
| *ITGB2* | 4.12E-01 | 1 | |  | | *RGS1* | 3.84E-01 | 1 | |  | | *TNFAIP8L2* | 4.13E-01 | 1 | |  | | *ITGB2* | 0.51695 | 1 |
| *CYBA* | 4.15E-01 | 1 | |  | | *HLA-DOA* | 3.97E-01 | 1 | |  | | *CH25H* | 4.17E-01 | 1 | |  | | *TLR2* | 0.57843 | 1 |
| *VAMP8* | 4.22E-01 | 1 | |  | | *ALOX5* | 4.16E-01 | 1 | |  | | *FCGR2A* | 4.22E-01 | 1 | |  | | *PIGP* | 0.584 | 1 |
| *ALG14* | 4.33E-01 | 1 | |  | | *DOCK8* | 4.18E-01 | 1 | |  | | *P2RY12* | 4.36E-01 | 1 | |  | | *SLC2A5* | 0.632 | 1 |
| *FYB1* | 4.33E-01 | 1 | |  | | *HLA-DPB1* | 4.20E-01 | 1 | |  | | *CD33* | 4.37E-01 | 1 | |  | | *BLNK* | 0.64587 | 1 |
| *CSF1R* | 4.40E-01 | 1 | |  | | *ARHGDIB* | 4.22E-01 | 1 | |  | | *ARHGDIB* | 4.45E-01 | 1 | |  | | *CYBA* | 0.662 | 1 |
| *P2RY12* | 4.46E-01 | 1 | |  | | *CD86* | 4.30E-01 | 1 | |  | | *CD84* | 4.60E-01 | 1 | |  | | *FYB1* | 0.67316 | 1 |
| *WDFY4* | 4.61E-01 | 1 | |  | | *PTPRC* | 4.41E-01 | 1 | |  | | *ADAM28* | 5.00E-01 | 1 | |  | | *LY86* | 0.69785 | 1 |
| *CD74* | 4.62E-01 | 1 | |  | | *TLR1* | 4.52E-01 | 1 | |  | | *FPR1* | 5.04E-01 | 1 | |  | | *CX3CR1* | 0.70983 | 1 |
| *CH25H* | 4.68E-01 | 1 | |  | | *ADAM28* | 4.69E-01 | 1 | |  | | *SUSD3* | 5.12E-01 | 1 | |  | | *LCP1* | 0.715 | 1 |
| *CMTM7* | 4.74E-01 | 1 | |  | | *HLA-DPA1* | 5.18E-01 | 1 | |  | | *LCP1* | 5.19E-01 | 1 | |  | | *RHBDF2* | 0.722 | 1 |
| *TLR2* | 4.89E-01 | 1 | |  | | *HCLS1* | 5.25E-01 | 1 | |  | | *CYTH4* | 5.47E-01 | 1 | |  | | *FCGR2A* | 0.72834 | 1 |
| *HLA-DMB* | 4.98E-01 | 1 | |  | | *IGSF6* | 5.66E-01 | 1 | |  | | *CMTM7* | 5.61E-01 | 1 | |  | | *FGL2* | 0.73 | 1 |
| *C1QA* | 5.04E-01 | 1 | |  | | *LY86* | 5.73E-01 | 1 | |  | | *ALG14* | 5.62E-01 | 1 | |  | | *TNFAIP8L2* | 0.73409 | 1 |
| *SLC7A7* | 5.05E-01 | 1 | |  | | *KCNQ1* | 5.88E-01 | 1 | |  | | *VAMP8* | 5.62E-01 | 1 | |  | | *KCNQ1* | 0.73905 | 1 |
| *TRAF3IP3* | 5.18E-01 | 1 | |  | | *ITGB2* | 5.92E-01 | 1 | |  | | *GPR183* | 5.63E-01 | 1 | |  | | *LAT2* | 0.7404 | 1 |
| *ALOX5AP* | 5.43E-01 | 1 | |  | | *CSF1R* | 5.96E-01 | 1 | |  | | *CYBB* | 5.82E-01 | 1 | |  | | *SUSD3* | 0.746 | 1 |
| *TMEM106A* | 5.45E-01 | 1 | |  | | *MYO1F* | 6.03E-01 | 1 | |  | | *ITGB2* | 5.97E-01 | 1 | |  | | *MS4A4A* | 0.753 | 1 |
| *SLC15A3* | 5.73E-01 | 1 | |  | | *TNFAIP8L2* | 6.05E-01 | 1 | |  | | *TMC8* | 6.03E-01 | 1 | |  | | *C1QA* | 0.75863 | 1 |
| *HCST* | 5.74E-01 | 1 | |  | | *SLC7A7* | 6.26E-01 | 1 | |  | | *CCR1* | 6.10E-01 | 1 | |  | | *C1QB* | 0.75863 | 1 |
| *HCLS1* | 5.78E-01 | 1 | |  | | *CCR1* | 6.29E-01 | 1 | |  | | *TMEM106A* | 6.31E-01 | 1 | |  | | *C1QC* | 0.75863 | 1 |
| *HLA-DMA* | 5.83E-01 | 1 | |  | | *C1QA* | 6.31E-01 | 1 | |  | | *SCIN* | 6.47E-01 | 1 | |  | | *APBB1IP* | 0.7685 | 1 |
| *HLA-DPB1* | 5.88E-01 | 1 | |  | | *ADAP2* | 6.33E-01 | 1 | |  | | *MS4A4A* | 6.56E-01 | 1 | |  | | *TRAF3IP3* | 0.81576 | 1 |
| *C1QC* | 6.01E-01 | 1 | |  | | *RNASE6* | 6.36E-01 | 1 | |  | | *MS4A7* | 6.70E-01 | 1 | |  | | *CD68* | 0.82533 | 1 |
| *IGSF6* | 6.05E-01 | 1 | |  | | *FYB1* | 6.39E-01 | 1 | |  | | *FCGR3A* | 6.74E-01 | 1 | |  | | *LILRB4* | 0.82841 | 1 |
| *RGS1* | 6.25E-01 | 1 | |  | | *HLA-DMB* | 6.42E-01 | 1 | |  | | *LILRB4* | 6.86E-01 | 1 | |  | | *RNASE6* | 0.82992 | 1 |
| *ADAM28* | 6.30E-01 | 1 | |  | | *FGL2* | 6.42E-01 | 1 | |  | | *P2RY13* | 6.97E-01 | 1 | |  | | *TLR1* | 0.83716 | 1 |
| *APBB1IP* | 6.39E-01 | 1 | |  | | *TRAF3IP3* | 6.55E-01 | 1 | |  | | *SLC2A5* | 7.42E-01 | 1 | |  | | *WDFY4* | 0.8567 | 1 |
| *CD86* | 6.65E-01 | 1 | |  | | *HLA-DMA* | 6.60E-01 | 1 | |  | | *CYTL1* | 7.43E-01 | 1 | |  | | *CH25H* | 0.914 | 1 |
| *DOCK8* | 6.66E-01 | 1 | |  | | *ALG14* | 6.70E-01 | 1 | |  | | *RGS18* | 7.48E-01 | 1 | |  | | *CD74* | 0.93278 | 1 |
| *C1QB* | 6.66E-01 | 1 | |  | | *CH25H* | 6.73E-01 | 1 | |  | | *MNDA* | 7.93E-01 | 1 | |  | | *CLEC9A* | 0.944 | 1 |
| *LAT2* | 6.71E-01 | 1 | |  | | *C1QB* | 6.88E-01 | 1 | |  | | *LAT2* | 8.04E-01 | 1 | |  | | *CSF1R* | 0.9679 | 1 |
| *HLA-DPA1* | 6.74E-01 | 1 | |  | | *RNASET2* | 6.94E-01 | 1 | |  | | *CD300A* | 8.14E-01 | 1 | |  | |  |  |  |
| *MS4A7* | 6.95E-01 | 1 | |  | | *C1QC* | 7.06E-01 | 1 | |  | | *CSF1R* | 8.25E-01 | 1 | |  | |  |  |  |
| *RNASE6* | 6.98E-01 | 1 | |  | | *IFI30* | 7.28E-01 | 1 | |  | | *RNASE6* | 8.42E-01 | 1 | |  | |  |  |  |
| *GPR183* | 7.38E-01 | 1 | |  | | *B3GNT5* | 7.32E-01 | 1 | |  | | *TLR2* | 8.61E-01 | 1 | |  | |  |  |  |
| *LRMDA* | 7.67E-01 | 1 | |  | | *CYTL1* | 7.79E-01 | 1 | |  | | *TRAF3IP3* | 9.10E-01 | 1 | |  | |  |  |  |
| *PTAFR* | 7.68E-01 | 1 | |  | | *SELPLG* | 7.83E-01 | 1 | |  | | *B3GNT5* | 9.59E-01 | 1 | |  | |  |  |  |
| *HK2* | 7.68E-01 | 1 | |  | | *APBB1IP* | 7.92E-01 | 1 | |  | | *PTAFR* | 9.70E-01 | 1 | |  | |  |  |  |
| *ALOX5* | 7.93E-01 | 1 | |  | | *CYBA* | 7.98E-01 | 1 | |  | | *ELF4* | 9.81E-01 | 1 | |  | |  |  |  |
| *RASAL3* | 7.98E-01 | 1 | |  | | *GPR183* | 8.55E-01 | 1 | |  | | *CLEC9A* | 9.85E-01 | 1 | |  | |  |  |  |
| *RGS10* | 8.03E-01 | 1 | |  | | *MNDA* | 8.63E-01 | 1 | |  | | *FGL2* | 9.92E-01 | 1 | |  | |  |  |  |
| *ADAP2* | 8.24E-01 | 1 | |  | | *SCIN* | 8.63E-01 | 1 | |  | |  |  |  | |  | |  |  |  |
| *OLFML3* | 8.42E-01 | 1 | |  | | *RHBDF2* | 8.81E-01 | 1 | |  | |  |  |  | |  | |  |  |  |
| *KCNQ1* | 8.59E-01 | 1 | |  | | *ALOX5AP* | 9.21E-01 | 1 | |  | |  |  |  | |  | |  |  |  |
| *RHBDF2* | 8.69E-01 | 1 | |  | | *C3* | 9.30E-01 | 1 | |  | |  |  |  | |  | |  |  |  |
| *FCGR1B* | 9.03E-01 | 1 | |  | | *PTAFR* | 9.44E-01 | 1 | |  | |  |  |  | |  | |  |  |  |
| *PIGP* | 9.31E-01 | 1 | |  | | *LAT2* | 9.56E-01 | 1 | |  | |  |  |  | |  | |  |  |  |
| *ARHGDIB* | 9.58E-01 | 1 | |  | | *VAMP8* | 9.77E-01 | 1 | |  | |  |  |  | |  | |  |  |  |
| Note: the significant genes common to both discovery and validation GWAS datasets are labeled with bold font. AD, Alzheimer's disease; IGAP, international genomics of Alzheimer's project; IMSGC, international multiple sclerosis genetics consortium; MAGMA, multi-marker analysis of genomic annotation; MS, multiple sclerosis; *Pc*, Bonferroni corrected *P* value. | | | | | | | | | | | | | | | | | | | | |
